# A Novel Cross-Disciplinary Multi-Institute Approach to Translational Cancer Research: Lessons Learned from Pennsylvania Cancer Alliance Bioinformatics Consortium (PCABC)

**Published:** 2007-06-08

**Authors:** Ashokkumar A. Patel, John R. Gilbertson, Louise C. Showe, Jack W. London, Eric Ross, Michael F. Ochs, Joseph Carver, Andrea Lazarus, Anil V. Parwani, Rajiv Dhir, J. Robert Beck, Michael Liebman, Fernando U. Garcia, Jeff Prichard, Myra Wilkerson, Ronald B. Herberman, Michael J. Becich, the PCABC

**Affiliations:** 1 Center for Pathology Informatics, Benedum Oncology Informatics Center, University of Pittsburgh Cancer Institute; 2 The Wistar Institute; 3 Kimmel Cancer Center of Thomas Jefferson University; 4 Fox Chase Cancer Center; 5 Abramson Cancer Center of the University of Pennsylvania; 6 Pennsylvania State Cancer Institute at Milton S. Hershey Medical Center; 7 Windber Research Institute; 8 Drexel University College of Medicine; 9 Geisinger Health System

## Abstract

**Background:**

The Pennsylvania Cancer Alliance Bioinformatics Consortium (PCABC, http://www.pcabc.upmc.edu) is one of the first major project-based initiatives stemming from the Pennsylvania Cancer Alliance that was funded for four years by the Department of Health of the Commonwealth of Pennsylvania. The objective of this was to initiate a prototype biorepository and bioinformatics infrastructure with a robust data warehouse by developing a statewide data model (1) for bioinformatics and a repository of serum and tissue samples; (2) a data model for biomarker data storage; and (3) a public access website for disseminating research results and bioinformatics tools. The members of the Consortium cooperate closely, exploring the opportunity for sharing clinical, genomic and other bioinformatics data on patient samples in oncology, for the purpose of developing collaborative research programs across cancer research institutions in Pennsylvania. The Consortium’s intention was to establish a virtual repository of many clinical specimens residing in various centers across the state, in order to make them available for research. One of our primary goals was to facilitate the identification of cancer-specific biomarkers and encourage collaborative research efforts among the participating centers.

**Methods:**

The PCABC has developed unique partnerships so that every region of the state can effectively contribute and participate. It includes over 80 individuals from 14 organizations, and plans to expand to partners outside the State. This has created a network of researchers, clinicians, bioinformaticians, cancer registrars, program directors, and executives from academic and community health systems, as well as external corporate partners - all working together to accomplish a common mission.

The various sub-committees have developed a common IRB protocol template, common data elements for standardizing data collections for three organ sites, intellectual property/tech transfer agreements, and material transfer agreements that have been approved by each of the member institutions. This was the foundational work that has led to the development of a centralized data warehouse that has met each of the institutions’ IRB/HIPAA standards.

**Results:**

Currently, this “virtual biorepository” has over 58,000 annotated samples from 11,467 cancer patients available for research purposes. The clinical annotation of tissue samples is either done manually over the internet or semi-automated batch modes through mapping of local data elements with PCABC common data elements. The database currently holds information on 7188 cases (associated with 9278 specimens and 46,666 annotated blocks and blood samples) of prostate cancer, 2736 cases (associated with 3796 specimens and 9336 annotated blocks and blood samples) of breast cancer and 1543 cases (including 1334 specimens and 2671 annotated blocks and blood samples) of melanoma. These numbers continue to grow, and plans to integrate new tumor sites are in progress. Furthermore, the group has also developed a central web-based tool that allows investigators to share their translational (genomics/proteomics) experiment data on research evaluating potential biomarkers via a central location on the Consortium’s web site.

**Conclusions:**

The technological achievements and the statewide informatics infrastructure that have been established by the Consortium will enable robust and efficient studies of biomarkers and their relevance to the clinical course of cancer. Studies resulting from the creation of the Consortium may allow for better classification of cancer types, more accurate assessment of disease prognosis, a better ability to identify the most appropriate individuals for clinical trial participation, and better surrogate markers of disease progression and/or response to therapy.

## Background

Biomedical researchers, geneticists, pathologists and tissue engineers depend on cancer biorepositories for the development and validation of new diagnostic or prognostic cancer markers, the understanding of basic disease mechanisms, and the evaluation of proposed therapeutic regimens or the creation of new bio-engineered materials or pharmaceuticals. Recent advances in translational research have resulted in a growing demand for specific, highly annotated human tumor tissues [1–3], and this demand has reinforced the importance of tissue banks as a major part of the necessary infrastructure of any region seeking to become a force in medical biotechnology.

Although virtually all major academic cancer centers—in Pennsylvania and across the nation— have research tissue banks, it is still difficult for most cancer researchers to find sufficient and well annotated tumor tissues that are necessary to begin and complete their work. This is because existing banks are beset by a number of major limitations: (1) the number of tissue specimens at a given site is limited, (2) sharing of information is constrained by confidentiality and de-identification issues, (3) the annotation process is complex and expensive, and (4) each tissue bank may define and apply annotation differently. The result is that access to well documented tissue specimens, using normalized descriptors, remains one of the most important limitations to cancer biomarker research[4].

In 1998, Pennsylvania’s leading cancer centers organized the Pennsylvania Cancer Alliance (PCA) and set as one of its first goals the task of communicating to Commonwealth legislators and the Governor the need to allocate on a long-term basis a substantial portion of the funds from that year’s national Master Settlement Agreement with the tobacco industry[5] to research and prevention of diseases directly linked to tobacco use, including cancer. In 2001, the PCA’s efforts led to the Commonwealth legislators’ and the Governor’s decision to approve Act 77 [6], to allocate 19% of Pennsylvania’s tobacco settlement money for biomedical research initiatives that would be sponsored by the PA Department of Health’s Commonwealth Universal Research Enhancement (CURE) program [7]. The details of the allocated funds to this project are discussed below.

The Pennsylvania Cancer Alliance Bioinformatics Consortium (PCABC, http://www.pcabc.upmc.edu) is one of the first major project-based initiatives stemming from the PCA, which was funded for three years by the Commonwealth. The objective of the PCABC was to initiate a prototype system for a Bioinformatics, Data Warehouse and Biorepository Project, by developing: (1) a statewide data model for bioinformatics; (2) a statewide repository of serum and tissue samples; (3) a data model for biomarker data storage; and (4) a public access website for disseminating research results and bioinformatics tools. The seven institutions comprising the Consortium have cooperated closely to (1) explore the opportunity for sharing clinical, genomic and other bioinformatics data on patient samples in oncology, (2) for the purposes of synergistically cooperating to share informatics and other expertise, and (3) of developing collaborative research programs across cancer research institutions in Pennsylvania. In many cases, clinical specimens reside in individual departments in each institution. These specimens are essentially unknown to the comprehensive research community and thus underutilized. The Consortium’s vision was to provide a virtual repository of clinical samples located in various departments across the state, supported by a common and well standardized informatics platform, in order to make them available for research. In addition, one of our primary goals was to facilitate the identification of cancer-specific biomarkers and encourage collaborative research efforts among the participating centers. Details of this “virtual biorepository” for melanoma, breast and prostate cancers, tools for data mining, and the imaging storage and analysis software that make up the bioinformatics platform will be discussed.

## Methods

### Participating institutions

The PCABC was created to explore the opportunity for sharing clinical, genomic and other bioinformatics data on patient samples in oncology, for the purpose of developing collaborative research programs across cancer research institutions in Pennsylvania. It is hoped that this activity will serve as a model for other regions, and be able to include multiple institutions in an enhanced research environment. The organization was initially comprised of University of Pittsburgh Cancer Institute, Fox Chase Cancer Center, Abramson Cancer Center of the University of Pennsylvania, Pennsylvania State Cancer Institute at Milton S. Hershey Medical Center, Fox Chase Cancer Center, Kimmel Cancer Center of Thomas Jefferson University, and The Wistar Institute. The Consortium has recently added new partners, that include the Geisinger Health System, Windber Research Institute, and Drexel University College of Medicine, and it is considering further expansion to include institutions from neighboring states.

The Consortium has access to cases and research facilities from a variety of medical care settings that include major cancer centers, academic medical centers, research institutions, as well as rural community-based healthcare networks. This varied access allows the Consortium to encompass the wide diversity of patients with cancer that reflects the demographics of the State of Pennsylvania and the surrounding areas and thereby expanding beyond the patient populations of the Cancer Centers’ component of the PCABC.

### Organization of the consortium

**Figure 1.**
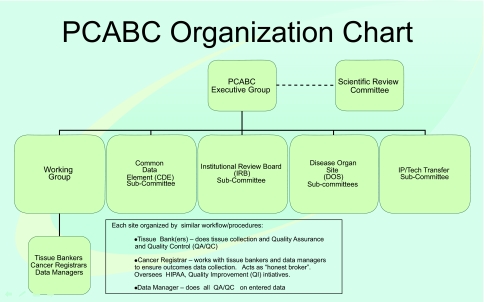
**Organization Chart.** The PCABC organization chart.

The Consortium is governed by an *Executive Group* made of PCABC institute leaders, who oversee and guide Consortium’s development and ongoing activities. Figure 1 depicts the organizational chart of the Consortium. The skill set of each committee and types of roles that make up each committee are summarized in Table 1. The *Executive Group* has delegated tasks to several subcommittees that included:

#### Disease/organ site sub-committees

Initially, a subset consisting of three target cancers: breast and prostate cancers and melanoma were selected to develop and implement a prototype system for evaluation of the complex elements necessary to enable the Consortium to succeed, as well as leveraging each of the member institutions’ well established research programs. The extension to additional cancers, as prioritized by the Executive Group, will follow the successful implementation of the prototype system by all members of the Consortium. In addition to storing the tissue samples with the needed clinical annotations, the Consortium aims to perform biomarker tests on the list of biomarkers compiled and agreed to by the Disease/Organ site sub-committee members. The Master List of purposed biomarkers is provided as supplemental file #1 with this manuscript and is a compilation of biomarkers suggested by investigators from all member institutions. The PCABC asked the research community to help the Consortium to determine which markers would be of interest strategically to compliment studies already underway or to add to banks of information already accumulated on a particular marker. For each biomarker submission, the PCABC asked the investigator to: 1) name the marker and primary cellular function or pathway involved in its impact on the neoplastic cell, 2) provide a short description of the background work done on this marker and why it would be important or at least worthwhile to pursue it as a therapeutic and/or diagnostic marker, and 3) designate the organ system site of most interest for exploring this marker explored, and the rationale behind its selection. The logic was to ask these well-published disease site experts and program leaders from each center to work with the Consortium in identifying biomarkers that have the most promise, with the intention of targeting those biomarkers first and foremost. The Master List of purposed biomarkers is a “work in progress,” as each disease/organ sub-committee assembles to ration and prioritize the biomarkers of interest. This “Biomarker Master List” will be used as a guide for all research proposals requesting biospecimens from the Consortium. The Consortium does not expect that each organ site and biomarker will be studied by each institution. Instead, a scientific review group will be assembled as part of the committee structure of the PCABC, which will decide which biomarkers are performed on which tumor sites and by which partnering organizations. If certain biomarkers are able to be tested at multiple sites, the scientific review group along with the executive committee will decide which institution will be the preferred testing site and a second testing site for validation. Experimental results and biomarker data generated from the tissues utilized from the Consortium biorepository will be requested from the researchers at the member sites for inclusion within the database as described below.

#### Intellectual property (IP)/technology transfer sub-committee

This group has the task of reviewing the issues surrounding how to deal with any intellectual property that develops as a result of the Consortium’s efforts. This group is identifying how the legislative language [6, 8] that dictates the grant award affects IP. The committee will begin to define potential IP and has devised a framework inter-institutional agreement to deal with the technology transfer needs of the Consortium. Additionally, to protect each institution, the Consortium has carefully studied the biomarker list and their IP characteristics, to ensure that the IP protected markers will be performed by the institutions offering them and that there will be agreements between the institutions to protect both the patients’ privacy and confidentiality when these biomarker assays are performed. This committee includes general counsel, business development directors, technology transfer directors, and faculty with experience in this important and complex area.

#### Institutional review board (IRB) sub-committee

This sub-committee had the initial task of: a) reviewing current practices within each center regarding sharing of banked tissue, b) comparing current patient informed consent samples, and beginning to develop a common standardized patient consent form and IRB protocol template, that all centers will use when collecting samples for analysis by the Consortium, and c) communicating with each center’s tissue banks to determine what tissue types and the number of samples that are sharable immediately. In addition, it is the Consortium’s intention that in subsequent years of the program, cross institutional IRB will be generated to review specific uses of these tissues between institutions. For example, if a group at the University of Pittsburgh wanted to partner with the University of Pennsylvania in a melanoma project, a separate IRB that would address tissue specimens collected at both institutions and the exact nature of that research that would be performed will be the subject of a subsequent IRB. Hence, the initial proposed work will focus on allowing the PCABC partners to bank and share de-identified information through a central database in the goal of providing this information for investigators across all member institutions that are part of this Consortium. The PCABC is fortunate to have on this committee an IRB chairman, a HIPAA regulatory specialist and a tissue bank director who can help direct the dialogue and provide thorough guidance to the Executive Group.

#### Common data elements (CDE) sub-committee

This important sub-committee was one of the first to be organized, in order to decide what information on specimens and assays will be tracked. Standardization and compatibility will be paramount to successful data sharing. To that end, the subcommittee has broken down the task into nine modules: Patient demographics, family history, clinical history, genomic information, patient consent issues, pathology report, tissue sample descriptors, outcomes and biomarkers. The intent of this modularization is to enable generalizable CDEs to be commonly developed across tissues/organs/cancers and to facilitate the swapping in/out of specialized modules as they are needed. However, because one of the initial goals was to enable the rapid implementation of a tissue and data repository for the Consortium in support of the biomarkers focus, the sub-committee focused the CDE development on the modules that allowed annotation of biospecimens. While utilizing and learning from the experiences of several others groups, the CDE sub-committee particularly took into consideration the experiences of the Cooperative Prostate Cancer Tissue Resource [CPCTR, see http://www.prostatetissues.org] [9, 10] as well as established open source standards including the Cancer Staging Manual[11] from the American Joint Committee on Cancer, the Data Standards for Cancer Registries from the North American Association of Central Cancer Registries [12], the College of American Pathologist’s Cancer Checklist[ 13], and other biorepository specific CDEs that were available through the National Cancer Institutes Center for Bioinformatics [14, 15]. This committee is comprised of molecular pathologists, microbiologists, oncology informatics pathologists, genomics core directors, and others who bring a wealth of experience in collaborative data collection.

#### Working group

PCABC’s Working Group infrastructure consists of key personnel from each institution, which includes data managers, cancer registrars, tissue bankers, database administrators and project coordinators who are responsible for day-to-day operations at individual sites as well as reporting progress to the central project coordinator.

*Data Managers* from each participating site act as the communicators and liaisons to the project coordination center. Responsibilities include managing the local site’s data entry and the site’s own de-identification link back to the patient identified data. A Data Manager is the point person for PCABC to communicate with regarding all clinical tissue and data collections and interactions with tissue bank directors and cancer registry managers from their respective institution. Data Managers will eventually be responsible for quality assurance standards within their center as they relate to data entered into the PCABC database.

*Data Entry Personnel* are any staff member who has access to the PCABC database and has the responsibility to review and/or enter data for their institution. These individuals review and disseminate data from various internal sources. Data entry personnel have the responsibility of entering the data accurately into the PCABC database. Oftentimes, they work within the tissue bank or the tumor registry department of the participating center, as they have the training and experience necessary to provide accurate and timely entry of critical data. Each institution’s tissue bank(ers) is responsible for collection and quality assurance and quality control of the biospecimens shared with the Consortium. The Cancer Registrars work with tissue bankers and data managers, to ensure outcomes data collection, and act as “honest brokers” (see below) as well as assist in quality improvement initiatives with data annotation processes.

### Human subjects protection—the honest broker concept

Each PCABC institute collects tissue and data locally according to the local and institutional guidelines and procedures. Tissue is stored locally but data management is done centrally. Each member organization has developed its own local protocols, including consent language describing its procedures to protect the confidentiality and privacy of human subjects [16, 17] and has obtained local/institutional IRB approval for all PCABC activities.

The institutions that make up the Consortium ensure protection of patient identity through “The Honest Broker Concept”[18]. An “honest broker” or “tissue bank trustee” acts as a well-defined barrier between the clinical environment (in which fully identified confidential patient information is routinely exchanged as part of medical care) and general research community (in which all information must be completely de-identified). In its simplest form, the honest broker is not part of either the clinical or research team and is the only person or organization that can link research identifiers and clinical identifiers. Although it differs between each PCABC organization, provisions are in place for the data managers, tumor registrars, tissue bankers, or local database administrators at the local PCABC sites to act as honest broker(s). Use of the honest broker system allows each organization to control the de-identification process and places responsibility and accountability for that process. Personal and clinical identifiers (names, medical record numbers, etc.) are limited to the clinical space while research identifiers (i.e. “subject 12432” are never tied to the personal or clinical identifies except in the honest broker’s code book. This concept differs from anonymization, which is a one way process of removing the linkage between personal identifiers with research identifiers.

This concept is implemented by designating at least one tissue bank trustee from each institution to the central PCABC coordination site. For example, at one institution, a tumor registrar is designated as the tissue bank trustee since tumor registrars, by the nature of their job and by federal mandate already have access to clinical information on cancer patients and do not have access to the results of research data for tissue bank samples. The trustee is the only person who can link a patient with the tissue bank number that identifies that patient. The trustee system ensures that new clinical outcome information can be added to a file identified only by a code number, not a name. In rare but possible circumstances, when critical research data becomes available and it becomes medically necessary to inform the patient or his survivors, the honest-broker system provides for an effective mechanism to ensure that the critical information reaches the interested party in a timely manner.

### Central database design

The PCABC central database was modeled and expanded from the work of the Cooperative Prostate Cancer Tissue Resource (CPCTR) [9, 10]. The system is relational database designed as a virtual bio-repository that gathers information on banked tissues and patients in research trials —including clinical and molecular (gene and protein data)—from each of the Consortium member institutions. This data will be de-identified at the local institutions and made available in a central data warehouse for visualization and query.

The workflow for entering data into the virtual bio-repository has been as follows:

1. The local (physical) tissue bank identifies cases appropriate for inclusion in the Consortium’s virtual bio-repository (warehouse).
2. The local (physical) tissue bank pre-processes data on these cases. The most important component of pre-processing is de-identification. All de-identification occurs at the local banks. No identifiable data is sent to the virtual biorepository (warehouse).
3. De-identified data are entered into the warehouse through a web site. The data entry web site uses radio buttons, combo boxes and other highly constrained data elements.
4. The local (physical) banks label each case with a de-identified number. This number is used to link the information in the warehouse to the cases in the local banks. The linkage codes are stored locally, using appropriate electronic and physical safety measures. Only the local banks have access to these linkage codes. The database also keeps a system generated key that is tied to the de-identified number that protects and secures the data links as an additional security measure.
5. The warehouse contains very minimal demographic data and complies with all HIPAA requirements.
6. Access to the data entry application is controlled by user name and password. Cases entered into the virtual bio-repository are scanned for logical errors (e.g. first recurrence before diagnosis etc.).

The workflow for querying the warehouse is as follows:

Initially, access is limited to members of the Consortium using a username and password system. The data manager at each facility is able to provide user names and passwords for approved researchers at that institution. The query tool accesses the central database through a highly constrained “click and point” interface. The data in the warehouse will eventually will allow queries on many of the approved data elements. However, the specificity of the data returned will depend on the user’s profile.

There are three user profiles as follows:

1. *The Public Query tool* is available to the general public and is accessible through the Consortium’s web site [http://www.pcabc.upmc.edu]. The output display of a public query is the accrued number of cases, specimens and blocks in the database that meet the criteria of the query and general statistics on a limited number of data elements. It is designed to allow interested investigators to see if there are enough tissue specimens available through the PCABC that meet their specific requirements.
2. *Approved Investigator Query tool* is password protected tool and is distributed to those research investigators who have approved research protocols within the PCABC or its member institutions. It allows users to refine and compile case lists for their application and also to mine and modify the default data views on the cases they received biospecimens from the Consortium. It shows all the annotations on the data associated with each case through multiple pre-defined views of the data set.
3. *Data Manager Query tool* is a password protected tool, available only for the internal PCABC members. It is meant for data managers to address QA issues regarding the data collected from a specific institute and generating a tissue disbursement list. The main difference between this viewer and the Approved Investigator Tool is that this tool allows the user to identify the institution from which the cases originated (i.e. UPCI, FCCC, PSU, etc.).

### Application of use of material

Instructions on how to apply for specimens are available by contacting the PCABC central project coordinator. Should a researcher find tissue samples that may be useful in ongoing or proposed research from the PCABC query tool, the investigator is requested to first submit a letter of intent (LOI) indicating their proposed study and tissue requirements. The LOI is reviewed by the Executive Group or the Consortium’s Scientific Review Committee, consisting of an independent group of experts for each disease/organ category. Tissues are not released until after:

- IRB approval of the study at the researcher’s institution—for patient safety of purposed research.
- IRB approval of tissue collections at the tissue bank institution—for patient safety of biospecimens banked.
- Approval of the Consortium’s Scientific Review Committee—to determine if the proposed research has validity and justifies the use of potentially valuable tissue resources from the Consortium.
- Approval of the local (physical) tissue bank - As “owner” and guardian of the tissue specimen (the local banks may have their own review committees or may wish to collaborate with the researcher).

## Results

The PCABC has become a model of how to effectively partner across multiple and complex research institutions, to achieve the common goal of building a statewide or regional bioinformatics network. Currently, there are over 80 individuals who are members of this unique PCABC partnership, which is continuously looking to expand to include new partners so that every region of the state as well as neighboring states can contribute and participate. This has created a network of researchers, clinicians, bioinformaticians, cancer registrars, program directors and external corporate partners - all working together to accomplish a common mission.

**Figure 2.**
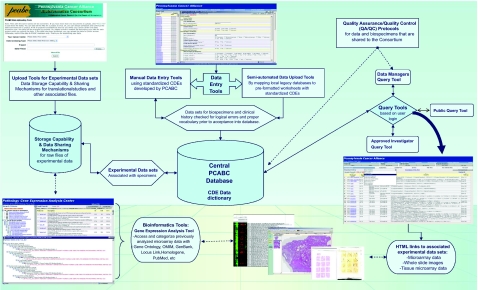
**PCABC Database Tools.** The tools and applications developed by the Consortium and data warehouse’s capabilities are highlighted.

The various sub-committees have developed a common IRB protocol template, common data elements for standardizing data collections for three organ sites, intellectual property/tech transfer agreements, and material transfer agreements that have been approved by each of the member institutions. This was the foundation work that has led to the development of a centralized data warehouse that has met each of the institutions’ IRB/HIPAA standards. The data warehouse has web-based interfaces for both data entry and query capabilities. The data entry is either done manually over the internet or semi-automated batch files through mapping of local data elements with PCABC common data elements. A second, more important part of the warehouse is its ability to associate supplementary data sets, such as high throughput molecular data or other files, such as whole slide images of paraffin sections, by linking it with the originating specimen (and patient). Although these supplementary files are not fully integrated into the biospecimen warehouse, hyperlinks to separate databases or applications are provided for those cases that are known to have any additional files. The group has also developed a central web-based tool that allows investigators to share their translational (genomics/proteomics) experiment data on research evaluating potential biomarkers at a central location on the Consortium’s website [http://pcabc.upmc.edu/data/acctools]. In some cases, access to raw experimental data would be available to only internal PCABC members or would require communication and collaboration with the data originator. The many tools and capabilities of the PCABC data warehouse are highlighted in Figure 2.

The design of the central database is developed to have normalized subset of standard clinical, pathological, outcomes and molecular and other biomarker descriptors, to enable Consortium members to utilize the warehouse to substantially complement their own local data repositories while making tissue specimens optimally available for use in translational and clinical cancer research.

### Case accruals for the PCABC biospecimen data warehouse

**Figure 3.**
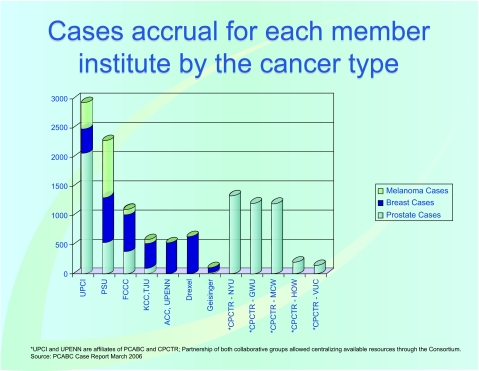
**Case Accrual Rate.** The total accrual rate of cases for each member institute by the cancer type.

Access to well qualified tissue samples is fundamental to biomedical research. PCABC developed this site for the exchange of de-identified information on consented tissue samples available to Pennsylvanian and national researchers. This site is updated each morning [http://www.pcabc.upmc.edu/main.cfm?dis=pqe]. Figure 3 shows the accrual rate of cases for each member institute by the cancer type.

**Figure 4.**
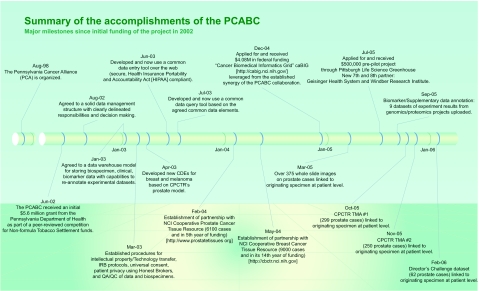
**PCABC Timeline.** Summary of the accomplishments of the PCABC since initial funding of the project in 2002.

**Figure 5.**
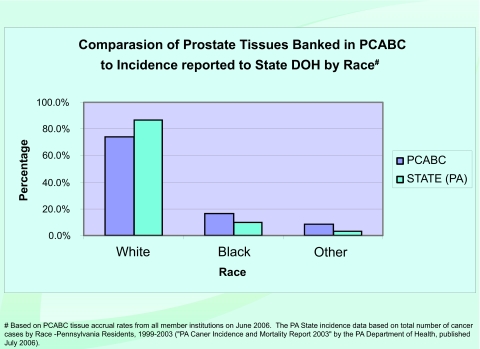
**Percentage of Prostate Tissues Banked compared to Incidence Reported in Pennsylvania.** Comparison of Prostate Tissues Banked in PCABC to Incidence reported to State DOH by Race.

**Figure 6.**
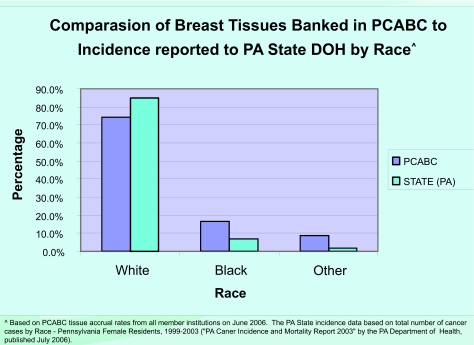
**Percentage of Breast Tissues Banked compared to Incidence Reported in Pennsylvania.** Comparison of Breast Tissues Banked in PCABC to Incidence reported to State DOH by Race.

**Figure 7.**
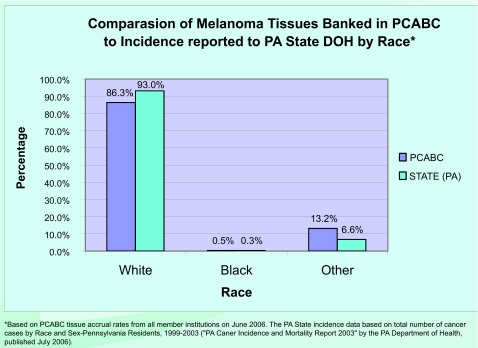
**Percentage of Melanoma Tissues Banked compared to Incidence Reported in Pennsylvania.** Comparison of Breast Melanoma Banked in PCABC to Incidence reported to State DOH by Race.

**Figure 8.**
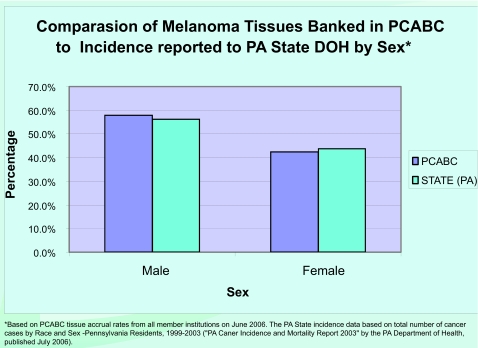
**Percentage of Melanoma Tissues Banked compared to Incidence Reported in Pennsylvania.** Comparison of Melanoma Tissues Banked in PCABC to Incidence reported to State DOH by Sex.

As of January 2007, the “virtual biorepository” has over 60,000 individual annotated samples from 12,734 cancer patients available for research purposes. The Prostate Cancer Database currently holds information on 7327 cases (including 9424 specimens and 46752 annotated blocks and blood samples). The Breast Cancer Database currently holds information on 3645 cases (including 4707 specimens and 10325 annotated blocks and blood samples). The Melanoma Database currently holds information on 1762 cases (including 1591 specimens and 2953 annotated blocks and blood samples). These numbers continue to grow, and plans to add more tumor sites are in progress. The accomplishments of the Consortium, since funding of this project in 2002, are summarized in Figure 4. A comparison of tissues banked with the cancer incidence data (race and sex) from the Pennsylvania Department of Health is described by Figures 5–8.

### Information and resources available from the consortium

The PCABC website contains additional information about the Consortium, including a frequently asked questions (FAQ) section. Additionally, many of the key resource documents have been provided as supplemental files with this manuscript, such as:

- The latest version of the Biomarker Master List (additional file #1)
- The Intellectual Property /Technology Transfer Agreements (additional file #2)
- Biomarker Disclosure Form (additional file #3)
- Template IRB Protocol for PCABC project (additional file #4)
- Template IRB Protocol for the Honest Broker system (additional file #5)
- Universal Consent Template (additional file #6)
- Material Transfer Agreement Template (additional file #7)
- The freely available CDEs developed for breast, prostate, and melanoma by the Consortium can be downloaded from its public database link on the PCABC website. (additional files #8–10)

Alternatively, additional information can be obtained by contacting the project coordinator listed on the Consortium’s website [http://www.pcabc.upmc.edu].

## Discussion

### Repository model

Each institution of the PCABC collects and stores specimens locally within their own tissue banks, while the data and information related to these specimens are maintained in a centrally located bioinformatics and data management system that is accessible by the members of the network. Although the Consortium has been successful to date, the implementation of this decentralized collection and storage with a centralized data management model has resulted in several limitations that are discussed in the subsequent sections.

### CDE issues

Creating the CDEs for the three initial disease types was modeled from the work of the Cooperative Prostate Cancer Tissue Resource. After their successful implementation of the prostate CDEs, the Consortium merely replicated the prostate CDEs and modeled the same format for the breast and melanoma CDEs within the data warehouse. However, as data entry began for the two later disease types, the difficulties of modeling them after the prostate CDEs surfaced. In particular, the prostate model allowed only one patient with one primarily disease to be entered along with associated tissue accessions pertaining to the primary disease; since once a prostate is removed, the patient does not have another primary prostate cancer. However, for other disease sites such as breast and melanoma, data on additional primary tumors of the same disease type cannot be accommodated within a single patient’s record using the system developed. The central data warehouse was a relational database, thus how data was entered and queried was limited by the constraint put by the design. The CDEs were arranged as:

- Case or Patient Identification number
  ○ Consent Information
  ○ Demographics and History
  ○ Progression and Outcomes
  ○ Clinical and Pathological Staging Information
- Treatment
- Biomarker data (i.e. PSA)
- Tissue Accession
  ○ Accession Level Pathology data
  ○ Block/Aliquot Level Pathology data

After initial implementation, several limitations were faced when data entry was done on the patients who had multiple primaries from the same disease/organ site. For example, the current design only allowed the input of staging information once per patient record. To bypass this issue, separate records with the patient identification number assigned as 12345-A, 12345-B, etc. were entered into the database for cases that had multiple primary diseases from the same organ site. However, this solution presents its own set of issues. For instance, when a patient’s vital status is presented as “dead”, the question of which of the two primaries was the cause of death is raised. Another issue is when updating this key information or other follow up data, multiple files of the same patient would have to be updated for a single piece of information. Furthermore, the ability to search for cases with multiple primaries would not be integrated, thus lessening the true value of such cases for research studies.

### IRB issues

Members of the IRB sub-committee addressed any privacy issues that might be of concern at individual IRB committees. Developing a standard IRB protocol that would be common at each institution for the entire project was a key step for the Consortium, especially as it related to data sharing in the PCABC biorepository data warehouse. Several institutions encountered delays in getting its IRB to approve the goals of the project. The primary concerns of the IRB were 1) how and why were data de-identified instead of annonymized? 2) Who has ownership of the tissue? 3) Who was allowed the usage of tissues/data from the Consortium? And 4) how was it determined who was given tissues/data from the Consortium?

In order to resolve these concerns, the project coordinators conducted on-site meetings and conference calls with individual site IRBs, to address their concerns as well as educate and train members of the Consortium on the role of cancer registries, training of HIPAA guidelines, and implementation of the Honest Broker system [18]. It was explained to the IRBs that it was critical to have cases that were entered into the Consortium to be de-identified so that follow-up information can be added as well have the ability to re-annotate those cases with experimental data sets when tissues were used for research studies. It was also noted that the ownership of the tissues is always retained with the “tissue bank(s)” of each institution. Full governance for all tissue and its associated data was in place within the Consortium and distributed to only those 0requests with all the appropriate approvals mentioned above were obtained. Furthermore, the creation of the multiple query views based on the user’s profile was directly related to the concerns of the IRB. An issue was raised that although there was a governance place on how tissue was requested, how was access to the data warehouse limited to potential users. Besides the privacy issues, the concern was that having detail information on individual cases would introduce case selection bias for potential research studies.

### Data annotation methods—manual vs. automated

Originally, the partners envisioned primarily a manual annotation methods by using web-based data entry interfaces, but we quickly discovered this was too time consuming as many centers had “legacy” databases that required researching and compiling archived data. Developing standardized parsing and semi-automated data entry methods with those institutions by adapting data mapping methods to created flat files that allowed alternative methods of batch uploads to the central data warehouse. However, the ability to implement these semi-automated techniques varied at each institution depended on the IT (information technology) support staff availability. Some institutions had large sophisticated legacy databases as well as access to other hospital base systems, such as tumor registry systems; those programmers and database administrators were able to map the required PCABC data elements with their internal data sources. Some institutions had minimal informatics infrastructure to support their homegrown databases (i.e. Access), with limited or no domain knowledge and expertise. These scenarios required the central PCABC database administrators to educate those institutions regarding the required format of the standardized export files. Of note, those institutions where no previous tissue bank existed, the data entry tool allowed for quick deployment of informatics tools that not only helped with case accruals for the Consortium, but also for alternative ways of managing their local tissue bank.

### Issues of semantics

The legacy databases lack the ability to exchange information and semantic interoperability, which is the ability to understand and use the information once it is received by other systems. Another problems is that the panoply of ways that similar or identical concepts or data are described by different users even within the same institution. For example, a data element called “grade of tumor” can be collected using various formats (e.g. some collect “grade-1, grade-2, and grade-3” vs. others who collect “low grade, intermediate grade, and high grade”). Such inconsistency in data descriptors makes it nearly impossible to aggregate and manage even modest-sized data sets in order to be able to ask basic queries. Moreover, these systems are not uniform and flexible as well as incapable of performing easy transfer of information and unambiguous interpretation of the information once it arrives into a central database.

However, our current database is based on such a bioinformatics model that aids in developing and conveying the semantic interoperability of the data system by describing the common data elements in the form of metadata or data descriptor (about the content, quality, condition, and other characteristics of the data) using controlled vocabulary and ontology, in order to make the data understandable and sharable for end-users and flexible for the system. Each common data element is associated with an object or concept, attribute, and valid value(s). For example, “patient age at diagnosis” is the CDE that is made up of “patient” (object), “age at diagnosis” (property) and the representation (value domain) in “years”. Thus, in order to share quality data into the PCABC central database, the data collectors at each site need to understand what exactly needs to be collected, as has been agreed by the members of CDE subcommittee. Specifically for each of the approved CDEs, the data collectors need to know: 1) the fundamental definition of the data element (i.e. date of diagnosis), 2) how that data element will be collected (e.g. 11/2003 vs. Nov. 2003 vs. 11/03, etc), 3) what are the consensus acceptable values or codes are for the data element (e.g. precise date of birth, not calculated from clinical records where the “patient appears to be a well developed 75 year old”), and 4) what the acceptable data format is for inclusion into the central database (e.g. dates as integers not character strings). Although the concept of formalized metadata is fairly straight forward, it has been rarely incorporated by clinical and research groups building databases[19].

The CDEs developed by the Consortium are ISO/IEC 11179 compliant (International Standards Organization / the International Electro technical Commission) [19,20] that means it defines a number of fields and relationship for metadata registries including detailed metamodel for defining and registering items, of which the primary component is a data element. The PCABC uses CDE standards and metadata that are defined by the consortium members of participating institutions. Furthermore, the Consortium members, through their work in the caBIG initiative[21], are working to enhance the database as a similar object-relational model as PCABC as well as some of the CDEs by using pre-defined controlled vocabulary systems like those in NCI Thesaurus[22] (NCI Thesaurus is a knowledgebase that contains the working vocabulary used in data systems covering clinical, translational and basic research as well as administrative terminology) with semantically integrated and globally accepted valid values for each data elements. PCABC has the Oracle platform with internal object model, whereas caBIG creates its own object model and forms the data standard repository taking package, class, attribute, and data type into consideration on a Java template. This differs from the current PCABC practices, in that, caBIG attempts to use these “globally” accepted CDE standards and metadata descriptors recommended by multiple voluntary organizations and groups from the research community that will allow all NCI-sponsored research to share data more easily. By taking this new approach, greater flexibility will be given to individual institutions with the current limitations placed in data collection methods and work flow. As another example, for the CDE “Patient race”, the object is “patient”; property is “race” and the valid value or representation of the metadata (“Caucasian”, “African American”, “Asian”, etc). If legacy databases from one institution uses “African American” and another institute uses “Black” as a valid value for the “race” field and both their CDEs and metadata are properly mapped to the central database, then when researchers query for “African American” cases in the Consortium’s user interface, the result would display the total cases (“African American” plus “black”) available from both institutions, because both terminologies are semantically integrated with the accepted system. Hence, the overall advantage of a distributed or federated model like caBIG over a centralized model like PCABC include the shared responsibilities of individual institutions for services and implementation of the required standards and vocabulary that would foster data sharing effortlessly. Furthermore, the centralized PCABC architecture has a data sharing model inbuilt that controls the various participating institutions. The implementation of federated systems do not have inbuilt data sharing models, but rather services that run as external grid services, which connects with local databases to establish such data sharing mechanisms. The importance of semantic integration cannot be over emphasized.

### Data integration of high throughput datasets

Although more work needs to be done, the Consortium’s current database design has limited integration capabilities of supplementary data sets, such as high throughput molecular data or other files, such as whole slide images of paraffin sections, by linking it with the originating specimen (and patient) [23–25]. Unlike classical tissue annotation and clinical data, high throughput techniques generate a large amount of well formatted and semantically integrated data, typically for a relatively small number of tissue specimens. These data require significant computation analysis, usually using several different approaches. The development of new tools for analysis of molecular research data is presently an area of intense research and focus of other major initiatives in the vocabulary workspace domain of caBIG infrastructure [21]. Data standards for high throughput data are only now being developed and there is no single standard that meets the needs of all researchers. Development of a metadata repository may resolve this issue.

### Tissue/data ownership issues

Numerous annotated tissue repositories already exist at most large academic institutions and cancer centers. In order to make them available to a wider research community, much work needs to be performed to educate those tissue banks about the benefits of sharing their resources in collaborative projects such as the PCABC. One success of the PCABC was to take an inventory of existing projects that possibly could partner with the Consortium. This led to the partnership with the CPCTR, with its data being shared in the PCABC data warehouse. In addition, there are multiple collections of similar disease/organ types within a single organization that individuals are either not aware of or cannot access. Furthermore, many tissue bank-focused projects do not consider the vast resources of paraffin archives that are available for use, that is housed in many academic pathology departments [26]. In order to resolve these issues, project leaders from many developing and ongoing collaborative projects [21, 27–36] need to provide incentives to all parties, especially to all those tissue banks that already exist, to collaborate and to build upon what they have learned without “re-inventing” much of the infrastructure.

### Funding

The key feature to improve the management and prevention of cancer is the novel ideas that come from research, and the pace of research is directly related to the availability of advanced technology and top talent, together with the infrastructure needed to financially support them. A successful research effort therefore requires a steady, reliable commitment of support, and the tobacco settlement offers Pennsylvania an opportunity to make that crucial long-term commitment and to measure the results of its investment in lives saved.

On November 16, 1998, the Commonwealth of Pennsylvania and 45 other states and territories entered into an historic settlement with the tobacco industry [5]. Under the terms of the Master Settlement Agreement, the tobacco industry will pay the states $206 billion over 25 years. Pennsylvania’s share of that settlement is valued at more than $11.2 billion [37]. This settlement presents Pennsylvania with an opportunity to invest in research that will reap benefits for all its citizens for generations. The PCABC was awarded $5.5 million for a three year project as one of the first unique collaborative projects seeking to promote cancer research throughout the state by utilizing the funds of the tobacco settlement [38]. The Commonwealth’s goal of funding such a project was to create a bioinformatics infrastructure for a biorepository of body fluids such as blood, serum, urine, etc. and tissue samples which, in turn, put the Consortium in a better position as a group to compete for additional funding to advance cancer and bioinformatics research. In addition, the Commonwealth hoped to facilitate collaborations not only among the researchers who are members of the Consortium, but also with outside collaborative partners such as pharmaceutical and biotechnology companies, the Life Sciences Greenhouses [39], and researchers and institutions outside of the consortium to energize economic growth throughout the state.

According to a study produced by the Tripp Umbach firm [40], since its start in 2001, the CURE program has provided $298 million for medical research in Pennsylvania using the tobacco money. That has resulted in a $544 million boost to the state’s economy—a nearly two-to-one return on investment. More than 4,000 high paying jobs were created or sustained in every sector of the economy through CURE funding, and $32 million in new tax revenue for the state was generated. In addition, many members of the PCA successfully leveraged the CURE funds to secure $138 million in new federal medical-research funding as well as $500 thousand from the Pittsburgh Life Sciences Greenhouse [39] to add two new members to the Consortium. Figure 4 shows the additional funds generated by the members of the PCABC.

In spite of the successes mentioned above, it cannot be emphasized enough that one of the fundamental issues related to building and managing a biorepository is the large financial commitment needed by various stakeholders involved. In addition to the millions of dollars spent by other international biorepository initiatives [27–34], a recent report by the NCI Advisory Bodies estimated over $50 million is spent annually by the NCI on biorepository-related activities [41, 42]. Furthermore, a report by RAND corporation [2] states that accurate determination of the actual costs of collecting, processing, storing, and distributing tissue samples as well its supporting informatics infrastructure and operating costs need to be determined as a “best practice” for cost recovery to financially sustain future biorepositories. This highlights the importance of standardizing and sharing tools by all groups so that unnecessary efforts are not duplicated, as well as, similar groups can build upon the lessons learned by others. As the Consortium prepares to open its biorepository for requests by interested researchers, it is still unclear whether it will be able to become self-funding through fees generated by specimen requests.

### Utilization and marketing of the PCABC biorepository

As reveal above, a large financial commitment is needed for a project like the PCABC to completely be operational and self-sufficient. Leveraging the work done by other collaborative groups [43, 44] that had two member institutions of the PCABC was a key factor for much of the success the Consortium consummated with its limited funding. When compared, each member of the NCI collaborative groups received on average over $1 million/year [45–47] as opposed to each of the initial seven PCABC member institutions receiving approximately $262K/year (PCABC total budget = $5.5mil/3years). The members of the PCA do acknowledge that much work still needs to be addressed in terms of marketing this resource developed for the research community. Much of the utilization of the biospecimens to date has been limited to a handful of pilot studies between the member institutions and numerous requests through partnerships with collaborative tissue groups from the NCI [43, 44]. Furthermore, the Consortium set its priority on several issues that needed to be addressed prior to the onset of utilization of the tissue resources. These issues included (1) intellectual property issues between the users and providers of the resources, (2) IRB issues related to confidential and privacy sharing of tissues and data across multiple institutions, and (3) infrastructure issues dealing with a common bioinformatics platform. As described by other repositories [3], marketing a biospecimen resource like the PCABC to the broader research community will also be a key factor in measuring its success for utilization of the biospecimens. However, with substantial new federal funding from the National Cancer Institute’s cancer Biomedical Informatics Grid (caBIG, see http://cabig.nci.nih.gov) initiatives [21], the members of the PCABC are committed to sustaining and continue to build the infrastructure developed by the Consortium, and are confident that its investment will provide an invaluable resource for large scale cancer biomarker research, that will lead to the demonstration of multiple new biomarkers as clinically important guides to tumor classification, prognosis, response to therapy, and/or clinical course of disease.

## Conclusion

The technological achievements and the statewide informatics infrastructure that have been established by the Consortium will enable robust and efficient studies of biomarkers and their relevance to the natural history and clinical course of cancer. Studies resulting from the creation of the Consortium may allow for better classification of cancer types, more accurate assessment of disease prognosis, a better ability to identify the most appropriate individuals for clinical trial participation, and better surrogate markers of disease progression and/or response to therapy. Of significance, the bioinformatics infrastructure, data warehouse, and biorepository created through Consortium activities will serve as a model as well as catalyst, and would be made available for use by researchers and institutions and other collaborative groups that are presently not part of this research effort.

In summary, the Pennsylvania Cancer Alliances Bio-informatics, Data warehouse and Bio-repository project has created a central resource through which researchers can find highly annotated tissue samples. The resource will have no access at any time to patient identified data and tissue will not be made available to researchers without the approval by IRB and Scientific Review Committee. Furthermore, we believe that the cancer centers of Pennsylvania have the skills and experience necessary to develop, build and implement a statewide network of highly annotated tissue specimens with relevant demographic, clinical, molecular, and outcome data for biomedical research. Based on the successful implementation of this system, the Consortium hopes other groups initiating similar projects will build upon the lessons learned from the PCABC, for some of the most important problems in biomedical as well as clinical informatics, including medical record de-identification, data mining tools and complex data warehousing of biology data. By integrating multimodal (pathological, clinical and molecular) data in the annotated tissue repository, creating query, visualizing techniques for these diverse data types and by making this information available statewide, we can help to provide much better selected and characterized tissues for research. In the process, we hope to foster new understanding of disease evolution and progression that would eventually help make Pennsylvania a leader in cancer research and biomedical technology.

## Supplemental Files

The Master List of purposed Biomarkers (additional file #1)

The Intellectual Property /Technology Transfer Agreements (additional file #2)

Biomarker Disclosure Form (additional file #3)

Template IRB Protocol for PCABC project (additional file #4)

Template IRB Protocol for the Honest Broker system (additional file #5)

Universal Consent Template (additional file #6)

Material Transfer Agreement Template (additional file #7)

The freely available CDEs developed for breast, prostate, and melanoma by the Consortium can be downloaded from its public database link on the PCABC website.

Prostate CDEs (additional files #8)

Breast CDEs (additional files #9)

Melanoma CDEs (additional files #10)

## Figures and Tables

**Table 1.** **Skill set of committee members.** Summary of the required skill sets and roles that make up each of the committees of the PCABC organization.

**Committee**	**Skill sets of committee members**
Executive	Cancer Center Directors and principle investigators of grant
Scientific Review Group	Independent group of experts for each disease/organ category of interest and a liaison member from the working group. Members include biostatisticians, faculty, researchers, and physicians.
Intellectual Property (IP)/Tech Transfer	General counsel, business development directors, technology transfer directors, and faculty members
Disease Organ Site	Leading experts of organ/disease of interest, faculty and researchers, physicians.
Institutional Review Broad (IRB)	Member of a IRB board, HIPAA regulatory specialist, tissue bank director, researchers, physicians, data managers
Common Data Elements (CDE)	Informaticians, molecular pathologists, microbiologists, oncology informatics pathologists, genomics core directors, tissue bankers, cancer registrars, data managers, pathologists, researchers and others who bring a wealth of experience in collaborative data collection.
Working Group	Tissue bankers, cancer registrars, data managers, pathologists, researchers, informaticians, post-doctoral fellows, database administrators and project coordinators.
