# Supplementary material for: A Novel Cross-Disciplinary Multi-Institute Approach to Translational Cancer Research: Lessons Learned from Pennsylvania Cancer Alliance Bioinformatics Consortium (PCABC)
Source: Cancer Inform. 2007 Jun 8;3:255–74. (PMC2675833)
Supplement: The Master List of purposed Biomarkers — (additional file #1) [file cin-03-255-s1.pdf]

Additional File #1

MASTER

Proposed Priority BioMarkers
Targeted Organ Areas
INCLUDES ALL CENTERS
Updated 9/15/03

163 Markers Listed to Date

Pennsylvania Cancer Alliance Bioinformatics Consortium

Print out on legal-size paper

Current Total of priority markers ALL disease sites: 155

Modifications:
2/13/03 – added new markers MSR1 and RNASEL per T. Rebbeck UPenn
2/14/03 – added new markers ATM, ATR and CHK1 per C. Brenner Kimmel/TJU
2/14/03 – added breast as new organ site for FHIT per C. Brenner Kimmel/TJU
4/14/03 – added new markers p300, SRC-1, Ncor, ER-alpha, CC228, CCK1, MEC, CRABP2, NFKBIA, IKB
SCGB3A1, SERPINB5 (MASPIN(p15) – A. Brufsky/P. Hergenroeder UPCI
4/14/03 – added Alpha and Beta forms of the Estrogen Receptor
5/16/03 – added CD4 – new melanoma priority marker per John Kirkwood
7/15/03 – added PDGF Receptor (alpha and beta) – new priority marker per Adam Dicker
7/15/03 – added AKT-2 AND CDKNB/27Kip2 Upenn per Angela DeMichele
9/15/03 – added ESO-1 as priority marker – UPCI John Kirkwood (+assay_
9/15/03 – added EST-Gene Bank accession number BE877801, and EST R33194, EIF3S61P, SNX17, and FL20303 added as priority marker by Adam Brufsky
**** Denotes designated priority marker – see entry for center/disease site**

ProposedMarker	Organ/Disease Sites	Master Grant	Fox Chase	Kimmel	Penn State	Univ Penn	UPCI	Wistar	Rationale	Status of Review
****AKT-2**	Breast					**X**			**Univ Penn/DeMichelle** – Identified as a priority marker 7/15/03	**Added to master list as priority marker 7/15/03**
**ALL1**	Leukemia	X		**X**					****Grant text**:Translocations associated with acute leukemias result in the fusion of one end of the ALL1 gene (the 5' end) with the other end (the 3' end) of 30 or more different genes, leading to the production of chimeric proteins.<br>Acute Leukemia and lymphoblastic leukemia; secondary leukemias caused by anticancer therapy<br><br>**Kimmel**: In adults, abnormalities at region q23 of human chromosome 11 are among the most common alterations observed in acute leukemias. Among young children with acute lymphoblastic leukemia, abnormalities at region 11q23 occur in over 80% of the cases. Abnormalities in this region also are observed in most cases of secondary leukemias, ie leukemias caused by anticancer therapy. The abnormalities at region 11q23 involve translocations with other chromosomes.<br><br>In patients with acute leukemias, analyses of the chromosomal rearrangements led to the identification of the key gene, named *ALL1*, located at chromosomal region 11q23. We have shown that the translocations associated with acute leukemias result in the fusion of one end of the *ALL1* gene (the 5' end) with the other end (the 3' end) of 30 or more different genes, leading to the production of "chimeric" proteins.	
**APC** [Drosophila APC]	Colorectal		**X**						**FCCC**: Defects in APC are a cause of familial adenomatous polyposis (FAP) and Gardners syndrome (GS). Defects contribute to tumor development in patients with noninherited forms of colorectal cancer.	
**AR**	Prostate					**X**			**Univ Penn/Rebbeck:** Associations of inherited genotypes with clinical progression and outcome in men with prostate cancer<br><br>**Univ Penn /Nathanson:** Genotypes associated with recurrence of prostate cancer in collaboration with Duke University (J. Schildkraut). Inherited Genotypes in androgen metabolisn and insulin-like growth factor pathway (SRD5A2, AR, CYP3A4, CYP3A5, IGFBP3, IGF1, VDR)	
**ARF**	Breast<br>Melanoma					**X**			**Univ Penn/Gerrero (Weber)**: Somatic mutations – Genes known to be involved in predisposition and progression of breast and/or melanoma (Somatic mutations PTEN, CDKN2A, ARF, CDK4, TP53, RB1, Hras, Braf, Cyclin D1) (Germline mutations BRCA-1, BRCA-2, PTEN, CDKN2, ARF, CDK4)	**Added 10/09/02**
****ATM**	**Breast**			**X**					**Kimmel/Brenner:** : FHIT is lost in a high proportion of breast tumors, particularly those that	**Added 2/14/03**

**ProposedMarker**	**Organ/Disease Sites**	**Master Grant**	**Fox Chase**	**Kimmel**	**Penn State**	**Univ Penn**	**UPCI**	**Wistar**	**Rationale**	**Status of Review**
									have brca1 and brca2 mutations. There is a growing body of evidence that ATR and CHK1 work with FHIT in an S-phase checkpoint pathway. There has long been evidence that ATM (related to ATR) heterozygosity may predispose to breast cancer and predict sensitivity to radiochemical treatments. (2/14/03)	**Priority Breast Marker for Kimmel**

ProposedMarker	Organ/Disease Sites	Master Grant	Fox Chase	Kimmel	Penn State	Univ Penn	UPCI	Wistar	Rationale	Status of Review
****ATR**	**Breast**			**X**					**Kimmel/Brenner:** : FHIT is lost in a high proportion of breast tumors, particularly those that have brca1 and brca2 mutations. There is a growing body of evidence that ATR and CHK1 work with FHIT in an S-phase checkpoint pathway. There has long been evidence that ATM (related to ATR) heterozygosity may predispose to breast cancer and predict sensitivity to radiochemical treatments. (2/14/03)	**Added 2/14/03 Priority Breast Marker for Kimmel**
**BAX**	Breast					**X**			**Univ Penn/DeMichele**: Somatic markers of tumor progression or prognosis in breast cancer- Somatic tumor markers including p27, p21, cyclin D1, cyclin E, cox-2 enzyme, IL-6 receptor, Her2/neu, EGFR and mutated EGFR (vIII), MCM2, CD34, BCL-2, Bax, Topo II	
**Bcl-2**	Breast<br>Esophageal<br>Head and neck<br>Lung	X		**X**		**X**	**X**		**Grant text**: Abnormal over-expression of Bcl-2 has been identified in cancers associated with tobacco use. Bcl-2 prevents cell death by binding to other proteins in the cell that promote cell death, thereby blocking the entry of cells into this pathway<br>**Kimmel:** The *Bcl-2* gene encodes for a member of a family of proteins that regulates an evolutionarily conserved pathway for programmed cell death. Bcl-2 prevents cell death by binding to other proteins in the cell that promote cell death, thereby blocking the entry of cells into this pathway. Patients whose cancer cells express low levels of Bcl-2 have a higher rate of survival relative to patients with cancer cells that express intermediate or high levels of Bcl-2 protein. High Bcl-2 expression may lead to prolonged survival of cancer cells by allowing these cells to escape programmed cell death.<br><br>Abnormal overexpression of Bcl-2 has been identified in cancers associated with tobacco use, including head and neck, lung, and esophageal cancer. Indeed, abnormal Bcl-2 expression was identified in ~25% of head and neck cancers, ~25% of lung cancers, and ~30% of esophageal cancers. Abnormal Bcl-2 overexpression is significantly correlated with the intensity of smoking in patients with lung cancer and head and neck cancer. Abnormal overexpression of Bcl-2 is considered to be a causative event in tobacco-related tumors. Bcl-2 expression has been suggested as a marker of malignancy in smoking-related tumors.<br><br>**UPCI/Grandis**: Not a well-established marker for head and neck cancer although we found and published evidence of upregulation in about 30% of cases. However, in the absence of mechanistic data (few if any SCCHN cell lines express Bcl-2) it would be hard to know how to analyze marker results. I have no objection to keeping on list.<br><br>**Univ Penn/DeMichele**: Somatic markers of tumor progression or prognosis in breast cancer- Somatic tumor markers including p27, p21, cyclin D1, cyclin E, cox-2 enzyme, IL-6 receptor, Her2/neu, EGFR and mutated EGFR (vIII), MCM2, CD34, **BCL-2**, Bax, Topo II	

ProposedMarker	Organ/Disease Sites	Master Grant	Fox Chase	Kimmel	Penn State	Univ Penn	UPCI	Wistar	Rationale	Status of Review

ProposedMarker	Organ/Disease Sites	Master Grant	Fox Chase	Kimmel	Penn State	Univ Penn	UPCI	Wistar	Rationale	Status of Review
**Beta Tubulin Isotope III**	Lung						**X**		**UPCI/Belani**: The taxanes are novel chemotherapy agents, widely used in the treatment of non-small cell lung cancer. They act by stabilizing the microtubule architecture, thereby inhibiting cell division. Since the taxanes bind to the beta tubulin subunit of the microtubule architecture, abnormalities in the binding sites for the taxanes could lead to decreased efficacy of the taxanes. Over expression of beta tubulin isotype has been noted in lung cancer cells. Decreased survival has been reported for patients with NSCLC that have beta tubulin isotype III overexpression, when treated with a taxane. This could be an important cellular mechanism that contributes to resistance to the anti-cancer effects of the taxanes, by the cancer cells. A clinical trial, which will be underway at the UPCI will assign patients with lung cancer to various chemotherapy regimens based on the genetic profile of the tumors, that includes the beta tubulin isotype III.	
**BFGF/FGFR1**	Melanoma						**X**		**UPCI Kirkwood**: Predisposing & progression-associated genes in melanoma of both sporadic and familial types<br><br>Work published in the past decade from other sites, and from our center indicate that bFGF and its receptor FGFR1 as well as the signaling molecule STAT3 are important in human melanoma progression, and modifiable with antisense approaches. Recent work just published this summer has identified the importance of bRAF as a new predisposing gene for melanoma, and current work with RAF kinase inhibitors has been underway even before this finding, suggesting that there are tools emerging. We ought to focus greater effort upon this area, which is a major focus of both the UPCI and the Wistar/Penn Centers.<br><br>These genes and gene products re relevant to melanoma, and would be applied consistently in this large and growing population of patients under study both in Pittsburgh and in Philadelphia; the bFGF/FGFR1 axis relevant to angiogenesis would have much broader application, but already is being monitored in trials of the national cooperative groups (E2602 phase II trial of PEG-IFN for patients with melanoma and elevated bFGF levels).	
**BLCA-4**	Bladder	X							****Grant text**: Bladder cancer is a smoking related cancer and with the specificity and sensitivity that we have seen so far, would appear to fit in well with what is being proposed for statewide biomarker analysis. UPCI has developed a unique marker derived from nuclear matrix proteins that is specific for bladder cancer called BLCA-4.	
**Braf**	Breast<br>Melanoma					**X**	**X**		**UPCI Kirkwood**: Predisposing & progression-associated genes in melanoma of both sporadic and familial types<br><br>**Univ Penn/DuPont Guerry**: Melanoma susceptibility. Germline variants in XP genes, genes	

ProposedMarker	Organ/Disease Sites	Master Grant	Fox Chase	Kimmel	Penn State	Univ Penn	UPCI	Wistar	Rationale	Status of Review
									regulating COX-2, **B-raf**, CDKN2a. Somatic markers of progression, prognosis, and proliferation, by histology (ki-67, p16 and its methylation state, **B-raf).** Prediction of response to small molecule inhibitors.<br><br>**Univ Penn/Gerrero (Weber)**: Somatic mutations – Genes known to be involved in predisposition and progression of breast and/or melanoma (Somatic mutations PTEN, CDKN2A, ARF, CDK4, TP53, RB1, Hras, Braf, Cyclin D1)	
**BRCA-1**	Breast<br>Melanoma					**X**		**X**	**Univ Penn/Gerrero (Weber)**: Genes known to be involved in predisposition and progression of breast and/or melanoma (Germline mutations BRCA-1, BRCA-2, PTEN, CDKN2, ARF, CDK4)<br><br>**Wistar: Rationale Pending**	
**BRCA-2**	Breast<br>Melanoma					**X**			**Univ Penn/Gerrero (Weber)**: Genes known to be involved in predisposition and progression of breast and/or melanoma (Germline mutations BRCA-1, BRCA-2, PTEN, CDKN2, ARF, CDK4)	
**BRMS1**	Breast<br>Melanoma	X			**X**				****PSU/Hershey**: Based upon high frequency deletions and loss of heterozygosity of 11q13 in advanced human breast cancer, we introduced an intact copy of chromosome 11 into human breast cancer cells and observed suppression of metastasis. Differential display was used to identify BRMS1, which maps to this location and which itself suppresses metastasis in human breast, murine mammary and human melanoma cell lines. BRMS1 appears to suppress metastasis by restoring gap junctional intercellular communication, apparently by up-regulating connexin 43 (Cx43) and down-regulating connexin 32 (Cx32) expression.	**ADD TO TABLE 10/09/02**
**Calnexin**	Melanoma<br>Prostate						**X**		**UPCI/Chatta-Shurin-Dhir-Ferrone:** The above genes are components of the antigen processing machinery, which have been shown to be disrupted in Melanoma (Ferrone et al). In collaboration with Dr Ferrone (RPCI), we are undertaking an investigation of these genes in prostate cancer both at the cDNA, as well as at the protein level.	
**CCL28 (CCK1, MEC chemokine ligand 28)**	Breast						**X**		**UPCI/Brufsky/Hergenroeder**: Mammary Enriched Chemokine expressed in normal mammary epithelial cells and found to be decreased in breast carcinoma. Reference: Porter, Dale A, A Sage View of Breast Tumor Progression. Cancer Research 61:5697-5702, 2001	**Added to table 04/14/03**
****CCND1 (cyclin D1)**	Bladder<br>Breast<br>Colorectal<br>Esophageal<br>Head and neck	**X**				**X**	**X**		**UPCI/Steinman**: Cyclin D1 over expression (especially coupled with low levels of p27) has been shown to be predictive of poor outcome in breast and colorectal cancer. Like p27 it is independently prognostic on multivariable analysis and seems to correlate with increased aggressiveness of tumors. Loss of cyclin D1 is protective in mouse models of induced breast cancers by neu or ras oncogenes. Of note, CCND1 expression can be easily detected by FISH and	**Priority marker Upenn 7/15/03**

ProposedMarker	Organ/Disease Sites	Master Grant	Fox Chase	Kimmel	Penn State	Univ Penn	UPCI	Wistar	Rationale	Status of Review
	Liver<br>Lung<br>Melanoma<br>Oral<br>Thyroid								probes are available from labs in the consortia.<br><br>**UPCI/Gollin**:<br>Cyclin D1/*CCND1*: Band 11q13, which harbors the locus for the cyclin D1 gene (*CCND1*), is amplified frequently in SCCHN (~30-50% of tumors), and to a lesser degree in a number of other carcinomas, including other aerodigestive cancers, breast, liver, and bladder cancer [1-5]. This amplification is best illustrated by FISH and protein overexpression is demonstrated by immunohistochemistry or Western blotting. Several genes have been shown to be amplified in this region, including *CCND1* which codes for cyclin D1, *EMS1* which encodes human cortactin, an actin binding protein possibly involved in the organization of the cytoskeleton and cell adhesion structures, and *FGF3* and *FGF4* (fibroblast growth factors 3 and 4, also called *INT2* and *HSTF1*), but the latter two genes are not overexpressed [6]. Of the amplified and overexpressed genes, *CCND1* is thought to play a direct role in SCCHN, since both its RNA transcript and protein are overexpressed [7]. Cyclin D1 is a critical cell cycle regulatory protein that drives the cell cycle from the G1 to the S phase of the cell cycle. Cyclin D1 binds a cyclin-dependent kinase, CDK4 or CDK6, and phosphorylates and inactivates the retinoblastoma tumor suppressor protein pRB, resulting in release of the bound E2F transcription factors and cell cycle progression. Overexpression of cyclin D1 may lead to reduction in the requirement for growth factors or mitogens, shortening of the G1 phase of the cell cycle and thus, premature passage through the G1-S cell cycle transition, resulting in propagation of unrepaired DNA damage, accumulation of genetic alterations, and a growth advantage for the cells. In SCCHN, cyclin D1 protein overexpression correlates with shorter time to recurrence, and with higher stage disease, lymph node involvement, and reduced overall survival [8-11]. *CCND1* gene amplification has been correlated with increased mRNA expression and laryngeal tumor progression [8]. Overexpression of cyclin D1 protein has been reported to be an independent prognostic factor in SCCHN [2,10]. In one study of hypopharyngeal SCCHN, cyclin D1 gene amplification and protein overexpression correlated with prognosis and also indicated that cyclin D1-negative tumors responded well to multimodality treatment [12]. Cyclin D1 overexpression also appears to alter sensitivity of tumor cells to ionizing radiation [13,14]. Two recent studies reported that antisense cyclin D1 inhibited cell proliferation, induced apoptosis, and led to SCCHN tumor shrinkage [15,16]. Recently, Boyle et al. (1999) demonstrated that the chemopreventive antiproliferative effect of retinoids on bronchial epithelial cells is directly linked to the degradation of cyclin D1 via the 26S proteosome degradation pathway [17], although additional involvement of the IGF-1 pathway has not been ruled out [18]. Papadimitrakopoulou and Hong (2000)]) report that a combination of altered cyclin D1 expression and p16 loss is predictive of adverse outcome in SCCHN patients in chemoprevention trials [19]. Overexpression of cyclin D1 protein is associated with aberrant cell cycle regulatory function. Therefore, quantitative immunohistochemistry will provide key functional information beyond that revealed by copy number alterations alone.Thus, for these reasons, we have chosen to examine the cyclin	

ProposedMarker	Organ/Disease Sites	Master Grant	Fox Chase	Kimmel	Penn State	Univ Penn	UPCI	Wistar	Rationale	Status of Review
									D1/*CCND1* gene and protein as biomarkers in oral and head and neck cancers in this study. Of note, *CCND1* copy number can be easily detected by FISH and probes are available from labs in the consortium. Cyclin D1 protein expression can be detected by quantitative immunohistochemistry in laboratories in the consortium.<br><br>High frequency in oral and head and neck cancer, with about 45% tumor showing CCND1 amplification, making this an ideal target for further validation. However, breast , lung, esophageal, bladder and liver cancers seem appropriate targets as well<br><br>**UPCI/Grandis**: Cumulative evidence supports amplification and over-expression I SCCHN. It belongs on the list.<br><br>**Univ Penn/DeMichele**: Somatic markers of tumor progression or prognosis - Somatic tumor markers including p27, p21, **cyclin D1**, cyclin E, cox-2 enzyme, IL-6 receptor, Her2/neu, EGFR and mutated EGFR (vIII), MCM2, CD34, BCL-2, Bax, Topo II<br><br>**Univ Penn/Gerrero (Weber)**: Somatic mutations – Genes known to be involved in predisposition and progression of breast and/or melanoma (Somatic mutations PTEN, CDKN2A, ARF, CDK4, TP53, RB1, Hras, Braf, Cyclin D1)	

ProposedMarker	Organ/Disease Sites	Master Grant	Fox Chase	Kimmel	Penn State	Univ Penn	UPCI	Wistar	Rationale	Status of Review
****CD4**	Melanoma						**X**		**UPI/Kirkwood – Added 05/016/03 by Dr. Kirkwood.**<br><br>**UPCI/Kirkwood:** Designated as a priority marker 9/15/03. Will perform immunohistological/in situ assays . Paired embedded frozen samples of primary, nodal or nodal, metastatic disease tissue. Will collect density and distribution of host response elements	**ADDED to table 05/16/03**<br><br>**Designated priority marker to measure by UPCI/Kirkwood 9/15/03**
**CD7**	CTCL							**X**	**Wistar:** Frequently lost in CTCL (Cutaneous T-Cell lymphoma)	
****CD8**	Melanoma					**X**	**X**		**Univ Penn/DuPont Guerry**: Somatic markers of immunogenicity (TIA-1, CD8, CD4)<br><br>**UPCI/Kirkwood:** Designated as a priority marker 9/15/03. Will perform immunohistological/in situ assays . Paired embedded frozen samples of primary, nodal or nodal, metastatic disease tissue. Will collect density and distribution of host response elements	**ADDED 10/09/02**<br><br>**Priority melanoma marker Upenn 7/15/03**<br><br>**Designated priority marker to measure by UPCI/Kirkwood 9/15/03**
**CD26**	CTCL							**X**	**Wistar:** Frequently lost in CTCL	
**CD34**	Breast					**X**			**Univ Penn/DeMichele**: Somatic markers of tumor progression or prognosis - Somatic tumor markers including p27, p21, cyclin D1, cyclin E, cox-2 enzyme, IL-6 receptor, Her2/neu, EGFR and mutated EGFR (vIII), MCM2, CD34, BCL-2, Bax, Topo II	
**CD83**	Breast<br>Prostate						**X**		**UPCI/Chatta-Shurin:** CD83 is expressed at a high level on immune-competent, activated and mature dendritic cells, which are considered to be the most potent antigen-presenting cells. Infiltration of the tumor mass by dendritic cells was associated with late tumor recurrence and better patient survival for many different types of cancer. We have demonstrated that patients have a longer PSA relapse free survival time after radical prostatectomy if the prostate carcinoma tissues expressed higher number of live CD83+ dendritic cells. These and other data provide in vivo support for the concept that CD83+ dendritic cells provide signals for direct intralesional T cell activation.	

ProposedMarker	Organ/Disease Sites	Master Grant	Fox Chase	Kimmel	Penn State	Univ Penn	UPCI	Wistar	Rationale	Status of Review
**CD9**	Melanoma					**X**			**Univ Penn/DuPont Guerry**: Somatic markers of invasion and adhesion (MMP-2, MUC-18, alpha V/beta 3, CD9)	**ADDED 10/09/02**
**Cdc25A**	Thought to be over-expressed in many human tumors. NEED TO CLARIFY ORGAN/DISEASE SITES	**X**							****Grant text**: The Cdc25 phosphatases hydrolyze both phosphoserine/threonine as well as phosphotyrosine residues on the same protein substrate and control cell cycle progression by activating cyclin-dependent kinases (Cdk). Three Cdc25 homologs exist in humans: Cdc25A, Cdc25B and Cdc25C. Two splice variants of Cdc25A have been reported, while Cdc25B and C have at least 7 and 5 each. The functional significance of these variants is currently unknown. Cdc25A and B are oncogenic and are thought to be over-expressed in many human tumors.	
**Cdc25B**	Thought to be over-expressed in many human tumors. NEED TO CLARIFY ORGAN/DISEASE SITES	**X**							****Grant text**: The Cdc25 phosphatases hydrolyze both phosphoserine/threonine as well as phosphotyrosine residues on the same protein substrate and control cell cycle progression by activating cyclin-dependent kinases (Cdk). Three Cdc25 homologs exist in humans: Cdc25A, Cdc25B and Cdc25C. Two splice variants of Cdc25A have been reported, while Cdc25B and C have at least 7 and 5 each. The functional significance of these variants is currently unknown. Cdc25A and B are oncogenic and are thought to be over-expressed in many human tumors.	
**CDID**	Leukemia							**X**	**Wistar**: CDID, along with TNFSF10, TRAIL and DUSP1 are all over-expressed in CTCL T-cells	
**CDK4**	Melanoma<br><br>Breast<br>Melanoma					 <br>**X**		**X**	**Wistar** – Two candidate genes have been identified in which germline mutations co-segregate with familial melanoma, CDKN2A and CDK4. CDKN2A codes for p16, which binds to CDK4 and CDK6, and inhibits their catalytic activity and cyclinD. Deletions or mutations in CDKN2A may affect the relative balance of functional p16 and cyclin D, resulting in abnormal cell growth.<br><br>**Univ Penn/Gerrero (Weber)**: Somatic mutations – Genes known to be involved in predisposition and progression of breast and/or melanoma (Somatic mutations PTEN, CDKN2A, ARF, CDK4, TP53, RB1, Hras, Braf, Cyclin D1) (Germline mutations BRCA-1, BRCA-2, PTEN, CDKN2, ARF, CDK4)	
****CDKN1A/p21**	Breast<br>Melanoma							**X**	**Wistar:** In early stage melanoma cells, IL-6 induced growth inhibition involves induction of p21 WAF1/CIP1 which is lost in the course of tumor progression as a result of a dominant oncogenic event. Markers of the risk of CDIS patients for invasive breast cancer and molecular targets of chemoprevention in breast intraepithelial neoplasia. (Synonyms Waf1/Cip1)	**Priority breast marker per Kimmel Juan Palazzo**

ProposedMarker	Organ/Disease Sites	Master Grant	Fox Chase	Kimmel	Penn State	Univ Penn	UPCI	Wistar	Rationale	Status of Review
	Breast					X			**Univ Penn/DeMichele**: Somatic markers of tumor progression or prognosis - Somatic tumor markers including p27, **p21**, cyclin D1, cyclin E, cox-2 enzyme, IL-6 receptor, Her2/neu, EGFR and mutated EGFR (vIII), MCM2, CD34, BCL-2, Bax, Topo II	
**CDKN1B (p27kip1)**	Breast<br>Melanoma<br>Prostate	X					X		****Grant text - UPCI/ Steinman:** (CDKN1B (p27kip1) on chromosome 12p13 is a cyclin dependent kinase inhibitor, which modulates cell cycle arrest in the setting of contact inhibition and nutrient deprivation. In addition it has both pro-and anti-apoptotic effects and has been implicated in the regulation of cell motility. In mouse models, p27 has been shown to be haplo-insufficient as a tumor suppressor. Low levels of p21 have been shown to be predictive of poor outcome and decreased progression-free survival in over twenty solid and liquid malignancies. Loss of p27 probably occurs early in invasion and not at initiation. However, p27 regulation is both at the transcriptional and posttranscriptional level and so gene arrays without proteomics would not be optimally informative with this marker. Affects cancer in every organ.	
	Breast					X			**Univ Penn/DeMichele**: Somatic markers of tumor progression or prognosis - Somatic tumor markers including p27, p21, cyclin D1, cyclin E, cox-2 enzyme, IL-6 receptor, Her2/neu, EGFR and mutated EGFR (vIII), MCM2, CD34, BCL-2, Bax, Topo II	
**CDKN2A/p16** kinase inhibitor gene	Breast<br>Head and neck<br>Melanoma<br>Oral<br>Prostate	X	X			X	X	X	****Grant text: UPCI/Gollin**: CDKN2A can be easily detected by FISH and probes are available from labs in the consortia. CDKN2A is one of the most common and earliest tumor suppressor loci lost in human tumors<br><br>**FCCC** – Rationale pending<br><br>**UPCI/Kirkwood**: Predisposing genes for familial melanoma.<br><br>**Univ Penn/DuPont Guerry**: Melanoma susceptibility. Germline variants in XP genes, genes regulating COX-2, B-raf, **CDKN2a**. Somatic markers of progression, prognosis, and proliferation, by histology (ki-67, **p16** and its methylation state, B-raf<br><br>**Univ Penn/Gerrero (Weber)**: Somatic mutations – Genes known to be involved in predisposition and progression of breast and/or melanoma (Somatic mutations PTEN, CDKN2A, ARF, CDK4, TP53, RB1, Hras, Braf, Cyclin D1) - (Germline mutations BRCA-1, BRCA-2, PTEN, CDKN2, ARF, CDK4)<br><br>**Wistar** – Two candidate genes have been identified in which germline mutations co-segregate with familial melanoma, CDKN2A and CDK4. CDKN2A codes for p16 which binds to CDK4	

ProposedMarker	Organ/Disease Sites	Master Grant	Fox Chase	Kimmel	Penn State	Univ Penn	UPCI	Wistar	Rationale	Status of Review
									and CDK6 and inhibits their catalytic activity and cyclinD. Deletions or mutations in CDKN2A may affect the relative balance of functional p16 and cyclin D, resulting in abnormal cell growth<br><br>**UPCI/Brufsky Hergenroeder** – Noted as priority marker (Reference: Yaswen, Paul Epigenetic changes accompanying human mammary epithelial cell immortalization. Jrnl of Mammary Gland Biology and Neoplasia 6 (2): 223-234, 2001	
****CDKNB (27kip2)**	**Breast**					**X**			**Upenn/DeMichele** – Identified as priority marker 7/15/03	**Added 7/15/03**
****CHK1**	**Breast**			**X**					**Kimmel/Brenner:** : FHIT is lost in a high proportion of breast tumors, particularly those that have brca1 and brca2 mutations. There is a growing body of evidence that ATR and CHK1 work with FHIT in an S-phase checkpoint pathway. There has long been evidence that ATM (related to ATR) heterozygosity may predispose to breast cancer and predict sensitivity to radiochemical treatments. (2/14/03)	**Added 2/14/03 Priority Breast Marker for Kimmel**
**C-Kit**	Leukemia<br>Lung<br>Ovarian<br>Gastrointestinal		**X**				**X**		**UPCI/Belani**: Activation of the KIT tyrosine kinase by somatic mutation has been documented in a number of human malignancies, including gastrointestinal stromal tumor (GIST), seminoma, acute myelogenous leukemia (AML), and mastocytosis. In addition, paracrine or autocrine activation of this kinase has been postulated in numerous other malignancies, including small-cell lung cancer and ovarian cancer. At least 70% of small cell lung cancers express the Kit receptor tyrosine kinase and its ligand, stem cell factor (SCF). Numerous lines of evidence have demonstrated that this coexpression constitutes a functional autocrine loop, suggesting that inhibitors of Kit tyrosine kinase activity could have therapeutic efficacy in this disease. There is an ongoing clinical trial at the UPCI that is evaluating the efficacy of combining Imatinib, an inhibitor of the c-kit tyrosine kinase, with chemotherapy for patients with advanced small cell lung cancer.<br><br>**FCCC/Eisenberg**: Rationale pending	
**c-Met (see also HGF)**	Lung cancer<br>Melanoma						**X**	**X**	**UPCI/Siegfried:** We have a data set of 82 lung cancer cases for which we have pathologic staging including lymph node dissection, treatment information, and outcome data. We have shown a significant association between level of HGF in the tumor (from frozen tumor tissue) and poor outcome (either local recurrence or metastatic disease). The effects are most pronounced in early-stage disease.	

ProposedMarker	Organ/Disease Sites	Master Grant	Fox Chase	Kimmel	Penn State	Univ Penn	UPCI	Wistar	Rationale	Status of Review
									We are currently measuring c-Met level as well. HGF is an independent prognostic indicator and HGF level is independent of stage of disease.<br><br>HGF may be valuable to predict poor outcome in lung cancer patients with Stage IA and IB disease, and could be used as an indication for adjuvant therapy in patients who otherwise would get no treatment.<br><br>It would be extremely valuable to get additional measurements from tumors at other institutions to verify this and to increase the sample size.<br><br>**Wistar**: Overproduced and activated in many malignant tumors. The interaction of HGF and c-Met activates cellular signals that stimulate cancer cells to spread into neighboring normal tissues and metastasize to distant tissues.	

ProposedMarker	Organ/Disease Sites	Master Grant	Fox Chase	Kimmel	Penn State	Univ Penn	UPCI	Wistar	Rationale	Status of Review
**CMRF44**	Breast<br>Prostate						X		**UPCI-Chatta-Shurin:** CMRF-44 and 56 are dendritic cell early activation/differentiation antigens with limited expression on other hematopoietic cell populations. CMRF-44 is expressed on the surface of cultured human blood dendritic cell, which subsequently acquire CD83 expression upon activation. CMRF-44 is also induced on isolated human Langerhans cells and dermal dendritic cells. A growing body of evidences suggests that both dendritic cell number and dendritic cell activation appear substantially deficient in human breast cancers and prostate cancer. Evaluation of the numbers of activated and suppressed dendritic cells within the tumor mass may serve as a good marker of tumor progression and the activity of the antitumor immune response. It might also predict the efficacy of different therapeutic approaches including immunotherapies.	
**CMRF56**	Breast<br>Prostate						X		**UPCI-Chatta-Shurin:** CMRF-44 and 56 are dendritic cell early activation/differentiation antigens with limited expression on other hematopoietic cell populations. CMRF-44 is expressed on the surface of cultured human blood dendritic cell, which subsequently acquire CD83 expression upon activation. CMRF-44 is also induced on isolated human Langerhans cells and dermal dendritic cells. A growing body of evidences suggests that both dendritic cell number and dendritic cell activation appear substantially deficient in human breast cancers and prostate cancer. Evaluation of the numbers of activated and suppressed dendritic cells within the tumor mass may serve as a good marker of tumor progression and the activity of the antitumor immune response. It might also predict the efficacy of different therapeutic approaches including immunotherapies.	
**Collagen I alpha**	Breast					X			**Univ Penn/DeMichele**: Predictors of response or late effects in breast cancers (Including : IGF-1, ER polymorphisms - PvuII, Xbal, Codon 325 C>G, TA repeat, Vitamin D receptor (VDR) polymorphisms - Taq1, Fokl start codon **Collagen I alpha (COLIA1**) polys - MSC1, Sp1G>T, IL-6 polys - CA repeat, Nt-634 C>G, -174 G>C TGF-beta polys -509C>T, -869C>T)	
**COMT**	Breast					X			**Univ Penn/Weber/Rebbeck**: **Breast** cancer – Candidate low penetrance susceptibility alleles Germline variants genes involved in hormone metabolism, DNA damage response and immune surveillance (CYP3A4, CYP3A5, CYP17, COMT, HSD3B2, CYP19, IL1, IL1R, TNFa,, IL10, IL6, IL12, XPD,XRCC2, XRCC3)	
**CRABP2**	Breast						X		**UPCI/Brufsky/Hergenroeder**: Retionoic Acid Binding Protein 2 – positive regulator of retinoic acid signaling in breast cancer (Reference: Eutimova, Vesna: Identification of Breast Cancer Metastisis-associated Genes by Chip Technology, Anti-cancer Res 21: 3799-3806, 2001 (citation 44)	**Added to table 04/14/03**
**CRADD**	CNS tumors						X		**UPCI/Kokkinakis -** Some of the major changes in gene expression in CNS tumors under	

ProposedMarker	Organ/Disease Sites	Master Grant	Fox Chase	Kimmel	Penn State	Univ Penn	UPCI	Wistar	Rationale	Status of Review
									methionine stress that have attracted immediate attention include the upregulation of Mda-7 , Cradd and BAK. The death signal, accepted through cell's surface receptors by specific ligand binding, induces oligomerization of these receptors, which is essential for the apoptotic response. Oligomerization of cells' receptors is followed by protein-protein interactions mediated through receptor's cytoplasmic domain termed as death domain (DD) which is involved in transducing proliferative and/or apoptotic signals by interacting with cytoplasmic proteins containing similar DD motifs (TRADD, TRAF, RIP, FADD/MORT1, RAID, CRADD, caspase 8/FLICE/MACH/Mch5 and caspase 10/Flice2/Mch4).	
**Cyclin B1**	Prostate						X		**UPCI/Finn-Chatta-Dhir:** Both Cyclin B1 and MUC-1 are over expressed in many tumors, and maybe potential targets for immunotherapy (Finn et al). We are undertaking an investigation of these genes in prostate cancer both at the cDNA, as well as at the protein level.	
****Cyclin E**	Breast					X			**Univ Penn/DeMichele**: Somatic markers of tumor progression or prognosis - Somatic tumor markers including p27, p21, cyclin D1, **cyclin E**, cox-2 enzyme, IL-6 receptor, Her2/neu, EGFR and mutated EGFR (vIII), MCM2, CD34, BCL-2, Bax, Topo II	**Priority marker 7/13/03 UPenn**
****Cyclooxygenase-2 (COX-2)**	Breast<br><br>Head and neck<br>Lung<br><br>Melanoma<br><br>Prostate			 <br><br><br><br><br>X		X<br><br><br><br><br>X	 <br>X		**Univ Penn/DeMichele**: Somatic markers of tumor progression or prognosis - Somatic tumor markers including p27, p21, cyclin D1, cyclin E, cox-2 enzyme, IL-6 receptor, Her2/neu, EGFR and mutated EGFR (vIII), MCM2, CD34, BCL-2, Bax, Topo II<br><br>**UPCI/Shin**: Recent literature suggests that COX-2 appears to be an important marker for head and neck cancer and lung cancer. There is a growing evidence that COX-2 shows a prognostic indicator for tumors of upper aerodigective tract including head, neck and lung.<br><br>**Univ Penn/DuPont Guerry**: Melanoma susceptibility. Germline variants in XP genes, genes regulating COX-2, B-raf, CDKN2a<br><br>**Kimmel/TJU/Dicker** – Identified as priority prostate marker by Adam Dicker 7/15/03	**Priority prostate marker per Adam Dicker 7/15/03**<br><br>**Priority breast marker per Angela DeMichele 7/15/03**
**c-YES**	Melanoma							X	**Wistar**: May play a role in the malignant progression of human melanocyte towards the brain metastatic phenotype.	
**CYP17, A T to C transition (A2 allele) in the 5' promoter**	Breast<br>Prostate					X		X	**Univ Penn/Weber/Rebbeck**: **Breast** cancer – Candidate low penetrance susceptibility alleles Germline variants genes involved in hormone metabolism, DNA damage response and immune surveillance (CYP3A4, CYP3A5, CYP17, COMT, HSD3B2, CYP19, IL1, IL1R, TNFa,, IL10, IL6, IL12, XPD,XRCC2, XRCC3)	

ProposedMarker	Organ/Disease Sites	Master Grant	Fox Chase	Kimmel	Penn State	Univ Penn	UPCI	Wistar	Rationale	Status of Review
**region**									**Wistar**: Rationale Pending	
**CYP19**	Breast					X			**Univ Penn/Weber/Rebbeck**: **Breast** cancer – Candidate low penetrance susceptibility alleles Germline variants genes involved in hormone metabolism, DNA damage response and immune surveillance (CYP3A4, CYP3A5, CYP17, COMT, HSD3B2, CYP19, IL1, IL1R, TNFa,, IL10, IL6, IL12, XPD,XRCC2, XRCC3)	
**CYP2B6**	Breast					X			**Univ Penn/Rebbeck/DeMichele/Aplene**: Associations of inherited genotypes with chemosensitivity/differential toxicity, clinical progression and outcome following chemotherapy in women with breast cancer (considering "traditional" biomarkers as well, such as ER/PR, Her2/neu, etc.)<br><br>Inherited genotypes in chemotherapy metabolism genes (e.g., CYP3A4, CYP3A5*3, CYP3A5*6, GSTT1, GSTM1, GSTP1 (2 SNPs), NQ01 **CYP2B6**, CYP2C8)	
**CYP2C8**	Breast					X			**Univ Penn/Rebbeck/DeMichele/Aplene**: Associations of inherited genotypes with chemosensitivity/differential toxicity, clinical progression and outcome following chemotherapy in women with breast cancer (considering "traditional" biomarkers as well, such as ER/PR, Her2/neu, etc.)<br><br>Inherited genotypes in chemotherapy metabolism genes (e.g., CYP3A4, CYP3A5*3, CYP3A5*6, GSTT1, GSTM1, GSTP1 (2 SNPs), NQ01 CYP2B6, **CYP2C8**)	
****CYP3A4**	Breast<br>Prostate					X			**Univ Penn/Weber/Rebbeck**: **Breast** cancer – Candidate low penetrance susceptibility alleles Germline variants genes involved in hormone metabolism, DNA damage response and immune surveillance (CYP3A4, CYP3A5, CYP17, COMT, HSD3B2, CYP19, IL1, IL1R, TNFa,, IL10, IL6, IL12, XPD,XRCC2, XRCC3)<br><br>**Univ Penn/Rebbeck**: Associations of inherited genotypes with clinical progression and outcome in men with **prostate** cancer. Inherited genotypes in hormone, cytokine, and DNA damage/repair pathways (e.g., SRD5A2, CYP3A4, CYP3A5, AR, HPC2, IL6, SOD2;<br><br>**Univ Penn/Rebbeck/DeMichele/Aplene**: Associations of inherited genotypes with chemosensitivity/differential toxicity, clinical progression and outcome following chemotherapy in women with **breast** cancer (considering "traditional" biomarkers as well, such as ER/PR, Her2/neu, etc.). Inherited genotypes in chemotherapy metabolism genes (e.g., **CYP3A4**, CYP3A5*3, CYP3A5*6, GSTT1, GSTM1, GSTP1 (2 SNPs), NQ01 CYP2B6, CYP2C8)<br><br>**Univ Penn/Nathanson**: Genotypes associated with recurrence of **prostate** cancer in collaboration with Duke Univ. (J. Schildkraut) Inherited Genotypes in androgen metabolism and	**Priority breast marker per Angela DeMichele 7/15/03**

ProposedMarker	Organ/Disease Sites	Master Grant	Fox Chase	Kimmel	Penn State	Univ Penn	UPCI	Wistar	Rationale	Status of Review
									insulin-like growth factor pathway (SRD5A2, AR, CYP3A4, CYP3A5, IGFBP3, IGF1, VDR)	
****CYP3A5**	Breast<br>Prostate					**X**<br><br>X	**Univ Penn/Rebbeck**: Associations of inherited genotypes with clinical progression and outcome in men with **prostate** cancer. Inherited genotypes in hormone, cytokine, and DNA damage/repair pathways (e.g., SRD5A2, CYP3A4, **CYP3A5**, AR, HPC2, IL6, SOD2<br><br>**Univ Penn/Nathanson**: Genotypes associated with recurrence of **prostate** cancer in collaboration with Duke Univ. (J. Schildkraut) Inherited Genotypes in androgen metabolisn and insulin-like growth factor pathway (SRD5A2, AR, CYP3A4, CYP3A5, IGFBP3, IGF1, VDR)<br><br>**Univ Penn/Weber/Rebbeck**: **Breast** cancer – Candidate low penetrance susceptibility alleles Germline variants genes involved in hormone metabolism, DNA damage response and immune surveillance (CYP3A4, CYP3A5, CYP17, COMT, HSD3B2, CYP19, IL1, IL1R, TNFa,, IL10, IL6, IL12, XPD,XRCC2, XRCC3)	**Priority prostate marker per Upenn 7/15/03**
****CYP3A5*3**	Breast					**X**			**Univ Penn/Rebbeck/DeMichele/Aplene**: Associations of inherited genotypes with chemosensitivity/differential toxicity, clinical progression and outcome following chemotherapy in women with breast cancer (considering "traditional" biomarkers as well, such as ER/PR, Her2/neu, etc.) CYP3A5*3 and CYP3A5*6 are the variant alleles of CYP3A5.<br><br>Inherited genotypes in chemotherapy metabolism genes (e.g., CYP3A4, **CYP3A5*3**, CYP3A5*6, GSTT1, GSTM1, GSTP1 (2 SNPs), NQ01 CYP2B6, CYP2C8)	**Priority breast marker per Angela DeMichele 7/15/03**
****CYP3A5*6**	Breast					**X**			**Univ Penn/Rebbeck/DeMichele/Aplene**: Associations of inherited genotypes with chemosensitivity/differential toxicity, clinical progression and outcome following chemotherapy in women with breast cancer (considering "traditional" biomarkers as well, such as ER/PR, Her2/neu, etc.). CYP3A5*3 and CYP3A5*6 are the variant alleles of CYP3A5.<br><br>Inherited genotypes in chemotherapy metabolism genes (e.g., CYP3A4, CYP3A5*3, **CYP3A5*6**, GSTT1, GSTM1, GSTP1 (2 SNPs), NQ01 CYP2B6, CYP2C8)	**Priority breast marker per Angela DeMichele 7/15/03**
**DD3 RNA**	Prostate	X			**X**				****PSU/Hershey /Clawson** DD3 RNA is highly specific for prostate cancer. It appears to function as a structural, noncoding RNA, which is expressed at high levels, making it an ideal candidate for a molecular detection marker for circulating prostate cancer cells	

ProposedMarker	Organ/Disease Sites	Master Grant	Fox Chase	Kimmel	Penn State	Univ Penn	UPCI	Wistar	Rationale	Status of Review
**DUSP1**	Leukemia							X	**Wistar:** DUSP1 along with TNFSF10, TRAIL and CDID are all over expressed in CTCL T-cells	
**E-Cadherin**	Melanoma							X	**Wistar:** During melanoma development, loss of E-cadherins expression is observed. It is accompanied by a parallel gain of N-cadherin expression that facilitates migration of melanoma cells from epidermis.	
**EGF**	Melanoma						X		**UPCI-Gorelik:** The proposed analysis of the angiogenic factors in the blood of cancer patients is based on our hypothesis of tumor-induced cytokine chaos.<br><br>The Luminex core facility at UPCI provides a possibility to analyze simultaneously 10 or more molecules in the plasma of cancer patients. Therefore the list of angiogenic molecules could be extended to include IL-8, EGF, TNF and IL-6, as well as VEGF, PDGF and FGF.	

ProposedMarker	Organ/Disease Sites	Master Grant	Fox Chase	Kimmel	Penn State	Univ Penn	UPCI	Wistar	Rationale	Status of Review
****EGFR**	Head and neck<br>Lung<br><br><br><br><br><br><br><br><br><br><br><br><br>Breast					X	X		**UPCI/Belani**: EGFR is a transmembrane receptor that activates a cascade of events that lead to cellular proliferation, metastasis, angiogenesis and inhibition of apoptosis, upon ligand binding. EGFR overexpression has been documented in > 65% of patients with NSCLC. Several drugs that inhibit the EGFR pathway are currently under evaluation in the treatment of cancer. Gefitinib, an orally administered inhibitor of the EGFR tyrosine kinase has documented antitumor efficacy in patients with advanced NSCLC.<br><br>**UPCI/Grandis**: Established biomarker in SCCHN that has been shown to correlate with survival, response to therapy and is now a therapeutic target. Please keep on list<br><br>**UPCI/Siegfried:** Expression of EGFR by lung tumors is a negative prognostic factor. This may be useful to combine with other prognostic markers described above. Also, overexpression of EGFR may lead to enhanced tumor progression and could have an impact on response to therapy.<br><br>**Univ Penn/DeMichele**: Somatic markers of tumor progression or prognosis - Somatic tumor markers including p27, p21, cyclin D1, cyclin E, cox-2 enzyme, IL-6 receptor, Her2/neu, EGFR and mutated EGFR (vIII), MCM2, CD34, BCL-2, Bax, Topo II	**Priority breast marker per Angela DeMichele 7/15/03**
****EGFR (vIII)**	Breast					X			**Univ Penn/DeMichele**: Somatic markers of tumor progression or prognosis in breast cancer-Somatic tumor markers including p27, p21, cyclin D1, cyclin E, cox-2 enzyme, IL-6 receptor, Her2/neu, EGFR and **mutated EGFR (vIII)**, MCM2, CD34, BCL-2, Bax, Top<br><br>Mutant of EGFR (deletion of amino acids 6-273).	**ADDED 10/09/02**<br><br>**Priority breast marker per Angela DeMichele 7/15/03**
****EIF3S61P**	Breast						X		**UPCI/Brufsky –** Designated as a priority marker 9/15/03 – assays will be conducted and gene expression results available	**Added 9/15/03 Priority marker by Adam Brufsky – Assays will be performed**
****EST-Gene Bank Accession Number BE877801**	Breast						X		**UPCI/Brufsky –** Designated as a priority marker 9/15/03 – assays will be conducted and gene expression results available<br><br>**Assay**: RT-PCR 12/03 spec req 1ug<br><br>NCI Ref Seq NM_014748 - gene encodes a member of sorting nexin family. Members of this	**Added 9/15/03 Priority marker by Adam Brufsky – Assays will be performed**

ProposedMarker	Organ/Disease Sites	Master Grant	Fox Chase	Kimmel	Penn State	Univ Penn	UPCI	Wistar	Rationale	Status of Review
									family conatin a phox(PX) domain, which is a phospoinositide binding domain and are involved in intracellular trafficking. SYN:KIAA0064, ref sequence from Burkitt's Lymphoma specimen - upreg in IDC with nodal spread	

ProposedMarker	Organ/Disease Sites	Master Grant	Fox Chase	Kimmel	Penn State	Univ Penn	UPCI	Wistar	Rationale	Status of Review
****EST-Gene Bank Accession Number R33194**	Breast						**X**		**UPCI/Brufsky –** Designated as a priority marker 9/15/03 – assays will be conducted and gene expression results available	**Added 9/15/03 Priority marker by Adam Brufsky – Assays will be performed**
****ER**	Breast					**X**			**Univ Penn/Rebbeck/DeMichele/Aplene**: Associations of inherited genotypes with chemosensitivity/differential toxicity, clinical progression and outcome following chemotherapy in women with breast cancer (considering "traditional" biomarkers as well, such as **ER/PR**, Her2/neu, etc.) Inherited genotypes in chemotherapy metabolism genes (e.g., CYP3A4, **CYP3A5*3**, **CYP3A5*6**, GSTT1, GSTM1, GSTP1 (2 SNPs), NQ01 CYP2B6, CYP2C8)	**Priority breast marker per Kimmel Juan Palazzo 2/21/03**
**ER-alpha**	Breast						**X**		**UPCI/Brufsky/Hergenroeder: Priority marker – rational to follow**	**Added to table 04/14/03**
**ERCC1**	Lung						**X**		**UPCI/Belani**: Overexpression of the excision repair cross-complementing 1 (ERCC1) gene, which is crucial in the repair of cisplatin (CDDP)-DNA adducts, is reported to negatively influence the effectiveness of CDDP-based therapy for gastric, ovarian and lung cancers. Recent evidence indicates that Gemcitabine (Gem) may modulate ERCC1 nucleotide excision repair activity, and down-regulation of DNA repair activity by ERCC1 antisense RNA reportedly inhibits synergism of CDDP/Gem. Prospective evaluation of response to platinum therapy and ERCC1 expression is ongoing.	
****ESO-1**<br>NEW 9/15/03	Melanoma						X		**UPCI/Kirkwood:** Designated as a priority marker 9/15/03. Will perform immunohistological/in situ assays . Paired embedded frozen samples of primary, nodal or nodal, metastatic disease tissue. Will collect density and distribution of host response elements	**Designated priority marker to measure by UPCI/Kirkwood 9/15/03**
**EXT1**<br>[C. elegans rib-2)	REMOVE FROM LIST		**X**						**REMOVE - FCCC:**	
**EXT2**	REMOVE FROM LIST		**X**						**REMOVE - FCCC:**	
**FEZ1**	Bladder<br>Breast<br>Hematologic<br>Prostate<br>Urinary	**X**		**X**					**Grant text**: Tumor suppressor gene. Loss of FEZ1 function has been suggested to play a role in the development of prostate, breast and other cancers and in the progression of bladder tumors<br><br>**Kimmel**: Abnormalities at region p21-22 of chromosome 8 are frequently associated with a variety of tumors, including prostate and breast cancer, urinary bladder carcinoma, and	

**ProposedMarker**	**Organ/Disease Sites**	**Master Grant**	**Fox Chase**	**Kimmel**	**Penn State**	**Univ Penn**	**UPCI**	**Wistar**	**Rationale**	**Status of Review**
									hematologic malignancies, among others. These abnormalities involve loss of heterozygosity at region 8p22. Analyses of these abnormalities led to the identification of the tumor suppressor gene *FEZ1*. Loss of *FEZ1* function has been suggested to play a role in the development of prostate, breast and other types of cancers, and to be involved in the progression of bladder tumors from low-grade superficial to high-grade invasive tumors	

ProposedMarker	Organ/Disease Sites	Master Grant	Fox Chase	Kimmel	Penn State	Univ Penn	UPCI	Wistar	Rationale	Status of Review
**FGF**	Adenocarcinoma<br>Brain<br>Melanoma<br>Prostate						X		**UPCI Gorelik**: Might serve as autocrine factors for proliferation of various tumor cells<br>The proposed analysis of the angiogenic factors in the blood of cancer patients is based on our hypothesis of tumor-induced cytokine chaos. Therefore, I will be very interested in testing the angiogenic factors (VEGF, PDGF and FGF) in the blood samples of cancer patients. These studies will be performed in cooperation with Dr. Anna Lokshin in a new Lumixex Core facility at UPCI. The Luinex technology provides a possibility to analyze simultaneously 10 or more molecules in the plasma of cancer patients Therefore the list of tested angiogenic molecules cfan be extended and should include IL-8, EGF, TNF and IL-6. For eac test only 50 ul of plasma is required. With appropriate technical support and funding at least 100 blood samples can be tested a day. (Melanoma, breast, prostate cancer, Glioblastoma,)	
**FGFR1**	Melanoma						X		**UPCI Kirkwood**: Predisposing & progression-associated genes in melanoma of both sporadic and familial types<br><br>Work published in the past decade from other sites, and from our center indicate that bFGF and its receptor FGFR1 as well as the signaling molecule STAT3 are important in human melanoma progression, and modifiable with antisense approaches. Recent work just published this summer has identified the importance of bRAF as a new predisposing gene for melanoma, and current work with RAF kinase inhibitors has been underway even before this finding, suggesting that there are tools emerging. We ought to focus greater effort upon this area, which is a major focus of both the UPCI and the Wistar/Penn Centers.<br><br>These genes and gene products re relevant to melanoma, and would be applied consistently in this large and growing population of patients under study both in Pittsburgh and in Philadelphia; the bFGF/FGFR1 axis relevant to angiogenesis would have much broader application, but already is being monitored in trials of the national cooperative groups (E2602 phase II trial of PEG-IFN for patients with melanoma and elevated bFGF levels).	**ADDED TO TABLE 10/09/02**
****FHIT**	Breast (2/14/03)<br>Esophageal<br>Head and neck<br>Lung	X		X			X		****Grant text**: Tumor suppressor gene that is susceptible to environmental carcinogens, including cigarette smoke><br><br>**UPCI/Grandis**: More clear in lung cancer, but Dr. Gollin's group has reported it in SCCHN. It belongs on list<br><br>**Kimmel**: The *FHIT* gene (for Fragile Histidine Triad) is a "tumor suppressor" gene that is susceptible to environmental carcinogens, including cigarette smoke. The Fhit protein encoded by this gene is absent or reduced in many common human tumors, including ~80% of cancers of the head and neck, ~80% of cancers of the lung, and ~75% of cancers of the esophagus, tumors most	**Priority Breast Marker for Kimmel 2/14/03**

ProposedMarker	Organ/Disease Sites	Master Grant	Fox Chase	Kimmel	Penn State	Univ Penn	UPCI	Wistar	Rationale	Status of Review
									commonly associated with smoking. Similarly, the Fhit protein was absent or reduced in ~85% of preneoplastic lesions of the lung and ~60% of preneoplastic lesions of the esophagus.<br><br>Loss of *FHIT* gene expression in lung cancers has been directly associated with smoking. Absence or marked reduction of Fhit protein is significantly higher in tumors of smokers than in tumors of non-smokers. Similarly, *FHIT* gene mutation in lung cancers has been associated with smoking and asbestos exposure. In addition, in patients with histories of heavy smoking, loss of *FHIT* gene expression has been observed at high frequency in esophageal cancer. Together, these studies suggest that loss of *FHIT* gene expression is an early event in the development of tobacco-related tumors. Loss of *FHIT* expression has been suggested as an early marker of malignancy in smoking-related tumors (9/02)<br><br>**Kimmel/Brenner**: FHIT is lost in a high proportion of breast tumors, particularly those that have brca1 and brca2 mutations. There is a growing body of evidence that ATR and CHK1 work with FHIT in an S-phase checkpoint pathway. There has long been evidence that ATM (related to ATR) heterozygosity may predispose to breast cancer and predict sensitivity to radiochemical treatments. (2/14/03)	

ProposedMarker	Organ/Disease Sites	Master Grant	Fox Chase	Kimmel	Penn State	Univ Penn	UPCI	Wistar	Rationale	Status of Review
****FLJ20303**	Breast						**X**		**UPCI/Kirkwood:** Designated as a priority marker 9/15/03. Will perform immunohistological/in situ assays . Paired embedded frozen samples of primary, nodal or nodal, metastatic disease tissue. Will collect density and distribution of host response elements	**Designated priority marker to measure by UPCI/Kirkwood 9/15/03**
**Gadd-45**	CNS tumors						**X**		**UPCI/Kokkinakis -** Some of the major changes in gene expression in CNS tumors under methionine stress that have attracted immediate attention include the down-regulation of Gadd45 amd mgmt. Gadd45 a gene whose expression is enhanced by DNA damaging agents such as BCNU and TMZ, is attractive given the synergy between these cytotoxic agents and MET stress in several human CNS tumor cell lines. The possible involvement of Gadd in the cytotoxic action of mda-7 is particularly interesting since Gadd may not be fully dependent on the p53 status of the tumor cell and thus may be responsive to MET stress via several mechanisms that could either involve the intact p53 mechanism or just p53 targeted genes such as **14-3-3** or **p21** or Gadd45 even when p53 itself is mutated. Gadd45 is an inhibitor of Cdk1/CyclinB1 complex and plays an important role in the induction of G2/M blocks following exposure to DNA damage. GADD-45 disrupts the interaction between Cdk-1 and CyclinB1 complex which is required for entering mitosis. The formation of the complex requires activation of the Cdk1 by inactivation of Cdc25C by 14-3-3-σ and cytosolic translocation. Activation of Cdc2 (the ultimate target of mitotic entry) by phosphorylation, allows its association with Cyclin B1 and A. This is required for cells to enter into mitosis. However DNA damage induces GADD-45, which disrupts the Cdc2-cyclin interactions forcing the cell into arrest. This arrest seems to be beneficial to the survival of the cells since it allows NER to eliminate DNA damage before the cell enters mitosis. If damage is not repaired and the cell enters mitosis it could either die or mutate. The choice between death and mutation depends on the type of damage and also on the mutational state of the cell. For example, Gadd deficient cells are easily killed with UV a situation that is exaggerated by inhibition of NER.	
**GP 100**	Melanoma							**X**	**Wistar**: Rationale pending	
**GRPR**	Head and neck<br>Lung						**X**		**UPCI/Grandis** : Work in collaboration with Dr. Siegfried has shown that GRPR is upregulated in SCCHN and lung cancer with prognostic impact.<br><br>**UPCI/Siegfried**: GRPR activation results in cell proliferation in the lung and in oral mucosa. We have shown the gene is regulated by estrogen in the lung mucosa and also that due to its location on the X chromosome, shows an imbalance of expression in airway musocal cells and lung fiberblasts between males and females, with females showing much more frequent expression. The expression patterns are also influenced by the amount of tobacco exposure and whether or not the individual has a diagnosis of lung cancer. GRPR "Expressers" (in their normal tissues, including their peripheral lymphocytes) have a greatly increased risk for lung cancer compared to "nonexpressers". Thus this phenotype may represent a test that can place an	

ProposedMarker	Organ/Disease Sites	Master Grant	Fox Chase	Kimmel	Penn State	Univ Penn	UPCI	Wistar	Rationale	Status of Review
									individual in a high-risk group for lung cancer.<br><br>We have a large dataset compiled on GRPR status in normal tissues from cancer cases and controls. We would like to also examine outcome in the cases to determine if the expresser phenotype also leads to more progression of lung cancer or lesser responses to therapy.<br><br>Measurement of GRPR in additional lung tumors and normal tissues from lung cancer cases would be of benefit to confirm and validate our hypotheses.<br><br>Genotyping of Risk Genes<br><br>There are a number of DNA repair genes and methylation genes that have recently been shown to contain polymorphisms that place individuals in elevated risk groups for tobacco carcinogenesis. We are in the process of collecting isolated DNA from all the individuals from our large GRPR dataset, and we wish to carry out genotyping for these genes on them. We also wish to examine several<br>metabolism genes as part of this study.<br><br>We hope to create a panel of risk markers that can identify those most susceptible to lung cancer.<br><br>DNA from this dataset could be made available to examine other potential risk factors. In addition, any blood collected statewide from lung cacner cases and controls could be used to increase the power and relevance to different types of lung cancer.	

ProposedMarker	Organ/Disease Sites	Master Grant	Fox Chase	Kimmel	Penn State	Univ Penn	UPCI	Wistar	Rationale	Status of Review
**GSTM1	Breast					X			Univ Penn/Rebbeck/DeMichele/Aplene: Associations of inherited genotypes with chemosensitivity/differential toxicity, clinical progression and outcome following chemotherapy in women with breast cancer (considering "traditional" biomarkers as well, such as ER/PR, Her2/neu, etc.)<br><br>Inherited genotypes in chemotherapy metabolism genes (e.g., CYP3A4, CYP3A5*3, CYP3A5*6, GSTT1, GSTM1, GSTP1 (2 SNPs), NQ01 CYP2B6, CYP2C8)	Priority breast marker per Angela DeMichele 7/15/03
GSTP*B	Prostate							X	Wistar: Rationale Pending GSTP*B is a mutant of GSTP1 (I105V)<br>GSTP*B is a mutant of GSTP1 (l105V)	ADDED 10/09/02
**GSTP1 (glutathione S-transferase	Prostate<br><br><br><br><br><br><br><br>Breast					 <br><br><br><br><br><br><br>X	 <br><br><br><br><br><br><br><br><br><br><br><br><br><br><br>X	X	Wistar: Human prostate cancer is characterized by an early and near-universal loss of expression of GSTP1. An aminoacid substitution (1I105V) in GSTP1 produces a variant enzyme with lower activity and less capability of effective detoxification.<br><br>Homozygotes for the A2 allele had a significant elevation nin risk (odds ratio = 19.2; 95% confidence interval, 2.2-157.4) compared with men who were homozygous for the A1 allele (interaction P = 0.0005).<br><br>Univ Penn/Rebbeck/DeMichele/Aplene: Associations of inherited genotypes with chemosensitivity/differential toxicity, clinical progression and outcome following chemotherapy in women with breast cancer (considering "traditional" biomarkers as well, such as ER/PR, Her2/neu, etc.)<br><br>Inherited genotypes in chemotherapy metabolism genes (e.g., CYP3A4, CYP3A5*3, CYP3A5*6, GSTT1, GSTM1, **GSTP1** (2 SNPs), NQ01 CYP2B6, CYP2C8)<br><br>UPCI/Brufsky/Hergenroeder: Reference: West, Mike: Predicting the Clinical Status of Human Breast Cancer by using Gene Expression Profiles PNAS 98(20): 11462-67, 2001	Priority breast marker per Angela DeMichele 7/15/03
**GSTT1	Breast					X			Univ Penn/Rebbeck/DeMichele/Aplene: Associations of inherited genotypes with chemosensitivity/differential toxicity, clinical progression and outcome following chemotherapy in women with breast cancer (considering "traditional" biomarkers as well, such as ER/PR, Her2/neu, etc.)<br><br>Inherited genotypes in chemotherapy metabolism genes (e.g., CYP3A4, CYP3A5*3, CYP3A5*6, GSTT1, GSTM1, GSTP1 (2 SNPs), NQ01 CYP2B6, CYP2C8)	Priority breast marker per Angela DeMichele 7/15/03
**HER-2/neu	Breast			X		X		X	Wistar: Markers of the risk of CDIS patients for invasive breast cancer and molecular targets of chemoprevention in breast intraepithelial neoplasia (Rationale added 10/9/02)<br>See also p21 and RB	Priority breast marker per Kimmel Juan

ProposedMarker	Organ/Disease Sites	Master Grant	Fox Chase	Kimmel	Penn State	Univ Penn	UPCI	Wistar	Rationale	Status of Review
									**Univ Penn/DeMichele**: Somatic markers of tumor progression or prognosis - Somatic tumor markers including p27, p21, cyclin D1, cyclin E, cox-2 enzyme, IL-6 receptor, **Her2/neu**, EGFR and mutated EGFR (vIII), MCM2, CD34, BCL-2, Bax, Topo II	**Palazzo**<br><br>**Priority breast marker per Angela DeMichele 7/15/03**

ProposedMarker	Organ/Disease Sites	Master Grant	Fox Chase	Kimmel	Penn State	Univ Penn	UPCI	Wistar	Rationale	Status of Review
**HGF**	Lung<br>Melanoma						**X**	**X**	**UPCI/Siegfried:** We have a data set of 82 lung cancer cases for which we have pathologic staging including lymph node dissection, treatment information, and outcome data. We have shown a significant association between level of **HGF** in the tumor (from frozen tumor tissue) and poor outcome (either local recurrence or metastatic disease). The effects are most pronounced in early-stage disease.<br><br>We are currently measuring c-Met level as well. HGF is an independent prognostic indicator and **HGF** level is independent of stage of disease.<br><br>HGF may be valuable to predict poor outcome in lung cancer patients with Stage IA and IB disease, and could be used as an indication for adjuvant therapy in patients who otherwise would get no treatment.<br><br>It would be extremely valuable to get additional measurements from tumors at other institutions to verify this and to increase the sample size.<br><br>**Wistar**: Overproduced and activated in many malignant tumors. The interaction of **HGF** and c-Met activates cellular signals that stimulate cancer cells to spread into neighboring normal tissues and metastasize to distant tissues. Produced by melanoma cells and serum levels have been associated with low survival and with late stages.	**ADDED 10/09/02**
**HLA-2**	Melanoma							**X**	**Wistar:** Rationale pending	
**HLA-G**	Breast<br>Prostate						**X**		**UPCI/Chatta-Shurin:** HLA-G is a non-classical MHC class Ib molecule with highly limited tissue distribution that has been attributed chiefly immune regulatory functions. HLA-G has been proposed to regulate immune responses during pregnancy playing a crucial role in maintaining an immuno-privileged environment at the materno-fetal interface. Similarly, HLA-G expression in tumor cells may favor their escape from host immune surveillance. HLA-G interacts with killing inhibitory receptors (KIR), hereby rescuing HLA-G expressing cells from NK cell attack. The inhibitory effect of HLA-G on priming of cytotoxic T cells has been shown to be directed against both CD8 and CD4 T cells. Recently it has been shown that HLA-G also modifies the function of dendritic cells via interactions with the paired immunoglobulin-like transcript (ILT) receptors. Thus, it is conceivable to consider the potential function of HLA-G as a new strategy of cancer cells to escape from immunosurveillance. For instance, HLA-G was activated at the transcriptional level and was up-regulated at high frequencies in human breast cancer, where it may impair efficient antitumor immunity. It has been also reported that a few HLA-G-positive cells within a population of HLA-G-negative tumor cells exerted significant immune inhibitory effects confirming that the aberrant expression of HLA-G may contribute to immune escape in different cancers.	

ProposedMarker	Organ/Disease Sites	Master Grant	Fox Chase	Kimmel	Penn State	Univ Penn	UPCI	Wistar	Rationale	Status of Review
****HPC2**	Prostate					X			**Univ Penn/Rebbeck**: Associations of inherited genotypes with clinical progression and outcome in men with prostate cancer. Inherited genotypes in hormone, cytokine, and DNA damage/repair pathways (e.g., SRD5A2, CYP3A4, CYP3A5, AR, HPC2, IL6, SOD2	**Priority prostate marker per Upenn 7/15/03**
**Hras**	Breast<br>Melanoma					X			**Univ Penn/Gerrero (Weber)**: Somatic mutations – Genes known to be involved in predisposition and progression of breast and/or melanoma (Somatic mutations PTEN, CDKN2A, ARF, CDK4, TP53, RB1, Hras, Braf, Cyclin D1)	
**HSD3B2**	Breast					X			**Univ Penn/Weber/Rebbeck**: **Breast** cancer – Candidate low penetrance susceptibility alleles Germline variants genes involved in hormone metabolism, DNA damage response and immune surveillance (CYP3A4, CYP3A5, CYP17, COMT, HSD3B2, CYP19, IL1, IL1R, TNFa,, IL10, IL6, IL12, XPD,XRCC2, XRCC3)	
****IGF-1**	Breast<br><br><br><br><br><br>Prostate					**X**<br><br><br><br><br><br>**X**			**Univ Penn/DeMichele**: Predictors of response or late effects in breast cancers (Including: **IGF-1**, ER polymorphisms - PvuII, Xbal, Codon 325 C>G, TA repeat, Vitamin D receptor (VDR) polymorphisms - Taq1, Fokl start codon Collagen I alpha (COLIA1) polys - MSC1, Sp1G>T, IL-6 polys - CA repeat, Nt-634 C>G, -174 G>C TGF-beta polys -509C>T, -869C>T)<br><br>**Univ Penn/Nathanson**: Genotypes associated with recurrence of prostate cancer in collaboration with Duke Univ. (J. Schildkraut) Inherited Genotypes in androgen metabolisn and insulin-like growth factor pathway (SRD5A2, AR, CYP3A4, CYP3A5, IGFBP3, IGF1, VDR)	**Priority marker per J Glick Upenn**<br><br>**Priority breast marker per Angela DeMichele 7/15/03**
**IGFBP3**	Prostate					**X**			**Univ Penn/DuPont Guerry**: Interited genotypes in androgen metabolism and insulin-like growth factor pathway (SRD5A2, AR, CYP3A4, CYP3A5, **IGFBP3**, IGF1, VDR.	**ADDED 10/09/02**
**IL-1**	Breast					**X**			**Univ Penn/Weber/Rebbeck**: **Breast** cancer – Candidate low penetrance susceptibility alleles Germline variants genes involved in hormone metabolism, DNA damage response and immune surveillance (CYP3A4, CYP3A5, CYP17, COMT, HSD3B2, CYP19, IL1, IL1R, TNFa,, IL10, IL6, IL12, XPD,XRCC2, XRCC3)	
**IL-10**	Breast<br>Melanoma					**X**		 <br><br><br><br>**X**	**Univ Penn/Weber/Rebbeck**: **Breast** cancer – Candidate low penetrance susceptibility alleles Germline variants genes involved in hormone metabolism, DNA damage response and immune surveillance (CYP3A4, CYP3A5, CYP17, COMT, HSD3B2, CYP19, IL1, IL1R, TNFa,, IL10, IL6, IL12, XPD,XRCC2, XRCC3)<br><br>**Wistar**: Produced by melanoma cells and serum levels have been associated with low survival and with late stages	

ProposedMarker	Organ/Disease Sites	Master Grant	Fox Chase	Kimmel	Penn State	Univ Penn	UPCI	Wistar	Rationale	Status of Review

ProposedMarker	<u>Organ/Disease Sites</u>	Master Grant	Fox Chase	Kimmel	Penn State	Univ Penn	UPCI	Wistar	Rationale	<u>Status of Review</u>
**IL-12**	Breast					**X**			<u>**Univ Penn/Weber/Rebbeck**</u>: **Breast** cancer – Candidate low penetrance susceptibility alleles Germline variants genes involved in hormone metabolism, DNA damage response and immune surveillance (CYP3A4, CYP3A5, CYP17, COMT, HSD3B2, CYP19, IL1, IL1R, TNFa,, IL10, IL6, IL12, XPD,XRCC2, XRCC3)	
**IL-1R**	Breast					**X**			<u>**Univ Penn/Weber/Rebbeck**</u>: **Breast** cancer – Candidate low penetrance susceptibility alleles Germline variants genes involved in hormone metabolism, DNA damage response and immune surveillance (CYP3A4, CYP3A5, CYP17, COMT, HSD3B2, CYP19, IL1, IL1R, TNFa,, IL10, IL6, IL12, XPD,XRCC2, XRCC3)	
****IL-6**	Breast<br>Melanoma<br>Prostate					**X**	**X**	**X**	<u>**UPCI-Gorelik:**</u> The proposed analysis of the angiogenic factors in the blood of cancer patients is based on our hypothesis of tumor-induced cytokine chaos.<br><br>The Luminex core facility at UPCI provides a possibility to analyze simultaneously 10 or more molecules in the plasma of cancer patients. Therefore the list of angiogenic molecules could be extended to include IL-8, EGF, TNF and IL-6, as well as VEGF, PDGF and FGF.<br><br><u>**Wistar**</u>: . Produced by **melanoma** cells and serum levels have been associated with low survival and with late stages<br><br><u>**Univ Penn/Rebbeck**</u>: Associations of inherited genotypes with clinical progression and outcome in men with **prostate** cancer. (Inherited genotypes in hormone, cytokine, and DNA damage/repair pathways (e.g. SRD5A2, CYP3A4, CYP3A5, AR, HPC2, IL6, SOD2)<br><br><u>**Univ Penn/Weber/Rebbeck**</u>: **Breast** cancer – Candidate low penetrance susceptibility alleles Germline variants genes involved in hormone metabolism, DNA damage response and immune surveillance (CYP3A4, CYP3A5, CYP17, COMT, HSD3B2, CYP19, IL1, IL1R, TNFa,, IL10, IL6, IL12, XPD,XRCC2, XRCC3)	**Priority marker per J. Glick Upenn**<br><br>**Priority breast marker per Angela DeMichele 7/15/03**
**IL-6 Receptor**	Breast					**X**			<u>**Univ Penn/DeMichele**</u>: Somatic markers of tumor progression or prognosis - Somatic tumor markers including p27, p21, cyclin D1, cyclin E, cox-2 enzyme, **IL-6 receptor**, Her2/neu, EGFR and mutated EGFR (vIII), MCM2, CD34, BCL-2, Bax, Topo II	
**IL-7**	???									
**IL-8**	Melanoma						**X**		<u>**UPCI-Gorelik:**</u> The proposed analysis of the angiogenic factors in the blood of cancer patients is based on our hypothesis of tumor-induced cytokine chaos.	

ProposedMarker	Organ/Disease Sites	Master Grant	Fox Chase	Kimmel	Penn State	Univ Penn	UPCI	Wistar	Rationale	Status of Review
									The Luminex core facility at UPCI provides a possibility to analyze simultaneously 10 or more molecules in the plasma of cancer patients. Therefore the list of angiogenic molecules could be extended to include IL-8, EGF, TNF and IL-6, as well as VEGF, PDGF and FGF.	
****K1 67**	Lung						**X**		**UPCI/Belani**: Ki-67 is a nonhistone protein expressed in the nucleus of cells in active division. Used as a cell proliferation marker, Ki-67 expression could be a helpful prognostic indicator of tumor aggression and patient survival in several malignancies, including lung cancer.<br><br>**UPCI/Grandis**: Non-specific with little clear correlative clinical data. I am lukewarm as I don't think it will be very informative but may reflect a general estimate of proliferation in the tumor.	**Priority melanoma marker Upenn 7/15/03**
	Melanoma					**X**			**Univ Penn/DuPont Guerry**: Somatic markers of progression, prognosis, and proliferation, by histology (ki-67, p16 and its methylation state, B-raf)	
**KiSS1**	Breast<br>Melanoma	**X**			**X**				****PSU/Hershey**: KiSS1 was identified by subtractive hybridization comparing metastatic and nonmetastatic human melanoma cell lines. Expression has been observed in melanocytes and radial growth phase-derived cell lines, but not vertical growth phase-metastasis-derived melanoma cell lines. Transfection into human melanoma and breast carcinoma cell lines suppressed metastasis in xenograft models.	
**LAMA**	Melanoma							**X**	**Wistar**: Human lymphocyte activation melanoma antigen considered to be a shared tissue-specific antigen which may be useful in immunodiagnosis and immunotherapy of melanoma.	

**ProposedMarker**	**<u>Organ/Disease Sites</u>**	**Master Grant**	**Fox Chase**	**Kimmel**	**Penn State**	**Univ Penn**	**UPCI**	**Wistar**	**Rationale**	**<u>Status of Review</u>**
**LMP2**	Melanoma<br>Prostate						**X**		**<u>UPCI/Chatta-Shurin</u>-Dhir-Ferrone:** The above genes are components of the antigen processing machinery, which have been shown to be disrupted in Melanoma (Ferrone et al). In collaboration with Dr Ferrone (RPCI), we are undertaking an investigation of these genes in prostate cancer both at the cDNA, as well as at the protein level.	
**MART-1**	Melanoma							**X**	**<u>Wistar</u>:** Rationale pending	
**MCM2**	Breast					**X**			**<u>Univ Penn/DeMichele</u>**: Somatic markers of tumor progression or prognosis - Somatic tumor markers including p27, p21, cyclin D1, cyclin E, cox-2 enzyme, IL-6 receptor, Her2/neu, EGFR and mutated EGFR (vIII), **MCM2**, CD34, BCL-2, Bax, Topo II	
**MDA-7**	Breast<br>CNS tumors<br>Colon<br>Lung<br>Melanoma<br>Prostate					**X**	**X**		**<u>UPCI/Kokkinakis</u>:**<br>Some of the major changes in gene expression in CNS tumors under methionine stress that have attracted immediate attention include the upregulation of Mda-7, Cradd and Bak and the downregulation of Gadd45 and mgmt. . Mda-7 is a unique gene that is selectively upregulated during the process of terminal differentiation and irreversible growth arrest of melanoma cells . This gene is a member of IL-10 family and its product is also known as IL24. When expressed by means of Ada.mda-7 infection of melanoma, breast carcinoma, prostate, small lung and colon carcinoma induces apoptosis following a G2/M arrest. However, expression of the gene in normal cells does not result in toxicity. Most importantly, the cytotoxic effect of the gene product, MDA-7, is not dependent on the p53 status and functionality of the p53 pathway and induces apoptosis by upregulation of BAX and BAK (modest) and also extensive dowregulation of Bcl-XL (3-9 fold). Recent evidence suggests that MDA-7 induces apoptosis via the activation of **p38 MAPK** pathway, at least in melanomas. Activation of p38MAPK pathway mediated by TNF, ceramide, sodium salicylates and UV, has been associated with apoptosis. This is further supported by the inhibition of apoptosis with the use of p38 MAPK inhibitor SB203580. Infection of tumor cells with Ad.mda-7 appears to dowregulate the activity of **bcl-2** promoter. In normal cells the induction of GADD pathway by Ad.mda-7 infection is not operative which explains the resistance of melanocytes to MDA-7 expression<br><br>**<u>Univ/Penn/DeMichele</u>**: Somatic markers of differentiation – Mitf and MDA-7 for melanoma	
**MGMT**	CNS tumors<br>Pancreas						**X**		**<u>UPCI/Kokkinakis -</u>** Some of the major changes in gene expression in CNS tumors under methionine stress that have attracted immediate attention include the upregulation of **mda-7** **Cradd** and **Bak** and the downregulation of **Gadd45** and **mgmt.**<br><br>This protein is responsible for the resistance of tumors to DNA damage induced by common alkylating drugs. Removal of the MGMT activity either with the use of MGMT inactivators or by	

ProposedMarker	Organ/Disease Sites	Master Grant	Fox Chase	Kimmel	Penn State	Univ Penn	UPCI	Wistar	Rationale	Status of Review
									induction of methionine stress results in sensitization of tumors to chemotherapy. This particularly important in pancreatic tumors where MGMT is extremely high.	
****MITF**	Melanoma					X			**Univ Penn/DuPont Guerry:** Somatic markers of differentiation - Mitf, MDA-7	**Priority melanoma marker Upenn 7/15/03**
**MLH1**	Gastrointestinal						X		**UPCI Wood/Sepulveda:** DNA mismatch repair genes. MLH1 and MSH2 are two key proteins that carry out DNA repair of base pair mismatches and insertion or deletion loops that may occur during DNA replication. If left unrepaired these DNA strand alterations are a known cause of gene mutations (manifested as microsatellite instability) that may affect critical regulatory proteins leading to an increased risk of malignancy development and progression. Deficiency (loss of expression) of MLH1 or MLH1 is the cause of most cases of hereditary non-polyposis colorectal cancer (HNPCC).<br>Loss of expression of MLH1 or MSH2 has also been found in sporadic cancers, especially those of stomach (30%) and colon (15-20%), although subsets of carcinomas from other organs such as prostate, endometrium and pancreas may also display loss of expression of MLH1 or MSH2. Patients with colorectal or stomach tumors laking MLH1 or MSH2 exhibit improved survival, in part associated with a lesser frequency of metastatic disease. In vitro, tumor cells with mismatch repair deficiency have been shown to have some tolerance to 5-FU, suggesting that that the mismatch repair system might be one mechanism for tumor resistance to 5-FU.<br>Because of the relatively frequent alteration in some GI cancers, expression analysis of these proteins on a large number of gastrointestinal carcinomas would help define their application as biomarkers in establishing disease prognosis and treatment selection. The expression of MLH1 and MSH2 is easily detected by immunohistochemistry on surgical or biopsy specimens.	
**MMP2**	Melanoma					X			**Univ Penn/DuPont Guerry**: Somatic markers of invasion and adhesion (MMP-2, MUC-18, alpha V/beta 3, CD9)	

**ProposedMarker**	**Organ/Disease Sites**	**Master Grant**	**Fox Chase**	**Kimmel**	**Penn State**	**Univ Penn**	**UPCI**	**Wistar**	**Rationale**	**Status of Review**
**MSH2**	Colorectal Gastrointestinal						**X**		**UPCI Wood/Sepulveda:** DNA mismatch repair genes. MLH1 and MSH2 are two key proteins that carry out DNA repair of base pair mismatches and insertion or deletion loops that may occur during DNA replication. If left unrepaired these DNA strand alterations are a known cause of gene mutations (manifested as microsatellite instability) that may affect critical regulatory proteins leading to an increased risk of malignancy development and progression. Deficiency (loss of expression) of MLH1 or MLH1 is the cause of most cases of hereditary non-polyposis colorectal cancer (HNPCC).<br>Loss of expression of MLH1 or MSH2 has also been found in sporadic cancers, especially those of stomach (30%) and colon (15-20%), although subsets of carcinomas from other organs such as prostate, endometrium and pancreas may also display loss of expression of MLH1 or MSH2.<br>Patients with colorectal or stomach tumors laking MLH1 or MSH2 exhibit improved survival, in part associated with a lesser frequency of metastatic disease. In vitro, tumor cells with mismatch repair deficiency have been shown to have some tolerance to 5-FU, suggesting that that the mismatch repair system might be one mechanism for tumor resistance to 5-FU.<br>Because of the relatively frequent alteration in some GI cancers, expression analysis of these proteins on a large number of gastrointestinal carcinomas would help define their application as biomarkers in establishing disease prognosis and treatment selection. The expression of MLH1 and MSH2 is easily detected by immunohistochemistry on surgical or biopsy specimens.	
****MSR1**	**Prostate**					**X**			**Univ Penn/Rebbeck** Genotypes associated with recurrence and outcomes of prostate cancer in collaboration, including inherited genotypes in hereditary prostate cancer genes, androgen metabolisn and insulin-like growth factor pathway (HPC2, MSR1, RNASEL, SRD5A2, AR, CYP3A4, CYP3A5, IGFBP3, IGF1, VDR). (Added 2/12/03)	**Added 2/13/03** **Priority prostate marker per UPenn**
**MUC-18**	Melanoma					**X**			**Univ Penn/DuPont Guerry**: Somatic markers of invasion and adhesion (MMP-2, MUC-18, alpha V/beta 3, CD9)	
**NCOR**	Breast						**X**		**UPCI/Brufsky/Hergenroeder: Priority marker – rationale to follow**	**Added to list 04/14/03**
**NF1**	REMOVE FROM LIST		**X**						**REMOVE - FCCC**:	
**NF2**	REMOVE FROM LIST		**X**						**REMOVE - FCCC**:	
**NFKBIA (IKB alpha)**	Breast						**X**		**UPCI/Brufsky/Hergenroeder:** Major histocompatability complex enhancer – binding protein MAD3, transcriptional regulator, gradual decline from DCIS to invasive breast cancer. IK (alpha) sequesters NFKb in the cytoplasm in an inactive form, therefore loss of IK (alpha) expression may lead to activation of the NFKb pathway (Reference Porter, Dale: A Sage View of Breast	**Added to list 04/14/03**

ProposedMarker	<u>Organ/Disease Sites</u>	Master Grant	Fox Chase	Kimmel	Penn State	Univ Penn	UPCI	Wistar	Rationale	<u>Status of Review</u>
									Tumor Progression. Cancer Research 61: 5697-5702, 2001	
**NQO1**	Breast					**X**			**<u>Univ Penn/Rebbeck/DeMichele/Aplene</u>**: Associations of inherited genotypes with chemosensitivity/differential toxicity, clinical progression and outcome following chemotherapy in women with breast cancer (considering "traditional" biomarkers as well, such as ER/PR, Her2/neu, etc.)<br><br>Inherited genotypes in chemotherapy metabolism genes (e.g., CYP3A4, CYP3A5*3, CYP3A5*6, GSTT1, GSTM1, GSTP1 (2 SNPs), NQ01 CYP2B6, CYP2C8)	
**p300**	Breast						**X**		**<u>UPCI/Brufsky/Hergenroeder</u>: Priority marker – rationale to follow**	**Added to table 04/14/03**
**P-Cadherin**	Melanoma							**X**	**<u>Wistar:</u>** During melanoma development, loss of E-cadherins expression is observed. It is accompanied by a parallel gain of N-cadherin expression that facilitates migration of melanoma cells from epidermis.	
**PDGF**	Brain<br>Melanoma<br>Adenocarcinomas						**X**		**<u>UPCI/Gorelik</u>**: PDGF Stimulates periendothelial cells	
****PDGR Receptor (alpha and beta)**	Prostate			**X**					**<u>Kimmel/TJU/Dicker: Identified as priority prostate marker by Adam Dicker 7/15/03</u>**	**Added to master table 7/15/03**
**Phakomatosis genes (10 in number)**	REMOVE FROM LIST		**X**						**<u>REMOVE - FCCC</u>**:	
**Phytoestrogens (Urinary excretion)**	Breast cancer							**X**	**<u>Wistar: Rationale pending</u>**	
**Pim-1**	Prostate<br>Colorectal<br>Leukemia<br>Stomach						**X**		**<u>UPCI/Wood-Sepulveda</u>**:<br>The pim family of protooncogene consists of at least three members encoding related serine-threonine protein kinases. The first protein described was pim-1. During embryonic development the pim-1 genes are expressed in cells of the immune and central nervous system and in epithelia.<br><br>Although little is known about the role of these proteins in epithelia, Pim-1 was recently described in association with epithelial malignancy. The functional effects of pim-1 include protection of thymocytes against glucocorticoid-induced apoptosis and promotion of cell proliferation in several IL-3 or IL-6 dependent hematopoietic cell lines. In B cells activation of NFKB leads to increased pim-1 expression. Pim-1 is important in tumor development and progression and the pim-1 protein is increased in adult acute leukemia and in chronic lymphocytic **leukemia**. Pim-1	

ProposedMarker	Organ/Disease Sites	Master Grant	Fox Chase	Kimmel	Penn State	Univ Penn	UPCI	Wistar	Rationale	Status of Review
									appears to function as a true oncogene since its increased expression in transgenic mice leads to increased incidence of tumors.<br><br>Pim-1 expression is an independent marker of tumor recurrence in prostate carcinomas and low levels are found in normal prostate epithelial cells. Pim-1 is expressed in a high percentage of gastric carcinomas and gastric cancer cell lines while low levels are found in the normal gastric mucosa. In contrast pim-1 is rarely expressed in **colon** carcinomas.<br><br>The expression of pim-1 is evaluated by established immunohistochemical methods of tumor sections. Similar to **prostate** cancer, it is likely that Pim-1 expression correlates with tumor behavior in **gastrointestinal** malignancy, and may be useful as a marker to help predict tumor recurrence.	

ProposedMarker	Organ/Disease Sites	Master Grant	Fox Chase	Kimmel	Penn State	Univ Penn	UPCI	Wistar	Rationale	Status of Review
**Plastin T**	CTCL							**X**	**Wistar:** Not expressed in normal T-cell - expressed in a high percentage of CTCL patients. Confirmed by array and QRT-PCR	
****PR (PGR)**	Breast						**X**		**Univ Penn/Rebbeck/DeMichele/Aplene**: Associations of inherited genotypes with chemosensitivity/differential toxicity, clinical progression and outcome following chemotherapy in women with breast cancer (considering "traditional" biomarkers as well, such as **ER/PR**, Her2/neu, etc.) Inherited genotypes in chemotherapy metabolism genes (e.g., CYP3A4, CYP3A5*3, CYP3A5*6, GSTT1, GSTM1, GSTP1 (2 SNPs), NQ01 CYP2B6, CYP2C8) Progesterone receptor	**ADDED 10/09/02**<br><br>**Priority breast marker per Kimmel Juan Palazzo**
**PTCH**	REMOVE FROM LIST		**X**						**REMOVE - FCCC:** Currently on their "watch list" – requested rationale<br><br>[Drosophila patched]	
**PTEN**	Breast<br>Melanoma<br>Thyroid		**X**			**X**			**FCCC**: Defects in PTEN are a cause of Cowden syndrome (CS). CS is an autosomal dominant cancer predisposition syndrome associated with elevated risk for tumors of the breast, thyroid and skin cancers. [UniGene Hs.10712] [C. elegans daf-18] {Drosophila Pten]<br><br>**Univ Penn/Gerrero (Weber)**: Somatic mutations – Genes known to be involved in predisposition and progression of breast and/or melanoma (Somatic mutations PTEN, CDKN2A, ARF, CDK4, TP53, RB1, Hras, Braf, Cyclin D1)<br><br>**Univ Penn/Gerrero (Weber)**: Genes known to be involved in predisposition and progression of breast and/or melanoma (Germline mutations BRCA-1, BRCA-2, PTEN, CDKN2, ARF, CDK4)	
**RB1**	Breast<br>Melanoma		**X**			**X**		**X**	**Univ Penn/Gerrero (Weber)**: Somatic mutations – Genes known to be involved in predisposition and progression of breast and/or melanoma (Somatic mutations PTEN, CDKN2A, ARF, CDK4, TP53, RB1, Hras, Braf, Cyclin D1)<br><br>**Wistar**: Markers of the risk of CDIS patients for invasive breast cancer and molecular targets of chemoprevention in breast intraepithelial neoplasia (Rationale added 10/9/02) Retinoblastoma 1 [unigene Hs.75770] [C.elegans lin-35]<br><br>**REMOVE - FCCC**: Currently on their watch list – requested rationale	
****RNASEL**	**Prostate**					**X**			**Univ Penn/Rebbeck:** Genotypes associated with recurrence and outcomes of prostate cancer in collaboration, including inherited genotypes in hereditary prostate cancer genes, androgen metabolisn and insulin-like growth factor pathway (HPC2, MSR1, RNASEL, SRD5A2, AR,	**Added 2/13/03**<br>**Priority prostate**

**ProposedMarker**	**Organ/Disease Sites**	**Master Grant**	**Fox Chase**	**Kimmel**	**Penn State**	**Univ Penn**	**UPCI**	**Wistar**	**Rationale**	**Status of Review**
									CYP3A4, CYP3A5, IGFBP3, IGF1, VDR). (Added 2/12/03)	**marker per UPenn**
**SCGB3A1**	Breast						**X**		**UPCI/Brufsky/Hergenroeder:** Secretoglobulin family 3A member 1 (HIN1). Novel growth inhibitory cytokine, hypermethylated in a large fraction of breast cancer that may play a role in epithelial cell proliferation and branching morphogenesis (Reference: Porter, Dale: A Save View of Breast Tumor Progression. Cancer Research 61: 5697-5702, 2001	
**SERPINB5 (MASPIN) (p15)**	Breast						**X**		**UPCI/Brufsky/Hergenroeder:** Inverse relationship with ER function (Reference West, Mike: Predicting the Clinical Status of Human Breast Cancer by Using Chip Technology. Anti-cancer Research 21: 3799-3806, 2001	**Added to list 04/14/03**
**SMAD4**	REMOVE FROM LIST		**X**						**REMOVE – FCCC**<br><br>(DPC4) (juvenile polyposis) [C. elegans daf-3]	
****SNX17**	Breast						**X**		**UPCI/Brufsky –** Designated as a priority marker 9/15/03 – assays will be conducted and gene expression results available	**Added 9/15/03 Priority marker by Adam Brufsky – Assays will be performed**
**SOD2**	Prostate<br>Breast					X	 X		**Univ Penn/Rebbeck**: Associations of inherited genotypes with clinical progression and outcome in men with prostate cancer<br><br>Inherited genotypes in hormone, cytokine, and DNA damage/repair pathways (e.g., SRD5A2, CYP3A4, CYP3A5, AR, HPC2, IL6, SOD2<br><br>**UPCI/Brufsky/Hergenroeder** – Mitochondrial superoxide dismutase decreased level in cancer cells is well documented and it may contribute to the high level of reactive oxygen species and subsequent oxidative stress characteristics of breast cancer (Reference: Porter, Dale A: A Sage view of breast tumor progression. Cancer Research 61: 5697, 2001)	**Added breast as disease site for this marker 4/14/04**
**SRC-1**	Breast						X		**UPCI/Brufsky/Hergenroeder** – Priority marker – rationale to follow	**Added to list 04/14/03**
****SRD5A2**	Prostate					X			**Univ Penn/Rebbeck**: Associations of inherited genotypes with clinical progression and outcome in men with prostate cancer. Inherited genotypes in hormone, cytokine, and DNA damage/repair pathways (e.g., SRD5A2, CYP3A4, CYP3A5, AR, HPC2, IL6, SOD2	**Priority prostate marker Upenn**

ProposedMarker	Organ/Disease Sites	Master Grant	Fox Chase	Kimmel	Penn State	Univ Penn	UPCI	Wistar	Rationale	Status of Review
									**Univ Penn/Nathanson**: Genotypes associated with recurrence of prostate cancer in collaboration with Duke Univ. (J. Schildkraut) Inherited Genotypes in androgen metabolism and insulin-like growth factor pathway (SRD5A2, AR, CYP3A4, CYP3A5, IGFBP3, IGF1, VDR)	**7/15/03**
**STAT 3 and Phosphorylated STAT3**	Melanoma						**X**		**UPCI/Steinman**: Some people think that increased stat3 expression is a tumor marker. While I think that this is a fairly unsophisticated view, it is conceivable that increased phosphotyrosine-705-stat3 could be a biomarker in some cases.<br><br>**UPCI/Kirkwood**: Predisposing & progression-associated genes.<br><br>**UPCI/Grandis**: Upregulated in SCCHN and now reported as a prognostic marker. I would strongly argue for measurement of phosphorylated STAT3 as well (a different Ab) since it reflects activation and is more closely associated with transformation and tumor progression.	

ProposedMarker	Organ/Disease Sites	Master Grant	Fox Chase	Kimmel	Penn State	Univ Penn	UPCI	Wistar	Rationale	Status of Review
**STAT4**	Cutaneous T-cell lymphoma							**X**	**Wistar:** Lost CTCL cells, expressed in normal T-cells	
**STK11 (LKB1) CDH1**	REMOVE FROM LIST		**X**						**REMOVE - FCCC**	
**Tapasin**	Melanoma Prostate						**X**		**UPCI/Chatta-Shurin-Dhir-Ferrone:** The above genes are components of the antigen processing machinery, which have been shown to be disrupted in Melanoma (Ferrone et al). In collaboration with Dr Ferrone (RPCI), we are undertaking an investigation of these genes in prostate cancer both at the cDNA, as well as at the protein level.	
**TCL1**	Leukemia	**X**		**X**					****Grant text**: Activation of the TCL1 oncogene, resulting from chromosomal rearrangements involving region 14q32.1, is considered to be a causative event in the development of T-cell leukemias (chronic lymphocytic leukemia and prolymphocytic leukemia) and B-cell chronic lymphocytic leukemia<br><br>**Kimmel**: Rearrangements at region q32.1 of chromosome 14 are the most commonly observed cytogenetic features of mature T-cell leukemias (such as T-cell chronic lymphocytic leukemia and prolymphocytic leukemia, T-CLL/T-PLL). These rearrangements include both translocations and inversions. In patients with mature T-cell leukemias, analyses of the chromosomal rearrangements led to the identification of the *TCL1* gene. This gene is normally not expressed in mature T cells, but it was found to be expressed in almost 100% of cases of T-CLL/T-PLL studied. Activation of the *TCL1* oncogene, resulting from chromosomal rearrangements involving region 14q32.1, is considered to be a causative event in the development of T-cell leukemias. This gene also seems to be important in the pathogenesis of B-cell chronic lymphoctyic leukemia, the most common human leukemia.	
**Telomerase**	Melanoma	**X**			**X**	**X**			****PSU/Hershey /Clawson**: Telomerase is a ribonucleoprotein complex, containing a structural RNA and a catalytic subunit. Telomerase functions to prevent telomeres during cellular DNA replication. Its activity is generally shut down in differentiated cells, thereby limiting cellular replicative potential. It is inappropriately activated in epithelial cancer cells, allowing unlimited replicative potential. The mRNA coding for its catalytic subunit will serve as a general marker for transformed epithelial cells of essentially all derivations. Telomerase is a general marker for transformed epithelial cells of essentially all derivations<br><br>**Univ Penn/DuPont Guerry** : Somatic marker of senescence	
**TGF-alpha**	Head and neck						**X**		**UPCI Grandis**: We have shown that upregulation of this EGFR ligand plays a role in SCCHN tumorigenesis and TGF-alpha can also serve as a therapeutic target.	

ProposedMarker	<u>Organ/Disease Sites</u>	Master Grant	Fox Chase	Kimmel	Penn State	Univ Penn	UPCI	Wistar	Rationale	<u>Status of Review</u>
****TGF-beta**	Breast					**X**			**<u>Univ Penn/deMichele</u>**	**Priority marker per J. Glick Upenn**
**TIA-1**	Melanoma					**X**			**<u>Univ Penn/DuPont Guerry</u>**: Somatic Markers of immunogenicity (TIA-1, CD8, CD4)	
**TNF**	Melanoma						**X**		**<u>UPCI-Gorelik:</u>** The proposed analysis of the angiogenic factors in the blood of cancer patients is based on our hypothesis of tumor-induced cytokine chaos.<br><br>The Luminex core facility at UPCI provides a possibility to analyze simultaneously 10 or more molecules in the plasma of cancer patients. Therefore the list of angiogenic molecules could be extended to include IL-8, EGF, TNF and IL-6, as well as VEGF, PDGF and FGF.	
**TNFa**	Breast					**X**			**<u>Univ Penn/Weber/Rebbeck</u>**: **Breast** cancer – Candidate low penetrance susceptibility alleles Germline variants genes involved in hormone metabolism, DNA damage response and immune surveillance (CYP3A4, CYP3A5, CYP17, COMT, HSD3B2, CYP19, IL1, IL1R, TNFa,, IL10, IL6, IL12, XPD,XRCC2, XRCC3)	
**TNFSF10**	Leukemia							**X**	**<u>Wistar</u>**: TNFSF10, TRAIL, DUSP1 and CDID are all over-expressed In CTCL T-cells	**ADDED 10/09/02**
**TOPO II A**	Breast					**X**			**<u>Univ Penn/DeMichele</u>**: Somatic markers of tumor progression or prognosis **-** Somatic tumor markers including p27, p21, cyclin D1, cyclin E, cox-2 enzyme, IL-6 receptor, Her2/neu, EGFR and mutated EGFR (vIII), MCM2, CD34, BCL-2, Bax, **Topo II**	
**TOPO II B**	Breast					**X**			**<u>Univ Penn/DeMichele</u>**: Somatic markers of tumor progression or prognosis **-** Somatic tumor markers including p27, p21, cyclin D1, cyclin E, cox-2 enzyme, IL-6 receptor, Her2/neu, EGFR and mutated EGFR (vIII), MCM2, CD34, BCL-2, Bax, **Topo II**	
****TP53**	Brain<br>Breast<br>Colorectal<br>Head and neck<br>Lung<br>Melanoma<br>Pancreas		**X**			**X**	**X**	**X**	**<u>FCCC</u>**: Rationale pending<br><br>**<u>Univ Penn/Gerrero (Weber</u>**): Somatic mutations – Genes known to be involved in predisposition and progression of breast and/or melanoma (Somatic mutations PTEN, CDKN2A, ARF, CDK4, TP53, RB1, Hras, Braf, Cyclin D1)<br><br>**<u>UPCI/Finkelstein</u>**: TP53 is one of the most important tumor suppressor genes and has been intensively studied for over ten years by many groups. TP53 is known as the guardian of the cell as it plays a critical role in regulation of the cell cycle, DNA repair, maintenance of the extracellular matric and homeostatic growth control. Mutations involving TP53 have been well	**Priority breast marker per Kimmel Juan Palazzo**

ProposedMarker	Organ/Disease Sites	Master Grant	Fox Chase	Kimmel	Penn State	Univ Penn	UPCI	Wistar	Rationale	Status of Review
									characterized and represent the most common cancer mutation being present in over 50% of all human tumors. It is especially mutated in common epithelial cancers including colon, pancreas and lung cancer. Most importantly, mutational change involving TP53 has been repeatedly shown in many studies to be an important predictive marker of tumor biological aggressiveness, treatment responsiveness and patient outcome. These well established facts provide support for its clinical use to assist cancer diagnosis and prognostication.<br><br>**UPCI/Grandis**: Established SCCHN marker.<br><br>**Wistar**: Pending	
**TS**	Lung						**X**		**UPCI/Belani**: Thymidylate synthase (TS) is a key enzyme for DNA synthesis, which provides de novo thymidylate for DNA synthesis, catalyzing the methylation of deoxyuridine monophosphate (dUMP) to deoxythymidine monophosphate (dTMP) with the 5,10-methylenetetrahydrofolate (5,10-CH_2FH_4) as a folate cofactor. TS is a target for 5-fluorouracil (5-FU), which is one of the most popular anticancer drugs and its effect is related to its active metabolite, 5-fluoro-2'-deoxyuridine 5'-monophosphate (FdUMP). FdUMP forms a tight-binding covalent ternary complex with TS and 5,10-CH_2FH_4 and thereby blocks the DNA synthetic process. Recently, many clinical studies have revealed that TS expression in various tumors, especially in gastro-intestinal malignancies, has an influence on prognosis as well as a drug resistance to 5-FU based chemotherapies. Many of these studies revealed that high levels of TS expression in tumor cells are related to poor prognosis and resistance to 5-FU. High expression of TS has been linked to poor prognosis for patients with lung cancer, especially adenocarcinoma. When combined with other such markers, this could provide valuable information to risk-stratify patients with lung cancer.	
**TSC1**	NEED TO CLARIFY		**X**						**FCCC:** TSC1 and TSC2 mutations in tuberous sclerosis	
**TSC2**	NEED TO		**X**						**FCCC:** TSCI and TSC2 mutations in tuberous sclerosis	

**ProposedMarker**	**<u>Organ/Disease Sites</u>**	**Master Grant**	**Fox Chase**	**Kimmel**	**Penn State**	**Univ Penn**	**UPCI**	**Wistar**	**Rationale**	**<u>Status of Review</u>**
[Drosophila gigas]	<u>CLARIFY</u>									

ProposedMarker	Organ/Disease Sites	Master Grant	Fox Chase	Kimmel	Penn State	Univ Penn	UPCI	Wistar	Rationale	Status of Review
**Tyrosinase**	Melanoma							X	**Wistar**: Detection of tyrosinawe transcripts in peripheral blood is associated with clinical stage male sex and site of the primary tumor in melanoma patients. It has prognostic significance with regard to diseaswe free survival in stage III patients	
**VEGF**	Adenocarcinomas<br>Brain<br>Lung<br>Melanoma						X		**UPCI/Gorelik**: VEGF (vascular endothelial growth factor), FGF (fibroblast growth factor) and PDGF (platelet derived growth factor) are the most potent tumor-produced factors involved in tumor-induced angiogenesis.<br><br>Tumor growth largely depends on new blood vessel formation to supply oxygen and nutrients that are essential for tumor cell survival and proliferation. Tumor cells neovascularization is stimulated by various angiogenic factors among which VEGF, FGF and PDGF are most potent. Tumor cells produce large quantity of these factors that induce endothelial cell proliferation, migration and formation of blood vessels. PDGF stimulates periendothelial cells (pericytes, smooth muscle) migration and proliferation that results in blood vessel stabilization. In addition, FGF and PDGF might serve as the autocrine factors for proliferation of various tumor cells (melanoma, breast, prostate cancer). Thus, overexpression of VEGF, FGF and PDGF by tumor cells makes them important tumor markers and targets for cancer therapy. Based on the level of vascularization glioblastoma, melanoma and adenocarcinomas of different organs could be the most suitable malignancies for evaluation.<br><br>The proposed analysis of the angiogenic factors in the blood of cancer patients is based on our hypothesis of tumor-induced cytokine chaos. The Luminex core facility at UPCI provides a possibility to analyze simultaneously 10 or more molecules in the plasma of cancer patients. Therefore the list of angiogenic molecules could be extended to include IL-8, EGF, TNF and IL-6, as well as VEGF, PDGF and FGF.<br><br>**UPCI/Grandis**: Can also be examined in SCCHN. Shown to be a target for EGFR and 3.<br><br>**UPCI/Siegfried**: VEGF is a known angiogenic molecule and is also a gene that is down-stream of the effects of HGF. VEGF gene expression is upregulated by HGF. In another set of studies we showed that high levels of both VEGF and HGF improved the prediction of poor outcome in lung cancer patients.	
**VHL**	REMOVE FROM LIST		X						**REMOVE - FCCC:**<br>[Drosophila vh1]	

ProposedMarker	Organ/Disease Sites	Master Grant	Fox Chase	Kimmel	Penn State	Univ Penn	UPCI	Wistar	Rationale	Status of Review
**Vitamin D Receptor (VDR)**	Breast					**X**			**Univ Penn/DeMichele**: Predictors of response or late effects in breast cancers (Including : IGF-1, ER polymorphisms - PvuII, Xbal, Codon 325 C>G, TA repeat, Vitamin D receptor (VDR) polymorphisms - Taq1, Fokl start codon Collagen I alpha (COLIA1) polys - MSC1, Sp1G>T, IL-6 polys - CA repeat, Nt-634 C>G, -174 G>C TGF-beta polys -509C>T, -869C>T)	
	Prostate					**X**			**Univ Penn/Nathanson**: Genotypes associated with recurrence of prostate cancer in collaboration with Duke Univ. (J. Schildkraut) Inherited Genotypes in androgen metabolisn and insulin-like growth factor pathway (SRD5A2, AR, CYP3A4, CYP3A5, IGFBP3, IGF1, VDR)	
**WT1**	REMOVE FROM LIST		**X**						**REMOVE - FCCC**<br>(Wilms tumor)<br>[Unigene Hs.1145]	
**XPD**	Breast					**X**			**Univ Penn/Weber/Rebbeck**: **Breast** cancer – Candidate low penetrance susceptibility alleles Germline variants genes involved in hormone metabolism, DNA damage response and immune surveillance (CYP3A4, CYP3A5, CYP17, COMT, HSD3B2, CYP19, IL1, IL1R, TNFa,, IL10, IL6, IL12, XPD,XRCC2, XRCC3)	
**XRCC2**	Breast					**X**			**Univ Penn/Weber/Rebbeck**: **Breast** cancer – Candidate low penetrance susceptibility alleles Germline variants genes involved in hormone metabolism, DNA damage response and immune surveillance (CYP3A4, CYP3A5, CYP17, COMT, HSD3B2, CYP19, IL1, IL1R, TNFa,, IL10, IL6, IL12, XPD,XRCC2, XRCC3)	
**XRCC3**	Breast					**X**			**Univ Penn/Weber/Rebbeck**: **Breast** cancer – Candidate low penetrance susceptibility alleles Germline variants genes involved in hormone metabolism, DNA damage response and immune surveillance (CYP3A4, CYP3A5, CYP17, COMT, HSD3B2, CYP19, IL1, IL1R, TNFa,, IL10, IL6, IL12, XPD,XRCC2, XRCC3)	

End of Report
*Total: **163** individual biomarker entries*
Lois Mathews UPCI
Updated 9/15/03
