# Supplementary material for: A Novel Cross-Disciplinary Multi-Institute Approach to Translational Cancer Research: Lessons Learned from Pennsylvania Cancer Alliance Bioinformatics Consortium (PCABC)
Source: Cancer Inform. 2007 Jun 8;3:255–74. (PMC2675833)
Supplement: The Intellectual Property /Technology Transfer Agreements — (additional file #2) [file cin-03-255-s2.pdf]

Additional File #2
Intellectual Property Agreement

INTER-INSTITUTIONAL AGREEMENT FOR INTELLECTUAL PROPERTY MANAGEMENT DEVELOPED UNDER PENNSYLVANIA DEPARTMENT OF HEALTH GRANT (ME-01740) PENNSYLVANIA CANCER BIOINFORMATICS CONSORTIUM

– FOR PCABC USE ONLY –

This Inter-Institutional Agreement (the "Agreement") is made effective as of this ____ day of ______________, 2002, by and among the UNIVERSITY OF PITTSBURGH – OF THE COMMONWEALTH SYSTEM OF HIGHER EDUCATION, FOX CHASE CANCER CENTER, KIMMEL CANCER CENTER OF THOMAS JEFFERSON MEDICAL COLLEGE, THE PENNSYLVANIA STATE UNIVERSITY, and THE WISTAR INSTITUTE (the "Parties").

WHEREAS, each of the parties is receiving money to perform work as defined in Grant ME-01740 (the "Grant") as part of the Commonwealth of Pennsylvania's Commonwealth Universal Research Enhancement Program. Dr. Ronald Herberman of the University of Pittsburgh is serving as Principal Investigator for this Grant. The Parties anticipate that patentable or copyrightable intellectual property will result from their work under the Grant, and hereby agree as follows:

1. If any Invention ("Invention" means any invention or discovery which is or may be patentable or otherwise protectable under Title 35 of the United States Code or which may be copyright protected under Title 17 of the United States Code) results from work by the Parties under the Grant, each and every inventor will disclose such Invention to that inventor's technology transfer office. Each technology transfer office of the Parties will communicate this to each of the other technology transfer offices of the Parties.

2. Ownership of any Invention will be determined by federal patent law or federal copyright law, as applicable.

3. Each and every Invention will be assigned by the inventor to that inventor's institution.

4. If any Invention is assigned to more than one Party, each of the Parties shall negotiate an inter-institutional agreement that will designate which Party shall manage the Invention and be responsible for its patenting and licensing, and for monitoring and maintaining said license. Any royalty income accruing from said license shall be distributed according to the terms established in the inter-institutional agreement.

5. Any Invention solely owned by one Party shall be administered by that Party's institutional intellectual property policy.

6. This Agreement shall be governed in all respects by the laws of the Commonwealth of Pennsylvania. This Agreement may not be amended or modified except by the execution of a written instrument executed by all of the Parties hereto.

7. This Agreement shall be in effect from the date first written above and shall continue for the duration of the Grant.

IN WITNESS WHEREOF, the Parties have caused this Agreement to be executed by their duly authorized representatives.

UNIVERSITY OF PITTSBURGH – OF THE COMMONWEALTH SYSTEM OF HIGHER EDUCATION
By:______________________________
Name: ___________________________
Title: _Executive Vice Chancellor_____

FOX CHASE CANCER CENTER
By:______________________________
Name: ___________________________
Title: ____________________________

KIMMEL CANCER CENTER OF THOMAS JEFFERSON MEDICAL COLLEGE
By:______________________________
Name: ___________________________
Title: ____________________________

PENN STATE CANCER CENTER
By: ______________________________
Name: ___________________________
Title: ____________________________

THE PENNSYLVANIA STATE UNIVERSITY
By:______________________________
Name: ___________________________
Title: ____________________________

THE WISTAR INSTITUTE
By:______________________________
Name: ___________________________
Title: ____________________________
