# Supplementary material for: A Novel Cross-Disciplinary Multi-Institute Approach to Translational Cancer Research: Lessons Learned from Pennsylvania Cancer Alliance Bioinformatics Consortium (PCABC)
Source: Cancer Inform. 2007 Jun 8;3:255–74. (PMC2675833)
Supplement: Biomarker Disclosure Form — (additional file #3) [file cin-03-255-s3.pdf]

Additional File #3
Biomarker Disclosure Form

Pennsylvania Cancer Alliance Bioinformatics Consortium (PCABC)

(To be completed by investigator submitting proposed priority biomarker to the Consortium)

Biomarker Ownership Disclosure Form

Biomarker Name: ____

Biomarker Description and intended use of biomarker:
____
____
____

1. Please state source of this biomarker:

Developed and solely owned by providing center: ________

or

Obtained from outside source –***if so, please complete section 2*** ________

2. Source of biomarker not owned by provider

Source:
____

Category (check one):

- Public domain (identify source, GeneBank, etc): ____________
- Other university/academic - Obtained MTA ____________
- Other university/academic – No MTA executed ____________
- Commercial third party: ____________
- Unknown original – please research ____________

3. To your knowledge, is this biomarker subject to an exclusive license or option to any third party or are any such agreements being negotiated?

4. Please state any other pre-existing ownership or third party rights you may be aware of surrounding this biomarker, including any restrictions on use imposed by a commercial entity that provided the biomarker:

UNIVERSITY OR INSTITUTION NAME

Principal Investigator
Office of Technology Management

BY:___________________________________(date)

BY:___________________________________(date)

VER05 LM-MM-RBH-MB-EO-TC 120202
