# Supplementary material for: A Novel Cross-Disciplinary Multi-Institute Approach to Translational Cancer Research: Lessons Learned from Pennsylvania Cancer Alliance Bioinformatics Consortium (PCABC)
Source: Cancer Inform. 2007 Jun 8;3:255–74. (PMC2675833)
Supplement: Template IRB Protocol for PCABC project — (additional file #4) [file cin-03-255-s4.pdf]

Additional File #4
APPLICATION FOR EXPEDITED IRB REVIEW

Protocol Title: Pennsylvania Cancer Alliance's "Bio-informatics, Data warehouse and Bio-repository" Project

Principle Investigator: John Doe, MD
Generic Cancer Center
123 Main St.
Hometown, PA 12345
Email: jonhdoe@email.com
Office: (123) 555.1234

Co- Investigators: Jack Doe, MD
Generic Cancer Center
123 Main St.
Hometown, PA 12345
Email: jonhdoe@email.com
Office: (123) 555.1234

Jane Doe, MD
Generic Cancer Center
123 Main St.
Hometown, PA 12345
Email: jonhdoe@email.com
Office: (123) 555.1234

Protocol for Pennsylvania Cancer Alliance's "Bioinformatics, Data warehouse and Bio-repository" Project

1. Objectives and specific aims:

As part of the second year of the Pennsylvania Cancer Alliance Bioinformatics Consortium (PCABC), we have decided to change the title of this group's project from "Bioinformatics Consortium to Enhance Translation and Clinical Research" to "Pennsylvania Cancer Alliance's Bioinformatics, Data warehouse and Bio-repository Project" to truly reflect the Consortium's specific aim to expand our efforts to enhance our information system to include tissue collection and biomarker performance on selected tissues. During 2003, we will focus on prostate, breast and melanoma tissue specimens from the six partner organizations. These institutions include: The University of Pittsburgh Cancer Institute, Kimmel Cancer Center of Thomas Jefferson University, Fox Chase Cancer Center, Abramson Cancer Center of the University of Pennsylvania, The Wistar Institute and the Penn State Cancer Institute. The subject of this year's

proposal is to aggregate tissue specimens at the institution of origin and make them available with clinical annotations through the information system proposed in the attached application. This project was approved by the IRB last year (see Appendix A: *Approved IRB application #020345-0203 for 2002*). The focus of this year's efforts will be to expand the research efforts to include tissue banking of these 3 tissue types and evaluate the utility of the tumor type specific biomarkers from the biomarker list.

In addition to aggregating tissues from these 3 cancer sites, we will be requiring the partnering institutions to collect pathology tumor markers and a variety of clinical outcome data on each of these specimens for central storing in the database system.

In addition to storing the tissue samples with the clinical annotations, we will propose within the Consortium to perform biomarker tests on the list of biomarkers attached below (see Appendix B: *A Compilation of biomarkers suggested by investigators from the PCABC*). These tables are a compilation of biomarkers suggested by investigators from all six centers. The PCABC asked the research community to help the Consortium to determine which markers are of interest strategically to compliment studies already underway or to add to banks of information already accumulated on a particular marker. For each biomarker submission, the PCABC asked the investigator to: 1) name the marker and primary cellular function or pathway involved in its impact on the neoplastic cell, 2) provide a short description of the background work done on this marker and why they feel it would be important or at least worthwhile to pursue it as a therapeutic and/or diagnostic marker, and 3) designate the organ system site they are most interested in seeing this marker explored and the rationale behind their selection. The logic was to ask these well-published premier experts and program leaders from each center to work with the Consortium in identifying biomarkers that have the most promise, with the intention of targeting those biomarkers first and foremost. The table is a "work in progress," as each disease/organ sub-committee gets organized and begins the task of reviewing and prioritizing the biomarkers and rationale.

We do not expect that each organ site and each biomarker will be performed by each institution. Instead, a scientific review group that is assembled as part of the committee structure of the Bioinformatics Consortium will decide which biomarkers are performed on which tumor sites by which partnering organizations as part of this process.

However, to protect each institution, we have carefully studied the biomarkers and their intellectual property (IP) characteristics to make sure the IP protected markers will be performed by the institutions offering them and that there will be agreements between the institutions to protect both the patient's privacy and confidentiality when these biomarker assays are performed. In addition, it is our intention that in subsequent years of the proposal, additional IRB's will be generated on specific use of these tissues across institutions. For example, if a group at the University of Pittsburgh wanted to partner with the University of Pennsylvania in a melanoma project, a separate IRB that would address tissue specimens collected at both institutions and the exact nature of that research that would be performed will be the subject of a subsequent IRB. Hence, the purpose of this proposal is to allow us to bank and share de-identified information

through a central database in the goal of providing this information for investigators across the six institutions that are part of this Consortium.

2. Background and Significance:

One of the clear barriers to research of the type proposed in this IRB is that individual institutions do not have the necessary numbers of specimens and carefully collected clinical outcomes to perform larger scale investigations and clinical trials. The purpose of this Bioinformatics Consortium is to facilitate collaborative research among the six member institutions as well as provide access to other institutions both within and outside of the state eventually.

As part of the rationale that the State of Pennsylvania, Department of Health has asked us to pursue this effort is to provide a local "leg-up" to members of the Pennsylvania community in providing economic stimulus in the form of job creation and training opportunities and new company formation in Pennsylvania. Access to these highly annotated clinical tissue specimens from six leading cancer centers in Pennsylvania acting as a single unit will make considerable strides to facilitate this activity.

3. Research Design and Methods:

The system is designed as a virtual bio-repository that gathers information on banked tissues and patients in research trials – including clinical and molecular (gene and protein data) – from six major Pennsylvania cancer centers: The University of Pittsburgh Cancer Institute, Kimmel Cancer Center of Thomas Jefferson University, Fox Chase Cancer Center, Abramson Cancer Center of the University of Pennsylvania, The Wistar Institute and the Penn State Cancer Institute. This data will be de-identified at the local institutions and made available in a central data warehouse for visualization and query.

The Following Honest Broker System or Process will be utilized: Pathology and Oncology Informatics and Center for Pathology Quality and Healthcare Research: Health Sciences Tissue Bank (HSTB), Clinical Outcomes, and Cancer Registry (UPMC/IRB Honest Broker Approval Number: HB015).

The workflow for entering data into the virtual bio-repository will be as follows:

1. The local (physical) tissue bank will identify cases appropriate for inclusion in the Cancer Alliance's virtual bio-repository (warehouse).
2. The local (physical) tissue bank will pre-process data on these cases. The most important component of pre-processing will be de-identification. All de-identification will occur at the local banks. No identifiable data will be sent to the virtual bio-repository (warehouse).
3. De-identified data will be entered into the warehouse through a web site. The data entry web site uses radio buttons, combo boxes and other highly constrained data elements.

4. The local (physical) banks will label each case with a de-identified number. This number will be used to link the information in the warehouse to the cases in the local banks. The linkage codes will be stored locally, using appropriate electronic and physical safety measures. Only the local banks will have access to these linkage codes.
5. The warehouse will contain very minimal demographic data and will comply with all HIPAA requirements. In particular there will be no:

    - No Patient names
    - No Medical Provider names
    - No Patient Addresses (including Zip Codes, States and Countries)
    - No social security numbers or any other personal Ids.
    - No accession numbers or other medical Ids.
    - No dates (including no date of birth or date of diagnosis).
    - All time periods will be entered as plus or minus months from diagnosis. For example the "date of first recurrence" will not be entered as a date but rather as "months from diagnosis" (i.e. Date of First Recurrence = 67). In local tissue banks will not enter any fixed dates into the virtual bio-repository (warehouse).
    - Currently the system does ask for age at diagnosis. There is a discussion about constraining this by decade (20s, 30, 40s, etc).
    - There will be no free text of comment fields. All data will be entered into highly constrained "synoptic" fields.
    - In summary, no identifiers

6. Access to the data entry application is controlled by user name and password.

Cases entered into the virtual bio-repository will be scanned for logical errors (e.g.: first recurrence before diagnosis etc.).

The workflow for querying the warehouse will be as follows:

Initially, access will be limited to members of the Alliance using a user and password system. The data in the warehouse will eventually be made available to the public using the model of the NCI's Collaborative Prostate Cancer Tissue Resource and the NCI's Collaborative Breast Cancer Tissue Resource. A single individual at each facility will be able to provide user names and passwords for researchers at that institution.

Significantly, access to the data will be through highly constrained "click and point" interface. There will be no mechanism for ad hoc query. The output of the system will therefore be highly constrained.

Should a researcher find tissue samples that may be useful in ongoing or proposed research, the researcher will have to contact the Cancer Alliance and the local bank(s) that hold the tissue. Tissue will not be released until after:

- IRB approval at the researcher's institution – for patient safety
- IRB approval at the tissue bank institution – for patient safely
- Approval of the Cancer Alliance's Scientific Review Committee – to determine if the proposed research has validity and justifies the use of potentially valuable tissue resources.
- Approval of the local (physical) tissue bank - As "owner" and guardian of the tissue specimen (the local banks may have their own review committees)

In addition, an *Executive Group* of PCABC institute leaders was established initially to oversee and guide Consortium activities. Several sub-committees report to the Executive Group. These include:

Organ/Disease Site Sub-committees

During this phase of the project sub-committees have been organized for three priority disease/organ sites - breast, melanoma and prostate with the intention of expanding to other disease/organ sites later in the study (lung, colorectal, brain, etc). Each sub-committee is comprised of a clinician or researcher from each of the six centers specializing in the treatment and research of that particular disease/organ. The charge of each sub-committee is to review the list of proposed priority biomarkers (gathered over the past year from all centers as valuable targets for further investigation), and determine: a) Is the list complete? b) What markers should be a priority? c) What assays can each center perform? d) Take inventory of current clinical trials and specimens available at each center for their respective organ/disease e) Review the preliminary Common Data Elements modules developed to date f) Review the preliminary findings of the IRB Sub-Committee to date g) Work with other sub-committees to develop strategies for moving forward (re: maintaining quality control of specimens, volume of specimens, data collection format, data storage, collection and transfer of tissues, patient consent regulations, etc.).

IP/Tech Transfer Sub-committee

This group has the task of reviewing the issues surrounding how to deal with any intellectual property that develops as a result of the Consortium's efforts. This group is identifying how the legislative language that dictates the grant award (Tobacco Settlement Act, Bayh-Dole Act, etc.) affects intellectual property (IP). The committee will begin to define potential IP and has devised a framework inter-institutional agreement to deal with the technology transfer needs of the Consortium (See attached document, approved by all centers and currently in process of obtaining signatures). This committee includes general counsel, business development directors, technology transfer directors, and faculty with experience in this important and complex area.

IRB Sub-Committee

This sub-committee had the initial task of: a) reviewing current practices within each center regarding sharing of banked tissue, b) comparing current patient informed consent samples, and begin developing standardized patient consent forms that all centers will use when collecting samples for analysis by the Consortium, and c) communicating with each center's tissue banks to determine what tissue samples are sharable now, how many, etc.

The PCABC is fortunate to have on this committee an IRB chairman, a HIPAA regulatory specialist and a tissue bank director who can help direct the dialogue and provide thorough guidance to the Executive Group.

Common Data Elements (CDE) Sub-Committee
This important sub-committee was one of the first to be organized in order to decide what information on specimens and assays will be tracked. Standardization and compatibility will be paramount to successful data sharing. To that end, the sub-committee has broken down the task into 9 modules: Patient demographics, family history, clinical history, genomic information, patient consent issues, pathology report, tissue sample descriptors, outcomes and biomarkers. The group first reviewed the NCI and NCICB CDE's (with the possibility of interfacing with these databases at some point). The goal of this component of the PCABC is to enable the rapid implementation of a tissue and data repository for the Consortium in support of the biomarkers focus. The intent of this modularization is to enable generalizable CDE's to be commonly developed across tissues/organs/cancers and to facilitate the swapping in/out of specialized modules as they are needed. The group will concentrate of developing a CDE model for breast samples, and replicate that model uniformly for all other organ/disease sites (melanoma, prostate, lung, colorectal, etc.) This committee is comprised of the PCABC's Working Group infrastructure of molecular pathologists, microbiologists, oncology informatics pathologists, genomics core directors, and others who bring a wealth of experience in collaborative data collection.

In summary, the Pennsylvania Cancer Alliances Bio-informatics, Data warehouse and Bio-repository project is seeking to create a central resource through which researchers can find tissue samples. The resource will have no access at any time to patient identified data and tissue will not be made available to researchers without IRB, Scientific Review Committee approval and local control.

4. Human subjects

In addition to being an information- sharing project, we will now store tissues in repositories located at each institution. We will prospectively consent using our universal tissue banking consent form (see Appendix C: The universal consent form for tissue banking of excess tissues IRB #981252) which will allow us access to serum and tissue specimens any prior or future specimens on the patient's consented in these three priority organ sites (prostate, breast and melanoma tissue specimens) at all six institutions. We have had this universal banking consent in place at the University of Pittsburgh Medical Center, as well as at several other institutions nationally which we have shared our protocols for universal consent tissue banking. The subject population will consist of both males and females. The age range of the subjects will be 18-99 years. The racial and ethnic characteristics of this group reflects the demographics of the State of Pennsylvania and the surrounding area and/or the patient population of the Cancer Centers' part of the PCABC. We shall attempt to recruit patients in respective proportion to these demographics. No exclusion criteria shall be based on race, ethnicity, or HIV status.

a. **<u>Inclusion of Children</u>**
   <u>This study will not include children.</u>

b. **<u>Recruitment procedures</u>**
   There is no active recruitment of subjects for this study. Biological materials collected for this project will be prospectively consented as mentioned above using the IRB approved protocol #981252.

c. **<u>Risk/ Benefit Ratio</u>**
   <u>There will be no risks for the patient.</u> This study will not require anything in addition to what is already being done as standard of care.

   The study will help improve the overall understanding of prostate cancer, breast cancer, and melanoma. **It will not provide any direct benefit to the patient.**

5. **<u>Costs and Payments</u>**
   There will be no costs or payments associated with this study.

6. **<u>Data Safety and monitoring Plan</u>**

A data and safety and monitoring plan will be implemented by the Principal Investigator to ensure that there are no changes in the risk/benefit ratio during the course of the study and that confidentiality of research data is maintained. Each member of the study team will meet with the PI and review confidentiality issues and complete a confidentiality agreement, prior to having contact with research subjects. Investigators and study personnel will meet monthly to discuss the study (e.g., study goals and modifications of those goals; subject recruitment and retention; progress in data coding and analysis; documentation, identification of adverse events or research subject complaints; violations of confidentiality) and address any issues or concerns at that time. Minutes will be kept for these meetings and will be maintained in the study regulatory binder. Any instances of adverse events will be reported immediately the University of Pittsburgh IRB using the standard forms and/or procedures that have been established by the IRB. The yearly IRB renewal for this study will include a summary report of the Data and Safety Monitoring Plan findings from the prior year.

We believe this project entails no risks to UPMC patients. We believe that further sharing of de-identified data, as outlined in detail in section 3 of this protocol, between centers significantly enhances the quality and quantity of cancer research performed in Pennsylvania as part of our mandate from the State Department of Health Tobacco settlement program. We are, therefore, applying for an expedited IRB-approval of the following project proposal so that members of the University of Pittsburgh IRB understand the current and future directions of this exciting proposal.

7. Investigators Qualifications:

a. <Insert qualifications on John Doe, MD>

b. <Insert qualifications on Jack Doe, MD>

c. <Insert qualifications on Jane Doe, MD>
