# Supplementary material for: A Novel Cross-Disciplinary Multi-Institute Approach to Translational Cancer Research: Lessons Learned from Pennsylvania Cancer Alliance Bioinformatics Consortium (PCABC)
Source: Cancer Inform. 2007 Jun 8;3:255–74. (PMC2675833)
Supplement: Template IRB Protocol for the Honest Broker system — (additional file #5) [file cin-03-255-s5.pdf]

Additional File #5

UPMC Health System/University of Pittsburgh Institutional Review Board

APPLICATION for the CERTIFICATION OF HONEST BROKER SYSTEMS/PROCESSES
(Refer to UPMC Health System Policy: HS; Index Title: HIPAA; Subject: Honest Broker Certification Process for the De-Identification of Research Data)

1. Specify the School, Department, Division, or Center for which this Honest Broker System/Process is being developed:

This Honest Broker System has been developed for collaborations between the University of Pittsburgh Department of Biomedical Informatics (Director: Michael Becich, M.D., Ph.D.), the Center for Pathology Quality and Healthcare Research (Director: Stephen S. Raab, M.D.), the Health Sciences Tissue Bank (HSTB), Department of Pathology Clinical Outcomes and the UPMC Network Cancer Registry to support faculty, staff, and graduate students who perform research within these inter-related groups. The Director of the UPMC Health Sciences Tissue Bank is Rajiv Dhir, M.D. and the Director of the UPMC Network Cancer Registry is Sharon Winters, M.S., RHIA, CTR.

2. Specify the individual who will assume responsibility for the appropriate management and oversight of this Honest Broker System/Process:

Name: **Sharon Winters**
Title: Director, Registry Information Services, UPMC Network Cancer Registry
Address: UPMC Cancer Pavilion, 3rd floor
Telephone Number: 412-647-6390
FAX Number: 412-647-5380
E-mail Address: winterssb@msx.upmc.edu

3. Specify the names of all additional individuals who will be involved in performing honest broker services under this Honest Broker System/Process:

HSTB:

1. **Rajiv Dhir, M.D.**
2. **Michelle Bisceglia**
3. **Aprell Delo**
4. **Lindsay Mock**
5. **Ashokkumar A. Patel, MD**
6. **Fang He, MS**
7. **Amelia Hensler**
8. **Kelly Santo**
9. **Patricia Clark**
10. **Linda Lloyd**
11. **Sambit Mohanty, MD**
12. **Elizabeth Ritchey**

13. Regina Skwortz
14. Kristin Valchar

Clinical Outcomes:
1. **Jennifer Condel**
2. **Colleen Vrbin, B.S.**
3. **Brian Turcsanyi**
4. **Erica Satifka**
5. **Stephen Bruno**
6. **Beverly Sutkowski**
7. **Laura Mahood**

Cancer Registry:
1. **Sharon Winters, M.S., RHIA, CTR**
2. **Jennifer Ridge-Hetrick, B.S., CTR**
3. **Louise Mazur, RHIT, CTR**
4. **Lorraine Ickes**
5. **Heidi Patterson-Orlando, CCRP**
6. **Brenda Crocker**
7. **Roberta Elnyczky**
8. **Denine Maglicco**
9. **Julia Michel**
10. **Rick Nestler**

4. HIPAA Complete (i.e., "Safe Harbor") De-Identification of Medical Record Information:

a. For <u>electronic</u> medical record information, address the processes and/or systems that will be used to fully de-identify (i.e., HIPAA "Safe Harbor" compliant) the information for subsequent use by your affiliated researchers. (Note: See Attachment A for HIPAA "Safe Harbor" de-identification requirements.)

The Co-Path Laboratory Information System (LIS) is used by the Department of Pathology for managing its clinical information. This LIS has the capability to generate de-identified reports, as per the needs of Safe Harbor compliance.

The Cancer Registry has information in its archives that will be needed for research use. The Registry provides this information, de-identified per Safe Harbor requirements, using electronic as well as manual tools.

Clinical Outcomes projects also currently involve abstraction of clinical information from the UPMC system clinical database, MARS. This data abstraction takes place manually, using hard-copy data collection forms to record information abstracted from these electronic medical records. IRB-approved hard-copy data collection sheets have patient identifiers on the top of them (medical record numbers), in order to gain access to the appropriate medical records. All recorded data points on the hard copy data collection sheets below the identifiers are data satisfying the HIPAA definition of a

fully de-identified data set. After complete recording of all data on the hard copy data collection sheets, the honest broker de-identifies them by physically tearing off and destroying the top portion of the sheets. These de-identified data sheets are then transferred to project data entry personnel or to project investigators for project use.

b. For paper-based medical record information, address the processes and/or systems that will be used to fully de-identify (i.e., HIPAA "Safe Harbor" compliant) the information for subsequent use by your affiliated researchers. (Note: See Attachment A for HIPAA "Safe Harbor" de-identification requirements.)

*The generation of "Safe Harbor" compliant data also uses manual means. The **HSTB** has been utilizing a system of linkage codes for specimens and annotating data. The linkage codes are retained by the **HSTB** on a password protected secure computer. Any printed records of these linkage codes are stored in a secure, locked filing space. The use of paper recording is strongly discouraged. The predominant mechanism used is electronic files on secure computers. Any excess paper records generated are destroyed using paper shredders and secure University/ UPMC contracted disposal contractors.*

The Outcomes group also has a similar workflow process and a system for de-identifying information abstracted from paper-based medical record information exactly as described above for electronic information. Any paper trail generated is either stored in a secure, locked filing system (if needed) or destroyed via shredding and secure disposal.

5. Limited Data Sets of Medical Record Information:

a. For electronic medical record information, address the processes and/or systems that will be used to develop Limited Data Sets of the information for subsequent use by your affiliated researchers. (Note: See Attachment A for HIPAA Limited Data Set requirements.)

The process followed for generation of "limited data sets" will be a combination of electronic and manual methods. The electronic systems are coded to generate information that follows the "Safe Harbor regulations". Some of these systems have the capability for extracting additional information according to specified additional data fields. Since the "Limited Data set" requirements contain some additional data elements, as compared to the "Safe Harbor" requirements, these elements will be extracted, and provided, electronically, where possible. In addition there will be instances when this might not be possible. Manual methods, as described above, will be used. Any additional information to be provided will be manually extracted and added to the "Safe Harbor" compliant documents. The records of this additional information needed will be placed in secure, locked filing cabinets.

The above protocol will apply to all the three groups covered in this submission,

namely the ***HSTB****,* ***Clinical Outcomes****, and the* ***Cancer Registry****.*

b. For paper-based medical record information, address the processes and/or systems that will be used to develop Limited Data Sets of the information for subsequent use by your affiliated researchers. (Note: See Attachment A for HIPAA Limited Data Set requirements.)

The generation of "Limited Data Sets" from paper-based medical record information will be similar to that for the fully de-identified data described above. The generation of "Safe Harbor" compliant data also uses manual means. The ***HSTB*** *has been utilizing a system of linkage codes for specimens and annotating data. The linkage codes are retained by the* ***HSTB*** *on a password protected secure computer. Any printed records of these linkage codes are stored in a secure, locked filing space. The use of paper recording is strongly discouraged. The predominant mechanism used is electronic files on secure computers. Any excess paper records generated are destroyed using paper shredders and secure University/ UPMC contracted disposal contractors.*

The Outcomes group also has a similar workflow process and a system for de-identifying information abstracted from paper-based medical record information exactly as described above for electronic information. Any paper trail generated is either stored in a secure, locked filing system (if needed) or destroyed via shredding and secure disposal.

These protocols will apply to all the three entities covered in this submission, namely the ***HSTB, Clinical Outcomes,*** *and the* ***Cancer Registry****.*

c. Address your policies, procedures and controls for ensuring that Limited Data Sets of medical record information that you proved to your affiliated researchers contain only the minimum necessary information needed to perform the research. (Note: These policies should include statements specifying that the medical record information provided to researchers under a Limited Data Set will be consistent with the specific data elements requested in the corresponding IRB-approved research application and Data Use Agreement.)

The policies and procedures in place currently focus on the following major issues:

1. ***IRB approval:*** *The three entities covered in this submission, namely the* ***HSTB****,* ***Clinical Outcomes****, and the* ***Cancer Registry****, ask the researcher to submit to them a copy of the* ***IRB submission and the IRB approval****. This ensures that appropriate institutional approvals have been obtained. The IRB submission and approval processes guide us to utilize the minimum information needed for the research project. The IRB submission also provides specific details regarding the data elements requested in the research IRB submission. Thus, the medical record information provided to researchers under a Limited Data Set will be consistent*

with the specific data elements requested in the corresponding IRB-approved research application and Data Use Agreement.

2. ***De-identification**: The biological materials, if needed, and the annotating information, are provided to the researchers totally devoid of any identifiers. Linkage codes are used as appropriate and the coding information is stored on password-protected computers. Any paper trail generated is stored in secure, locked filing cabinets. It is the responsibility of the supervisors of the laboratory and data entry and retrieval personnel to ensure that the protocols are being followed. They are encouraged to seek help from the faculty in charge of these facilities and seek guidance and help. In addition there are weekly meetings to discuss any potential issues.*

3. ***Oversight and training**: A quarterly review (once every three months) of the protocols and procedures will be performed by the manager of this Honest Broker System (Sharon Winters) and compliance by the three groups will be documented. Currently, the supervisor of the **HSTB** (Michelle Bisceglia) meets with the staff of the **HSTB** and goes over the electronic and paper trail of requests generated and fulfilled over the last quarter. Any issues or questions pertaining to the workflow over the last quarter are discussed.*

   In addition there are periodic review sessions where protocols and policies, and possible issues and clarifications, are discussed. The faculty members on this submission periodically present on issues of importance and interest (informal didactic teaching). These sessions focus primarily on issues pertaining to IRB, confidentiality and advances in techniques and methods.

6. Assignment of Re-Identification Codes to De-Identified (HIPAA "Safe Harbor") Medical Record Information and Limited Data Sets:

Address your policies, procedures and controls for the assignment of re-identification codes to the de-identified (HIPAA "Safe Harbor") medical record information and/or Limited Data Sets of medical record information provided to your affiliated researchers. (Note: These policies should include statements specifying that the assignment of re-identification codes will be based on project-by-project verification that the IRB granted approval of the use of re-identification codes. In addition, include statements addressing how re-identification codes will be appropriately managed by the honest broker so as to prevent researcher access to information linking these codes with corresponding patient-subject identifiers.)

*The de-identified information is a subset of the data available in the repositories of the **HSTB** or the **Cancer Registry** or a subset of the data available in Pathology LIS or UPMC clinical databases. The **HSTB, Clinical Outcomes**, or the **Cancer Registry** groups will generate the "Safe Harbor" and limited data sets of information from the already existing information, as described above. Linkage codes will be used, as*

appropriate, to enable the tissue and data repositories to access further information pertaining to the individuals in the studies. This process will be initiated only when it is mandated by the IRB and is part of the initial IRB submission, or any modification thereof.

It will be the responsibility of the manager of this Honest Broker System (Sharon Winters) and the supervisors of both the laboratory and data entry and retrieval personnel to ensure that the protocols are being followed. They will be encouraged to seek help from the faculty in charge of these facilities and seek guidance and help. In addition there will be weekly meetings to discuss any potential issues. There will also be periodic assessment by the manager of this Honest Broker System (Sharon Winters) of the system in place by evaluating the records in place and the problems encountered. These overview sessions will be performed every quarter (once every three months).

7. Documentation and Quality Assurance:

a. Address your policies, procedures and controls for ensuring that Institutional Review Board approval has been granted for the use of de-identified (HIPAA "Safe Harbor) medical record information or a Limited Data Set of medical record information <u>prior</u> to providing such to your affiliated researchers.

*The three entities part of this submission, namely the **HSTB**, **Clinical Outcomes,** and the **Cancer Registry**, will not provide access to any information to any researcher till IRB submission and approval documentation is provided. **No requests are entertained without prior IRB approval.** The three facilities make no exceptions on this issue and do not provide any information prior to IRB approval.*

As part of the oversight process, a quarterly review by the manager of this Honest Broker System (Sharon Winters) will be performed to evaluate the requests fulfilled over the last quarter. This exercise will focus on the information provided and make sure that this was consistent with the IRB submission and approval. This exercise will also assess the temporal workflow and make sure that information was released only after the appropriate approvals were in place. Any discrepancies and errors will be initially locally evaluated and corrective measures taken, including teaching and training. The IRB will also be informed of any errors.

b. Address your policies and procedures for documenting each honest broker transaction with your affiliated researchers (e.g., documentation of the identity of researcher, identity of the research study, the nature of the information provided, corresponding IRB approval information, etc.).

*The workflow plan for all three groups envisages storing information pertaining to the "Honest Broker" system. **HSTB** and **Cancer Registry** requests for information are logged both electronically and via a paper trail. The electronic trail is generated if the request is submitted electronically. Otherwise the date time, nature of request and the*

requesting researcher are documented electronically, if the request is a paper request (request form hard copy printed out and filled). This logging in process also documents the identity of the researcher. The technician involved in this logging process has to contact the supervisor to make sure that the requesting individual is recognized and accepted as part of the University of Pittsburgh community. In instances where the identity is in doubt or if the request is from a researcher outside the University of Pittsburgh, the faculty supervisor is responsible for making contact with the researcher, documenting the contact and communicating the decision back to the technician (generally via E-mail). The technician stores this information and proceeds accordingly.

The process of collecting the information starts only after the researcher provides the IRB submission and approval. *This allows documentation of the magnitude of the approval provided to the researcher.* ***In no instance will the information gathering process be allowed to start if the requesting individual does not have appropriate IRB mandates.***

The next step in this workflow is to document the date and time the information, and biological material (if any), is provided to the researcher. If the transmission of data is electronic (probably in all cases), there will be an electronic trail of transmission of information. If the transmission of information is via hard copy, the researcher, or their designee, will need to sign a form certifying receipt of the information.

Information provided to the **Clinical Outcomes** *group will be collected from Pathology LIS and hospital clinical databases* ***only after the researcher provides the IRB submission and approval.*** *This allows documentation of the magnitude of the approval provided to the researcher.* ***In no instance will the information gathering process be allowed to start if the requesting individual does not have appropriate IRB mandates.*** *The date and time of data collection as well as the date and time of transference of information to the researcher or their designee will be documented by the Honest Broker collecting the data.*

c. Address your policies and procedures for routine monitoring (auditing) of de-identified (HIPAA “Safe Harbor”) medical record information and Limited Data Sets of medical record information provided to affiliated researchers so as to ensure that this information has been de-identified in compliance with respective HIPAA requirements.

We have designed software tools that will allow electronic de-identification, per the needs of HIPAA. The technicians will still do a visual check of the data generated on all requests to make sure that the information provided is consistent with the requirements of “Safe Harbor” and “Limited Data Set” requirement, as appropriate for the request.

The manager of this Honest Broker System also will perform quarterly (every three months) reviews of the requests fulfilled in the preceding quarter.

This policy will be revisited after six months, depending on the efficacy of the electronic system and the time spent on these reviews.

d. Address your policies and procedures for managing and ensuring the security of all identifiable medical record information that is in the Honest Broker's possession during the performance of its de-identification (HIPAA "Safe Harbor") or creation of Limited Data Set functions.

The data is stored electronically on current state-of-the-art databases. The process of creation of "Safe Harbor" is primarily electronic. The "Limited Data Sets" will contain additional data elements that will be added after the "Safe Harbor" data set is created.

We will be making some modifications to our software tools to enable automatic generation of "Limited Data Sets" in a manner analogous to what is in place for the "Safe Harbor" data sets. This method would make the whole process electronic.

The access to the electronic systems is password protected. The logging on to these systems is monitored. The staff will be all trained and be certified honest brokers. They will be aware of their responsibilities in keeping passwords secure and contacting the appropriate personnel in case they think their passwords has been compromised or if there is any other breach of confidentiality

In some instances there might be a need to create a paper hard copy. These hard copies will be stored in locked, secure filing cabinets located in safe areas in the UPMC Cancer Pavilion, 3rd floor, Cancer Center, or Shadyside Medical Building.

8. Business Associate Agreement: Attach to this Application a completed UPMC Business Associate Agreement. (Note: the standard UPMC Business Associate Agreement can be found at http://purchasing.upmc.com).

****

CERTIFICATION OF HONEST BROKER RESPONSIBILITIES

By signing below I agree/certify that:

1. I am cognizant of and will comply with the Federal Policy (Common Rule) and HIPAA regulations and the IRB and UPMC policies governing research involving the use of

identifiable medical record information.

2. I have reviewed this Honest Broker System/Process application in its entirety and I am fully aware of and in agreement with all submitted statements.
3. I will ensure that the Honest Broker System/Processes will be implemented and followed in strict accordance with this application.
4. I will request and obtain IRB and UPMC Privacy Officer approval for any proposed modifications to this application prior to implementing such modifications.
5. I will ensure that all individuals involved in providing the Honest Broker System/Process services are provided with a copy of this current version of this application.
6. I and/or my Honest Broker staff will not provide identifiable medical record information, de-identified medical record information, or Limited Data Sets of medical record information to affiliate researchers until evidence of IRB approval of the corresponding research study is provided.
7. I will respond promptly to all requests for information or materials solicited by the UPMCHS Privacy Officer or the IRB.
8. I will maintain adequate documentation of all Honest Broker transactions with affiliated researchers.
9. I and/or my Honest Broker staff will, under no circumstances, provide the researchers with information that would permit de-identified (HIPAA “Safe Harbor”) medical record information or Limited Data Sets of medical record information to be linked to patient identifiers.
10. I and/or my Honest Broker staff will not intervene or interact with patients in the conduct of Honest Broker functions.
11. I and/or my Honest Broker staff will maintain complete confidentiality of identifiable medical record information in our possession during the performance of Honest Broker functions.

Signature of Individual Responsible for Honest Broker System/Processes	Date

****

Honest Broker System/Process Application Approved:

UPMC HS Privacy Officer	Date
IRB Chair/Vice Chair	Date

ATTACHMENT A

APPLICATION for the CERTIFICATION OF HONEST BROKER SYSTEMS/PROCESSES

A. HIPAA "Safe Harbor" De-Identification of Medical Record Information

HIPAA requires that each of the following identifiers of the individual or of relatives, employers, or household members of the individual must be removed from medical record information in order for the records to be considered de- identified (HIPAA "Safe Harbor")

1. Names
2. All geographic subdivisions smaller than a State, including street address, city, county, precinct, zip code, and their equivalent geocodes, except for the initial 3 digits of a zip code if, according to the currently publicly available data from the Bureau of Census:
    a. The geographic unit formed by combining all zip codes with the same 3 initial digits contains more than 20,000 people; and
    b. The initial 3 digits of a zip code for all such geographic units containing 20,000 or fewer people is changes to 000.
3. All elements of dates (except year) for dates directly related to an individual, including birth date, admission date, discharge date, date of death; and all ages over 89 and all elements of dates (including year) indicative of such age, except that such ages and elements may be aggregated into a single category of age 90 or older.
4. Telephone numbers
5. FAX numbers
6. Electronic mail addresses
7. Social security numbers
8. Medical record numbers
9. Health plan beneficiary numbers
10. Account numbers
11. Certificate/license numbers
12. Vehicle identifiers and serial numbers; license plate numbers
13. Device identifiers and serial numbers
14. Web Universal Resource Locators (URLs)
15. Internet Protocol (IP) address numbers
16. Biometric identifiers
17. Full face photographic images and any comparable images
18. Any other unique identifying number, characteristic, or code, except a code to permit re-identification of the de-identified data by the Honest Broker.

ATTACHMENT A (continued)

B. Limited Data Sets

For Limited Data Sets, HIPAA requires that each of the following identifiers of the individual or of relatives, employers, or household members of the individual must be removed from medical record information.

1. Names
2. Postal address information, other than town or city, State, and zip code
3. Telephone numbers
4. FAX numbers
5. Electronic mail addresses
6. Social security numbers
7. Medical record numbers
8. Health plan beneficiary numbers
9. Account numbers
10. Certificate/license numbers
11. Vehicle identifiers and serial numbers; license plate numbers
12. Device identifiers and serial numbers
13. Web Universal Resource Locators (URLs)
14. Internet Protocol (IP) address numbers
15. Biometric identifiers
16. Full face photographic images and any comparable images
