# Supplementary material for: A Novel Cross-Disciplinary Multi-Institute Approach to Translational Cancer Research: Lessons Learned from Pennsylvania Cancer Alliance Bioinformatics Consortium (PCABC)
Source: Cancer Inform. 2007 Jun 8;3:255–74. (PMC2675833)
Supplement: Universal Consent Template — (additional file #6) [file cin-03-255-s6.pdf]

Pennsylvania Cancer Alliance Bioinformatics Consortium (PCABC)

Additional File #6: Example of Universal Consent Form for Tissue Banking of Excess Tissues

---

Approval Date: June 28, 2005
Renewal Date: June 27, 2006
University of Pittsburgh
Institutional Review Board
IRB# 0506140

CONSENT TO HAVE A TISSUE SAMPLE OR BIOLOGICAL SPECIMEN INCLUDED IN THE HEALTH SCIENCES TISSUE BANK

TITLE: Banking of Tissue and Biological specimens for Research use by the Health Sciences Tissue Bank

PRINCIPAL INVESTIGATOR: **Rajiv Dhir, MD,** Associate Professor of Pathology, (412) 623-1321

SOURCES OF SUPPORT: **National Institutes of Health/ National Cancer Institute**

DESCRIPTION: Because you are or will be undergoing a surgical or other procedure involving the removal or collection of tissue or biological specimens (e.g., blood, urine, saliva, etc.) for your medical care, you are being asked to give your consent to include a sample of your tissue/biological specimen in the Health Sciences Tissue Bank (HSTB). **If you consent to this request, all testing of your tissue/biological specimen required for your medical care will be completed prior to obtaining the sample for inclusion in the HSTB.**

Samples of tissue/biological specimens placed in the HSTB will be used for research studies. The specific nature of the research studies in which your tissue/biological specimen may be used will vary and are not fully known at this time. If the tissue/biological specimen is being surgically removed or collected because you have a specific disease or condition, it is likely that the research studies in which your tissue/biological specimen will be used will be directed at the same disease or condition. However, it is possible that your tissue/biological sample may be used in research studies directed at other diseases or conditions. **Your tissue is released for research studies only after careful review of the research proposal by oversight committees (e.g. tissue utilization committee/ IRB, as appropriate for the research project).**

Your sample will be stored in the HSTB in such a manner whereby it will be possible for the individuals responsible for the HSTB to connect your identity with your sample. Also, in order to use this sample in an effective manner for research, it is also often necessary that your medical information is available for review. Hence, if you agree to include a sample of your tissue/biological specimen in the HSTB, you also agree to allow individuals responsible for the HSTB to review and collect identifiable information from your medical records. **However, when your tissue/biological specimen and medical information are made available for actual use in research studies, it will be provided to the researchers in such a manner whereby it will not be**

possible for them to connect your identity with the sample or medical information.

Because it will not be possible to connect your identity with your tissue/biological specimen when the sample is being used for research, it will also not be possible to inform you of the results of such research.

If you agree to give a sample of your tissue/biological specimen to the HSTB, it will become the property of the University of Pittsburgh and its use will be under the control of the individuals responsible for the HSTB, who are listed on the first page of this consent form. Your medical information and sample will be stored in the HSTB until such time that the sample is used up or no longer felt to be appropriate for use in research studies. Your decision to provide these specimens and identifiable medical information to the HSTB, or to later withdraw from it, will not affect your current or future medical care at UPMC.

RISKS AND BENEFITS: You will receive no direct benefit by agreeing to include a sample of your tissue/biological specimen in the HSTB. However, the availability of such samples for research use is important to the future development of new treatments. There are no additional risks associated with participation. **No additional amount of your tissue/biological specimen will be removed surgically or collected for the purpose of including a sample in the HSTB.** Rather, the sample of your tissue/biological specimen that we are requesting for inclusion in the HSTB is that which would normally be thrown away after the testing for your medical care is completed. Hence, there are no additional risks if you agree to allow a sample of your tissue/biological specimen to be included in the HSTB.

COSTS and PAYMENTS: There will be no additional costs to you or your insurance company if you agree to include a sample of your tissue/biological specimen in the HSTB. You will not be paid for the inclusion of your sample in the HSTB. Use of your tissue/biological specimen for research may lead, in the future, to new inventions or products. If researchers are able to develop new products from the use of the sample that you donate to the HSTB, **you will not receive any money for this donation.**

CONFIDENTIALITY: To protect your privacy, your name will be removed from the sample of your tissue/biological specimen and medical information while it is stored in the HSTB. Your sample and information will be stored in the HSTB using a code number, and only the individuals responsible for the HSTB will be able to connect this code number with your identity. The information linking this code number with your identity will be kept by these individuals in a secure manner.

As stated above, when your tissue/biological specimen and medical information are made available for actual use in research studies, it will be provided to the researchers in such a manner whereby it will not be possible for them to connect your identity with the sample or medical information. Therefore, your identity will not appear in any articles describing the results of research studies that involved the use of your sample.

RIGHT TO WITHDRAW: You may refuse to allow us to include a sample of your tissue/biological specimen in the HSTB. Such a decision will not affect the current or future care that you receive at this institution or any other benefits for which you might qualify.

If you agree to include a sample of your tissue/biological specimen and medical information in the HSTB, you may withdraw your permission at any time through a written request. Depending on your wishes, we can either destroy any remaining amount of the sample or continue to store your sample in the HSTB, but in a totally anonymous manner (i.e., no one, including the individuals responsible for the HSTB, will be able to connect your name with your sample). Your medical information will also be removed from the HSTB.

It is not possible for us to guarantee that we will be able to destroy any of your samples that may have been previously provided for research use since your identity will not be connected with these samples.

****

VOLUNTARY CONSENT: The above information has been explained to me and all of my questions have been answered. I understand that any future questions I have about the donation of a sample of my tissue/biological specimen to the HSTB will be answered by a qualified individual or by one of the individuals responsible for the HSTB, who are listed on the first page of this form. I also understand that I may always request that my questions be answered by one of the individuals responsible for the HSTB. The Human Research Subject Advocate of the Institutional Review Board, University of Pittsburgh (1-866-212-2668), will answer any questions that I may have about my rights as a research subject.

By signing this form I agree to allow a sample of my tissue/biological specimen to be included in the Tissue Bank for use in research studies directed at any disease or condition and I agree to allow the use and disclosure of my medical record information, as described above.

____ _________________________

Patient/Subject Signature Date

I certify that the nature and purpose, the potential benefits and possible risks associated with the donation of tissue samples/biological specimens to the Tissue Bank have been explained to the above individual and that all questions about this donation have been answered.

____ ______________________________

Signature of individual obtaining consent Date
