# Supplementary material for: A Novel Cross-Disciplinary Multi-Institute Approach to Translational Cancer Research: Lessons Learned from Pennsylvania Cancer Alliance Bioinformatics Consortium (PCABC)
Source: Cancer Inform. 2007 Jun 8;3:255–74. (PMC2675833)
Supplement: Material Transfer Agreement Template — (additional file #7) [file cin-03-255-s7.pdf]

Additional File #7

Pennsylvania Cancer Alliance Bioinformatics Consortium (PCABC)

MATERIAL TRANSFER AGREEMENT

THIS MATERIAL TRANSFER AGREEMENT (the "Agreement") is made and entered into as of the ______ day of January, 2006 (the "Effective Date"), by and between the University of Pittsburgh – Of the Commonwealth System of Higher Education, a non-profit Pennsylvania corporation, having an office at 350 Thackeray Hall, Pittsburgh, PA 15260 (the "UNIVERSITY"), and ______ ("RECIPIENT") having an address at ______.

In response to the RECIPIENT's request for the transfer of _____ ("Materials"), from the lab of Dr. _____, UNIVERSITY is willing to provide such Materials, subject to the following terms and conditions:

1. Upon request for a specific quantity of Materials by RECIPIENT, such Materials shall be provided by UNIVERSITY to RECIPIENT, subject to availability of such Materials. The availability of such Materials shall be determined solely by UNIVERSITY.

2. The Materials, and all unmodified derivatives or progeny of the Materials, remain the property of the UNIVERSITY. The Materials are to be used under your immediate and direct control only for the research project set forth on Exhibit A within the above identified RECIPIENT. The Materials, and any unmodified derivatives or progeny of the Materials, are not to be used: (a) in any product, (b) for the purpose of producing any product, or (c) for providing any service in which a product or service is sold or otherwise made commercially available. No other right or license, patent or otherwise, is granted to RECIPIENT for the use of the Materials as a result of our transmission of them to you.

3. The Materials shall not be sold, distributed or otherwise made available to any other party for any purpose.

4. The Materials shall be used with prudence and appropriate caution in any experimental work since not all of their characteristics are known. THEY ARE PROVIDED WITHOUT WARRANTY OF MERCHANTABILITY OR FITNESS FOR ANY PARTICULAR PURPOSE OR ANY OTHER WARRANTY, EXPRESS OR IMPLIED. UNIVERSITY MAKES NO WARRANTY OR CLAIM THAT THE MATERIALS WILL NOT INFRINGE ANY PATENT, COPYRIGHT, TRADEMARK OR OTHER PROPRIETARY RIGHTS. RECIPIENT agrees to release the UNIVERSITY, its trustees, appointees, employees and agents from any liability in connection with use of the Materials by you. RECIPIENT agrees to defend and indemnify the UNIVERSITY, its trustees, appointees, employees and agents from any and all claims and damages in any way arising from the acquisition, use, storage or disposal of the Materials by RECIPIENT.

5. The Materials will be used in compliance with all applicable statutes and regulations, including NIH guidelines on the use of animals or recombinant DNA. The Materials may not be used for *in vivo* testing in human subjects. Materials derived from human donors may not be transferred with any individual donor identifying information.

6. This Agreement shall terminate one (1) year from the date on which it was signed, unless terminated or extended through prior written agreement of the parties. Either party may terminate this Agreement prior to the expiration of the designated term by giving sixty (60) days written notice to the other. The obligations of Recipient hereunder shall survive termination. Upon termination, the Materials shall be either returned to University or destroyed.

7. Upon the execution of this Agreement, RECIPIENT shall be authorized to receive Materials from UNIVERSITY. Requests for specific quantities shall be processed by UNIVERSITY as Materials are available. A shipping cost recovery fee in the amount of $_____________per______ will be charged at the time of such shipments.

UNIVERSITY OF PITTSBURGH – OF THE COMMONWEALTH SYSTEM OF HIGHER EDUCATION

By____
Director, Office of Research Date

RECIPIENT OFFICIAL

By____
Authorized Individual for the Institution Date
Printed Name: ____
Title: _____

RECIPIENT INVESTIGATOR

By____
Recipient Investigator Date
Printed Name: _____

Exhibit A:

7/06

UNIVERSITY OF PITTSBURGH OFFICE OF RESEARCH (OR)
SUBMISSION FORM FOR THE EVALUATION OF AN
OUTGOING MATERIAL TRANSFER AGREEMENT (MTA)

SUBMIT THIS COMPLETED FORM AND SUPPORTING DOCUMENTS TO THE OFFICE OF RESEARCH WITH ANY MTA FOR UNIVERSITY REVIEW AND SIGNATURE

(SUBMIT BY FAX: OR EMAIL: ORMTA@OFFRES.PITT.EDU)

THE UNIVERSITY OF OFFICE OF RESEARCH WILL BE THE LAST SIGNATURE AFFIXED TO ANY OUTGOING MTA

University Principal Investigator (PI) Name/Title:<br>•	Company/Institution Receiving Materials (Recipient):
PI's Departmental Administrative Contact for MTA Follow-up:<br>Name:<br>Phone/Email:	Company/Institution Contact for MTA:<br>Name:<br>Email:
Recipient is:<br>☐ Nonprofit/ Academic<br>☐ For-profit/ Industry	List *ALL* Material being provided under this MTA:

General:

Was all the Material independently developed by you or under your direction at this University?

☐ Yes
☐ No, explain _____

Does the Material incorporate or is the Material derived from materials obtained from a third party.

☐Yes, explain _____
☐No

Was any of the Material brought to this University from somewhere else.

☐Yes, explain and provide applicable contacts _____

☐No

Are there alternative sources to provide the Material?

☐ Yes ☐ No

Is the Material under disclosure to or patented by the University Office of Technology Management (OTM)

☐ Yes ☐ No

Is any of the Material published or in the public domain?

☐ Yes ☐ No ☐ Not Aware

Is any of the Material subject to any third party restrictions *(list all that apply)*

☐ Sponsored Research Agreement/Government or Other Grant *(list applicable proposal/project number(s))*:

____
____
____

☐ License Agreement/Option *(list Company/companies)*:

____
____

☐ MTA:

____
____

☐ Other
☐ None

Compliance:

Is the Material being sent to a recipient located outside of the United States?

☐Yes, specify country _____
☐No

Is the Material of direct human origin?

☐ Yes, attach the appropriate IRB/CORID letter of approval or exemption and applicable consent. If the letter does not name you, append explanation/approval from the named person

☐ No

Does the Material involve recombinant DNA?

☐ Yes, attach applicable IBC/rDNA letter

☐ No

Is the Material hazardous?

☐ Yes, attach EHS approval letter

☐ No

Is the Material on the federal Select Agent list http://www.cdc.gov/od/sap/docs/salist.pdf?

☐ Yes, attach safety officer approval

☐ No

Note: Shipments crossing state lines or leaving the country may require special shipping or handling permits from the USDA - http://www.aphis.usda.gov/ or CDC - http://www.cdc.gov/od/ohs/biosfty/imprtper.htm

---

To the best of my knowledge, the answers to the questions are true, complete and accurate. I have read the referenced MTA and agree to provide the Materials as outlined. I am a University of Pittsburgh faculty member authorized to oversee the transfer of the materials named above.

Principal Investigator:____ Date:_________________
