# Supplementary material for: A Novel Cross-Disciplinary Multi-Institute Approach to Translational Cancer Research: Lessons Learned from Pennsylvania Cancer Alliance Bioinformatics Consortium (PCABC)
Source: Cancer Inform. 2007 Jun 8;3:255–74. (PMC2675833)
Supplement: Prostate CDEs — (additional files #8) [file cin-03-255-s8.pdf]

Additional File #8

Pennsylvania Cancer Alliance Bioinformatics Consortium (PCABC)

Prostate Common Data Elements

vMarch 2006

Cancer Centers

Cancer Center

Site Name

Definition:

Required: Yes; Enterable Field: No, Radio_Button

Validation Rules:

Value	Value Description
ACC, UPenn	
FCCC	
KCC, TJU	
PSU	
UPCI	
Wistar	
CPCTR-GWU	
CPCTR-HOW	
CPCTR-MCW	
CPCTR-NYU	
CPCTR-VUC	
WSU	
Geisinger	

Case Identification

Case Identification

PA CA Case Number

Definition: This is a de-identified number given by the submitting institution to index all data on a given case.

Required: Yes; Enterable Field: Yes

Validation Rules: None

Data Type: Character; Default Value: No default; Maximum length: 32

CDE version

Definition: Version 2 (1/15/2004 aap) with NAACR elements

Required: Yes; Enterable Field: Yes

Validation Rules:

Data Type: Number; Default Value: No default; Data Range:

CDE version

Definition: Version 2 (1/15/2004 aap) with NAACR elements

Required: Yes; Enterable Field: Yes

Validation Rules:

Data Type: Number; Default Value: No default; Data Range:

Research Consent Elements

Consent Status

Tissue Consent Status

Definition: Is there a valid consent in place for use of this patient's tissue in research?

Required: Yes; Enterable Field: No, Radio_Button

Validation Rules: None

Value	Value Description
Valid	
Not Valid	
Unknown (Default)	

Data Consent Status

Definition: Is there a valid consent in place for use of this patient's data in research?

Required: Yes; Enterable Field: No, Radio_Button

Validation Rules: None

Value	Value Description
Valid	
Not Valid	
Unknown (Default)	

Demographics and History

Base Demographics

Age at Diagnosis

Definition: Age of patient at diagnoses in complete years..

Required: Yes; Enterable Field: Yes

Validation Rules: Calculate by subtracting year of birth from year of diagnostic biopsy; if not available use year of prostatectomy.

Data Type: Number; Default Value: -1; Data Range: 25 - 99

Age Range at Diagnosis

Definition: THIS ELEMENT IS FOR DATA QUERY VIEWER PURPOSE ONLY.

Required: ; Enterable Field: No, Radio_Button

Validation Rules:

Value	Value Description

Age Range at Diagnosis

Definition: THIS ELEMENT IS FOR DATA QUERY VIEWER PURPOSE ONLY.

Required: Yes; Enterable Field: No, Radio_Button

Validation Rules:

Value	Value Description
0-20	
21-30	
31-40	
41-50	
51-60	
61-70	
71-80	
>80	

Race

Definition: Code the patient?s race. Race is coded separately from Spanish/Hispanic Orgin.

Required: Yes; Enterable Field: No, Radio_Button

Validation Rules: Needs to have a positive statement in the medical record to make a statement of race. Discordant documents should result in further record review. If the majority of documentation specifies a specific race then that race should be used. Physician notes (ie. OR reports, History/Physical, etc) should take priority over hospital admissions data. "Other" is a combination of races listed. "Unknown" is not known at accessioning. Hispanic races such as Mexican, Puerto Rican, Cuban, and all Europeans Caucasians are recorded as "Caucasians".

Value	Value Description
Caucasian	

African American	
Asian	
Native American	
Other	
Unknown (Default)	
Test Race	

Hispanic Origin

Definition: Code identifying persons of Spanish or Hispanic origin.

Required: Yes; Enterable Field: No, Radio_Button

Validation Rules: Needs to have a positive statement in the medical records. If not recorded, the ROADS guideline recommends that the tumor registrar may consider the patient of Spanish Origin if only documented by surname only or maiden name and there is no contrary evidence that the person is not Hispanic from the medical record. Some patients can be identified as African American but still have a Hispanic origin. In this case, assign Hispanic origin as yes. No documentation of Hispanic origin then conclude patient is not of Hispanic decent (unknown).

Value	Value Description
Yes	
No	
Unknown (Default)	

Patient Disease History

History of Non-prostate Cancer

Definition: Did this patient have a diagnosis of a non-prostate cancer at the time of PCA diagnosis?

Required: Yes; Enterable Field: No, Radio_Button

Validation Rules: None

Value	Value Description
Yes	
No	
Unknown (Default)	

Family Disease History

Family History of Prostate Cancer

Definition: Does the patient have a family history of prostate cancer?

Required: Yes; Enterable Field: No, Radio_Button

Validation Rules: Code whether the patient has a family member i.e. father, uncle, 1st cousin with a history of prostate cancer. Documentation can be found in the H&P. Unknown values should also include cases with inconsistent or discordant information. If no information is found which positively states some family member has cancer should be listed as unknown.

Value	Value Description
Yes	
No	
Unknown	

Patient Exposures

Patient History of Tobacco Use

Definition: What had been the patient's history of tobacco use at the time of diagnosis?

Required: Yes; Enterable Field: No, Radio_Button

Validation Rules: Yes values will include any smoking history-Past, Present, or Usage but NOS. Unknown values should also include cases with inconsistent information, or patients with discordant smoking histories in the medical records.

Value	Value Description
Yes	
No	
Unknown (Default)	

Progression and Outcomes

Clinical Staging (at diagnosis)

Clinical Staging, AJCC Version

Definition: A code that indicates the edition of the AJCC manual used to stage the tumor. This applies to the manually coded AJCC fields It does not apply t o Derived AJCC T,N,M and AJCC Stage Group fields.

Required: Yes; Enterable Field: No, Radio_Button

Validation Rules: Which edition of the AJCC Manual for Staging of Cancer is used. 5th edition was used prior to Dec 31, 2002. 6th edition was implemented in most labs on Jan 1, 2003.

Value	Value Description
2nd Edition	
3rd Edition	
4th edition	
5th edition (Default)	
6th edition	
Unknown	

T stage, Clinical

Definition: Detailed site-specific codes for the clinical tumor (T) as defined by AJCC and recorded by the physician.

Required: Yes; Enterable Field: No, Radio_Button

Validation Rules: Report T stage at initial diagnosis. If ?T1? ? ?T4?, then Invasive Tumor Present = ?Yes? and Tumor Size, Maximum Diameter (cm) must be > 0.

Value	Value Description
TX	
T0	
T1	
T1a	
T1b	
T1c	
T2	
T2a	
T2b	
T2c	
T3	
T3a	
T3b	
T4	
Unknown	

N Stage, Clinical

Definition: Detailed site-specific codes for the clinical nodes (N) as defined by AJCC and recorded by the physician.

Required: Yes; Enterable Field: No, Radio_Button

Validation Rules: Report N stage at initial diagnosis. If used this should be defined by a CT or MRI scan (usually of the pelvis).If ?NX? , then Nodes Examined = ?00" or ?-1"If ?N0", then Nodes Examined > 0, AND Nodes Positive = ?0".If ?N1", then Nodes Examined > 0, AND Nodes Positive > 0.

Value	Value Description
NX	
N0	
N1	
Unknown	

M Stage, Clinical

Definition: Detailed site-specific codes for the clinical metastases (M) as defined by AJCC and recorded by the physician.

Required: Yes; Enterable Field: No, Radio_Button

Validation Rules: If used this should be defined by either a bone scan (most common), or a

CT/MRI of the chest. This can be a known metastasis only at initial diagnosis. All other metastasis after a diagnosis was made is considered a distant metastatic progression, thus should not be evaluated for the clinical M stage for this data element.

Value	Value Description
MX	
M0	
M1	
M1a	
M1b	
M1c	
Unknown	

cTNM

Definition: THIS ELEMENT IS FOR DATA QUERY VIEWER PURPOSE ONLY.

Required: Yes; Enterable Field: Yes

Validation Rules:

Data Type: Number; Default Value: No default; Data Range:

Pathologic Staging

Pathological Staging, AJCC Version

Definition: A code that indicates the edition of the AJCC manual used to stage the tumor. This applies to the manually coded AJCC fields It does not apply t o Derived AJCC T,N,M and AJCC Stage Group fields.

Required: No; Enterable Field: No, Radio_Button

Validation Rules: Which edition of the AJCC Manual for Staging of Cancer is used. 5th edition was used prior to Dec 31, 2002. 6th edition was implemented in most labs on Jan 1, 2003.

Value	Value Description
2nd Edition	
3rd Edition	
4th Edition	
5th Edition (Default)	
6th Edition	
Unknown	

T-Stage, Pathologic

Definition: Detailed site-specific codes for the pathological tumor (T) as defined by AJCC and recorded by the physician.

Required: No; Enterable Field: No, Radio_Button

Validation Rules: Report T stage at initial diagnosis. If ?T1? ? ?T4?, then Invasive Tumor Present = ?Yes? and Tumor Size, Maximum Diameter (cm) must be > 0.

Value	Value Description
pTX	
pT0	
pT1	
pT1a	
pT1b	
pT1c	
pT2	
pT2a	
pT2b	
pT2c	
pT3	
pT3a	
pT3b	
pT4	
Unknown	

N Stage, Pathologic

Definition: Detailed site-specific codes for the pathological nodes (N) as defined by AJCC and recorded by the physician.

Required: No; Enterable Field: No, Radio_Button

Validation Rules: Report N stage at initial diagnosis. Pathological stage is mandatory for all invasive cancers other than M1. Clinical nodal stage is allowed only for non-invasive cancers. If ?NX? , then Nodes Examined = ?00" or ?-1"If ?N0", then Nodes Examined > 0, AND Nodes Positive = ?0".If ?N1", then Nodes Examined > 0, AND Nodes Positive > 0.

Value	Value Description
pNX	
pN0	
pN1	
Unknown	

M Stage, Pathologic

Definition: Detailed site-specific codes for the pathological metastases (M) as defined by AJCC and recorded by the physician.

Required: No; Enterable Field: No, Radio_Button

Validation Rules: This can be a known metastasis only at initial diagnosis. All other metastasis after a diagnosis was made is considered a distant metastatic progression, thus should not be evaluated for the pathological M stage for this data element.

Value	Value Description
pMX	
pM0	
pM1	
pM1a	
pM1b	
pM1c	
Unknown	

pTNM

Definition: THIS ELEMENT IS FOR DATA QUERY VIEWER PURPOSE ONLY.

Required: Yes; Enterable Field: Yes

Validation Rules:

Data Type: Number; Default Value: No default; Data Range:

Most Recent Followup

Most Recent Follow Up (Months from Diagnosis)

Definition: Date of las contact with the patient, or date of death.

Required: Yes; Enterable Field: Yes

Validation Rules: Record the most recent date on which contact was made with the patient, or it was verified through a registrar, physician, or clinical record that the patient was alive. If the patient has died, then the date in this field remains fixed at the last time the patient was verified to be alive, and no further updating is necessary. The date of death (if known) is then entered into the ?months from diagnosis to death? field.

Data Type: Number; Default Value: -1; Data Range: -1 - 999

Follow Up (Months)

Definition:

Required: Yes; Enterable Field: No, Radio_Button

Validation Rules:

Value	Value Description
<13	
13-24	
25-36	
37-60	
>60	

Vital Status

Vital Status at Most Recent Follow Up

Definition: Vital Status of the patient as of the last date of contact.

Required: Yes; Enterable Field: No, Radio_Button

Validation Rules: If Alive or Unknown, then date of death must be blank and Date of ?most recent follow up? must be completed. If dead, then ?months from diagnosis to death? must

not be blank.

Value	Value Description
Alive	
Dead	
Unknown	

Months from Diagnosis to Death

Definition: Months between diagnosis and death

Required: Yes; Enterable Field: Yes

Validation Rules: The date of death, which may be found from doctor?s office records, other cancer registries, death certificates, last admission, or Social Security Death Index webpage. If the exact date of last contact or death is not available, record an approximate date. If Information is limited to a description, use the following: ?Spring? as April, ?The middle of the year? as July, ?The fall of the year? as October, and ?The winter of?as determined if this means the beginning or the end of the year and use January or December as indicated.

Data Type: Number; Default Value: -1; Data Range: -1 - 999

Post Diagnosis Survival(Months)

Definition:

Required: Yes; Enterable Field: No, Radio_Button

Validation Rules:

Value	Value Description
<13	
13-24	
25-36	
37-60	
>60	

Follow up

Definition:

Required: Yes; Enterable Field: Yes

Validation Rules:

Data Type: Number; Default Value: No default; Data Range:

First Recurrence

Months from Diagnosis to First Recurrence

Definition: Months from diagnosis to first recurrence

Required: Yes; Enterable Field: Yes

Validation Rules: Record date of FIRST recurrence regardless of whether it is a prostate recurrence or non-prostate recurrence. This also includes any biochemical recurrences (i.e. increased PSAs post-prostatectomy).

Data Type: Number; Default Value: -1; Data Range: 1 - 999

Diagnosis To First Recurrence (Months)

Definition:

Required: Yes; Enterable Field: No, Radio_Button

Validation Rules:

Value	Value Description
<13	
13-24	
25-36	
37-60	
>60	

First Recurrence Type

Definition: Code for the type of first recurrence after a period of documented disease-free intermission or remission.

Required: Yes; Enterable Field: No, Combo_Box

Validation Rules: Biochemical recurrences should be coded as ?Recurred-Site unknown?. Distant lymph nodes should be coded as ?Distant?, which are outside the confines of the true pelvis and their involvement constitutes distant metastasis. They can be imaged using ultrasound, computed tomography, magnetic resonance imaging, or lymphangiography, and

include: aortic (para-aortic, periaortic, lumbar), common iliac, inguinal, superficial inguinal (femoral), supraclavicular, cervical, scalene, and retroperitoneal (NOS) nodes.

Value	Value Description
No Known Recurrence	
In situ	
Local	
Regional - NOS	
Regional - Tissue	
Regional - Lymph Nodes	
Distant	
Never Disease Free	
Biochemical Recurrence	
Susp. Of Biochemical Recurrence	
Unknown (Default)	

Recurrence Events

Definition:

Required: Yes; Enterable Field: Yes

Validation Rules:

Data Type: Number; Default Value: No default; Data Range:

Overall Metastatic Progression

First Progression

Definition:Location of distant metastases.

Required: Yes; Enterable Field: No, Combo_Box

Validation Rules: Record the first metastatic site, if an actual tissue site recurrence is detected by radiology imaging, biopsy or surgery.

Value	Value Description
No First Location (Default)	
Peritoneum	
Lung	
Pleura	
Liver	
Bone	
CNS	
Skin	
Distant Lymph Nodes	
Other - Generalized - NOS	
Biochemical	
Unknown	
Bladder	
Regional Lymph Node	

Second Progression

Definition:

Required: Yes; Enterable Field: No, Combo_Box

Validation Rules: Record the second metastatic site, if an actual tissue site recurrence is detected by radiology imaging, biopsy or surgery.

Value	Value Description
No Second Location (Default)	
Peritoneum	
Lung	
Pleura	
Liver	
Bone	
CNS	
Skin	
Distant Lymph Nodes	
Other - Generalized - NOS	

Biochemical	
Unknown	
Bladder	
Regional Lymph Node	

Third Progression

Definition:

Required: Yes; Enterable Field: No, Combo_Box

Validation Rules: Record the third metastatic site, if an actual tissue site recurrence is detected by radiology imaging, biopsy or surgery.

Value	Value Description
No Third Location (Default)	
Peritoneum	
Lung	
Pleura	
Liver	
Bone	
CNS	
Skin	
Distant Lymph Nodes	
Other - Generalized - NOS	
Biochemical	
Unknown	
Bladder	
Regional Lymph Node	

Time Line

Overall TL

Definition: THIS ELEMENT IS FOR DATA QUERY VIEWER PURPOSE ONLY.

Required: Yes; Enterable Field: Yes

Validation Rules:

Data Type: Number; Default Value: No default; Data Range:

Therapy TL

Definition: THIS ELEMENT IS FOR DATA QUERY VIEWER PURPOSE ONLY.

Required: Yes; Enterable Field: Yes

Validation Rules:

Data Type: Number; Default Value: No default; Data Range:

Procedure TL

Definition: THIS ELEMENT IS FOR DATA QUERY VIEWER PURPOSE ONLY.

Required: Yes; Enterable Field: Yes

Validation Rules:

Data Type: Number; Default Value: No default; Data Range:

Marker TL

Definition: THIS ELEMENT IS FOR DATA QUERY VIEWER PURPOSE ONLY.

Required: Yes; Enterable Field: Yes

Validation Rules:

Data Type: Number; Default Value: No default; Data Range:

Experimental Data

MOL RECLASS

Definition:

Required: No; Enterable Field: No, Radio_Button

Validation Rules:

Value	Value Description
Yes	
No (Default)	

TMA1

Definition:

Required: No; Enterable Field: No, Radio_Button

Validation Rules:

Value	Value Description
Yes	
No (Default)	

TMA2

Definition:

Required: No; Enterable Field: No, Radio_Button

Validation Rules:

Value	Value Description
Yes	
No (Default)	

Tissue Accession Data

Date and Procedure Elements

De-identified Accession ID

Definition: De-identified accession ID from submitting institution

Required: Yes; Enterable Field: Yes

Validation Rules: This is a de-identified number, chosen by the submitting institution, to label this ACCESSION or SPECIMEN. De-identified patient numbers and block numbers will be added to their respective fields within the database. The system will append a prefix designating the submitting institution. For example, if one is at PITT and enters 12345 the system will designate the accession as PIT-12345. NOTE: There are 3 de-identified numbers within the database: 1) PA CA Case Number: one per patient 2) De-identified Accession ID: this is a specimen from a patient. You can enter multiple specimens per patient. 3) Block ID: this is the tissue block from a specimen. You can enter multiple blocks per specimen.

Data Type: Character; Default Value: No default; Maximum length: 32

Months Between Diagnosis and Accession

Definition: The time in months between the Diagnosis and the Accession (can be a negative for benign biopsies before the Dx)

Required: Yes; Enterable Field: Yes

Validation Rules: None

Data Type: Number; Default Value: No default; Data Range:

Diagnosis To Accession (Months)

Definition:

Required: Yes; Enterable Field: No, Radio_Button

Validation Rules:

Value	Value Description
<13	
13-24	
25-36	
37-60	
>60	

Procedure Type

Definition: Site-specific codes for the type of surgery to the primary site performed. Records the surgical removal of distant lymph nodes or other tissues/organs beyond primary site.

Required: Yes; Enterable Field: No, Combo_Box

Validation Rules: None

Value	Value Description
Prostatectomy NOS	
Cystoprostatectomy	
Prostate Biopsy NOS	
TURP - Transurethrual Resection	
Prostate Aspiration	
Regional Node Exploration Only	

Exploration for Metastases Only	
Blood or Fluid Only	
Other	
Unknown (Default)	
Donor Prostectomy	
Supra Pubic Prostectomy	
Radical Prostectomy	
Retropubic Prostectomy	
Nerve Sparing Radical	
Needle Biopsy	

Invasive Tumor Present

Definition: Was invasive tumor identified in the accession?

Required: Yes; Enterable Field: No, Radio_Button

Validation Rules: If this specimen contains any invasive tumor, one should enter "Yes". If there is no tumor is present (invasive or in situ) one should enter "No". Should the accession show in situ (High Grade PIN) without invasive tumor, use the option "In situ Only". Cases of benign prostate tissue or control/donor prostate tissue, without tumor, should be entered as "No". The "Not Applicable option is used of accessions such as blood samples only.

Value	Value Description
Yes	
No	
No - In Situ Only	
Not Applicable	
Unknown	

Primary or Metastatic Tumor

Definition: Was primary or metastatic tumor identified in the accession?

Required: Yes; Enterable Field: No, Radio_Button

Validation Rules: If the accession contains only the primary prostate tumor tissue, enter "primary". If the accession contains only metastatic prostate tissue, enter "metastatic". Should the accession contain both primary and metastatic prostate tissue samples (for example, a primary site with region mets) enter "both". The "Not Applicable option is used of accessions such as blood samples only.

Value	Value Description
Primary	
Metastatic	
Both	
Neither	
Not Applicable (Default)	
Unknown	

Procedure Event

Definition:

Required: Yes; Enterable Field: No, Radio_Button

Validation Rules:

Value	Value Description

Diagnosis and Grade

Primary Histology

Definition: Codes for the histological type of the tumor being reported using ICD-O-3.

Required: Yes; Enterable Field: No, Combo_Box

Validation Rules: Choose the primary histology in the current accession. Note that some rare types of prostate cancer are not listed and should be entered as "Other". Specimens with tissue, but the histology is not known at the time when accessioning this, then enter ?Unknown?. The option "Not Applicable" is used for cases in which there is no histology, such as accessions that represent a blood sample only.

Value	Value Description

Adenocarcinoma NOS (Default)	
Ductal Adenocarcinoma	
Benign Tissue Only	
In Situ Neoplasia Only	
Mucinous Adenocarcinoma	
Signet Ring Adenocarcinoma	
Basal Cell Adenocarcinoma	
Undiff Non-Small Cell Carcinoma	
Sarcomatoid Carcinoma	
Small Cell Anaplastic Carcinoma	
Squamous or Adenosquamous Carci	
Mesenchymal Tumor NOS	
Lymphoma	
Neuroendocrine Carcinoma	
Transitional Carcinoma	
Other	
N/A -- For Blood fluids	
Unknown	

Gleason Grade, Primary

Definition: Primary Gleason Grade
Required: Yes; Enterable Field: No, Radio_Button
Validation Rules: None

Value	Value Description
1	
2	
3	
4	
5	
NA (Default)	
Unknown	

Gleason Grade, Secondary

Definition: Secondary Gleason Grade
Required: Yes; Enterable Field: No, Radio_Button
Validation Rules: None

Value	Value Description
1	
2	
3	
4	
5	
NA (Default)	
Unknown	

Gleason Sum Score

Definition: Gleason Sum Score
Required: Yes; Enterable Field: No, Radio_Button
Validation Rules: None

Value	Value Description
2	
3	
4	
5	
6	
7	
8	
9	
10	

NA	

Percent Gleason 4/5 Grade

Definition: Percent of the tumor showing Gleason 4 and/or 5 histology. Only used for Gleason 6 and 7 tumors.
Required: Yes; Enterable Field: Yes
Validation Rules: None
Data Type: Number; Default Value: -1; Data Range: -1 - 100

Tumor Differentiation

Definition: Code for the grade or degree of differentiation of the reportable tumor.
Required: Yes; Enterable Field: No, Radio_Button
Validation Rules: None

Value	Value Description
Well	
Moderate	
Poor	
Undifferentiated	
Unknown/Not Applicable (Default)	

Gleason4/5

Definition:
Required: Yes; Enterable Field: No, Radio_Button
Validation Rules:

Value	Value Description
<10%	
11-25%	
>25%	

Gleason Score Equation

Definition:
Required: Yes; Enterable Field: Yes
Validation Rules:
Data Type: Number; Default Value: No default; Data Range:

Tumor Size and Extent

Tumor Size, Maximum Diameter (cm)

Definition: Largest dimension of largest tumor nodule in the specimen in cm
Required: Yes; Enterable Field: Yes
Validation Rules: None
Data Type: Number; Default Value: -1; Data Range: -1 - 5.00

Tumor Size (cm)

Definition: Largest dimension of largest tumor nodule in the specimen in cm
Required: Yes; Enterable Field: No, Radio_Button
Validation Rules: None

Value	Value Description
<0.5	
0.6-1.0	
1.1-1.5	
1.6-2.0	
>2.0	
Unknown	

Tumor, % of Specimen

Definition: Amount of the accession involved by invasive tumor
Required: Yes; Enterable Field: No, Radio_Button
Validation Rules: None

Value	Value Description
< 5	

5 - < 25	
25 - < 50	
50 - < 75	
75 - 100	
Not Applicable	
Unknown	

Tumor Laterality

Definition: Laterality of invasive tumor in the prostate
Required: Yes; Enterable Field: No, Radio_Button
Validation Rules: None

Value	Value Description
One-right	
One-left	
Two	
One NOS	
Not Applicable (Default)	
Unknown	

Multifocal Disease

Definition: Is there more than one discrete focus of invasive tumor. Multifocal tumors MUST be separated by a certain distance (1-2 cm) so that the chance of artifactual sectioning is eliminated.
Required: Yes; Enterable Field: No, Radio_Button
Validation Rules: None

Value	Value Description
Yes	
No	Staging T1 desc
Not Applicable (Default)	
Unknown	

In Situ Neoplasia

High Grade PIN

Definition: Was High Grade PIN identified in this accession?
Required: Yes; Enterable Field: No, Combo_Box
Validation Rules: None

Value	Value Description
Unknown	
Present NOS	
Focal within tumor	
Focal away from tumor	Does this prostate contain HGPIN
Multifocal	
Not Present	
N/A	

Tumor findings and Attributes

Extraprostatic Extension

Definition: Was Extracapsular Extension Identified in this Accession?
Required: Yes; Enterable Field: No, Radio_Button
Validation Rules: None

Value	Value Description
Yes	
Yes - Extensive	
No	
N/A	
Unknown	

Seminal Vesicle Invasion

Definition: Code describes the status of surgical margins in the specimen.

Required: Yes; Enterable Field: No, Radio_Button
Validation Rules: None

Value	Value Description
Yes	
Yes - Extensive	
No	
N/A	
Unknown	

Perineural Invasion

Definition: Was perineural invasion identified in this accession
Required: Yes; Enterable Field: No, Radio_Button
Validation Rules: None

Value	Value Description
Yes	
No	
N/A	
Unknown	

Angiolymphatic Invasion

Definition: Was Angiolymphatic Invasion identified in this accession
Required: Yes; Enterable Field: No, Radio_Button
Validation Rules: None

Value	Value Description
Yes	
No	
N/A	
Unknown	

Surgical Margin Involvement

Definition:
Required: Yes; Enterable Field: No, Radio_Button
Validation Rules: None

Value	Value Description
Yes	
Yes - Extensive	
No	
Not Applicable	
Unknown	

Lymph Node Examination

Number of Lymph Nodes Examined

Definition: Record the total number of regional LN examined by a pathologist for this specimen.
Required: Yes; Enterable Field: Yes
Validation Rules: None
Data Type: Number; Default Value: -1; Data Range: 0 - 99

Lymph Node Examined Range

Definition: THIS ELEMENT IS FOR DATA QUERY VIEWER PURPOSE ONLY.
Required: Yes; Enterable Field: No, Radio_Button
Validation Rules:

Value	Value Description
0	
1-5	
>5	
Unknown	

Number of Lymph Nodes Positive
Definition: Identifies positive regional lymph nodes found in the specimen
Required: Yes; Enterable Field: Yes
Validation Rules: None
Data Type: Number; Default Value: -1; Data Range: -1 - 99

Lymph Node Positive Range
Definition: THIS ELEMENT IS FOR DATA QUERY VIEWER PURPOSE ONLY.
Required: Yes; Enterable Field: No, Radio_Button
Validation Rules:

Value	Value Description
0	
1-5	
>5	
Unknown	

Extranodal Extension
Definition: Was extranodal extension seen in the specimen? Do not record yes just because there is metastatic spread.
Required: Yes; Enterable Field: No, Radio_Button
Validation Rules: None

Value	Value Description
Yes	
No	
N/A	
Unknown	

Largest Lymph Node Metastasis (cm)
Definition: Largest metastatic tumor nodule in centimeters
Required: Yes; Enterable Field: Yes
Validation Rules: None
Data Type: Number; Default Value: -1; Data Range: -1 - 99

Size of Tumor in Lymph Node Range
Definition: THIS ELEMENT IS FOR DATA QUERY VIEWER PURPOSE ONLY.
Required: Yes; Enterable Field: No, Radio_Button
Validation Rules:

Value	Value Description
<0.5	
0.6-1.0	
1.1-1.5	
1.6-2.0	
>2.0	
Unknown	

Lymph Node Ratio
Definition:
Required: Yes; Enterable Field: Yes
Validation Rules:
Data Type: Number; Default Value: No default; Data Range:

Tissue Processing

Total Number of Paraffin Blocks
Definition: Total number of paraffin tissue blocks associated with this accession
Required: Yes; Enterable Field: Yes
Validation Rules: That you have available. There may be a difference with what you enter here and what you have entered at the block level.
Data Type: Number; Default Value: -1; Data Range: -1 - 99

Paraffin Block Range
Definition: THIS ELEMENT IS FOR DATA QUERY VIEWER PURPOSE ONLY.
Required: Yes; Enterable Field: No, Radio_Button

Validation Rules:

Value	Value Description
<5	
6-10	
>10	

Frozen Tissue Available

Definition: Is frozen tissue from this accession available
Required: Yes; Enterable Field: No, Radio_Button
Validation Rules: None

Value	Value Description
Yes	
No	
Unknown (Default)	

Warm Ischemia Time (min)

Definition: If tissue was frozen, what was the warm ischemic time.
Required: Yes; Enterable Field: Yes
Validation Rules: None
Data Type: Number; Default Value: -1; Data Range: -1 - 999

Ischemia Time Range (min)

Definition: THIS ELEMENT IS FOR DATA QUERY VIEWER PURPOSE ONLY.
Required: Yes; Enterable Field: No, Radio_Button
Validation Rules:

Value	Value Description
<15	
15-30	
31-60	
>60	

Are Blood Products Available

Definition: Is there a sample blood or blood product associated with this accession
Required: Yes; Enterable Field: No, Radio_Button
Validation Rules: None

Value	Value Description
Yes	
No	
Unknown	

Are Urine Samples Available

Definition:
Required: Yes; Enterable Field: No, Radio_Button
Validation Rules: None

Value	Value Description
Yes	
No	
Unknown	

Is this accession currently available?

Definition:
Required: Yes; Enterable Field: No, Radio_Button
Validation Rules: None

Value	Value Description
Yes (Default)	
No	

Data on Individual Tissue Blocks and Fluid Aliquots

Block Id (Label)

Definition: Alphanumeric string that identifies the block or fluid in question. This string must not identify the patient

Required: Yes; Enterable Field: Yes

Validation Rules: None

Data Type: Character; Default Value: No default; Maximum length: 10

Complete Block Id

Definition:

Required: Yes; Enterable Field: Yes

Validation Rules:

Data Type: Number; Default Value: No default; Data Range:

Sample Type

Definition:

Required: Yes; Enterable Field: No, Radio_Button

Validation Rules: None

Value	Value Description
Prostate	
Lymph Node	
Metastatic Site	
Whole Blood	
Serum	
Plasma	
Buffy Coat	
RBCs	
Urine	
Other (Default)	

Procedure Type

Definition:

Required: Yes; Enterable Field: No, Combo_Box

Validation Rules: None

Value	Value Description
Biopsy	
Resection	
Tissue Aspiration	
Blood Draw	
Fluid	
Other	
Unknown (Default)	
Not Applicable	

Sample Processing

Definition:

Required: Yes; Enterable Field: No, Radio_Button

Validation Rules: None

Value	Value Description
Formalin Paraffin	
Other Fixation	
Frozen Bulk	
Frozen OCT	
Unknown	

Freezer Temperature

Definition:

Required: Yes; Enterable Field: No, Radio_Button

Validation Rules: None

Value	Value Description
-40	
-40 to -180	
< -180	
Not Applicable	

Invasive Tumor Present

Definition:
Required: Yes; Enterable Field: No, Radio_Button
Validation Rules: None

Value	Value Description
Yes	
No	
No - In Situ only	
N/A	
Unknown (Default)	

Primary or Metastatic Tumor Present

Definition:
Required: Yes; Enterable Field: No, Radio_Button
Validation Rules: None

Value	Value Description
Primary	
Metastatic	
Both	
Neither	
N/A	
Unknown	

Tumor Size , Maximum Diameter (cm)

Definition:
Required: Yes; Enterable Field: Yes
Validation Rules: None
Data Type: Number; Default Value: -1; Data Range: 0.01 - 5.00

Url to Image

Definition:
Required: No; Enterable Field: Yes
Validation Rules: None
Data Type: Character; Default Value: No default; Maximum length: 128

Sample Availability

Definition: Is this sample available now?
Required: Yes; Enterable Field: No, Radio_Button
Validation Rules: None

Value	Value Description
Yes (Default)	
No	

Histology

Definition:
Required: Yes; Enterable Field: No, Combo_Box
Validation Rules:

Value	Value Description
Adenocarcinoma NOS	
Ductal Adenocarcinoma	
Benign Tissue Only	
In Situ Neoplasia Only	

Mucinous Adenocarcinoma	
Signet Ring Adenocarcinoma	
Basal Cell Adenocarcinoma	
Undiff Non-Small Cell Carcinoma	
Sarcomatoid Carcinoma	
Small Cell Anaplastic Carcinoma	
Squamous or Adenosquamous Ca	
Mesenchymal Tumor NOS	
Lymphoma	
Neuroendocrine Carcinoma	
Transitional Carcinoma	
Other	
N/A -- For Blood fluids	
Unknown	

Primary Gleason Grade

Definition:

Required: Yes; Enterable Field: No, Radio_Button

Validation Rules:

Value	Value Description
1	
2	
3	
4	
5	
N/A	
Unknown	

Secondary Gleason Grade

Definition:

Required: Yes; Enterable Field: No, Radio_Button

Validation Rules:

Value	Value Description
1	
2	
3	
4	
5	
N/A	
Unknown	

High Grade PIN

Definition:

Required: Yes; Enterable Field: No, Radio_Button

Validation Rules:

Value	Value Description
Yes	
No	

Extracapsular Extension

Definition:

Required: Yes; Enterable Field: No, Radio_Button

Validation Rules:

Value	Value Description
Yes	
No	

Seminal Vesicle Invasion

Definition:

Required: Yes; Enterable Field: No, Radio_Button
Validation Rules:

Value	Value Description
Yes	
No	

Perineural Invasion

Definition:
Required: Yes; Enterable Field: No, Radio_Button
Validation Rules:

Value	Value Description
Yes	
No	

Angio-lymphatic Invasion

Definition:
Required: Yes; Enterable Field: No, Radio_Button
Validation Rules:

Value	Value Description
Yes	
No	

Surgical Margin Involvement

Definition:
Required: Yes; Enterable Field: No, Radio_Button
Validation Rules:

Value	Value Description
Yes	
No	

Block Gleason

Definition:
Required: Yes; Enterable Field: Yes
Validation Rules: None
Data Type: Number; Default Value: No default; Data Range:

Image Available

Definition:
Required: Yes; Enterable Field: No, Check_Box
Validation Rules:

Value	Value Description
Yes	
No	

Tumor Marker Events

Marker Results

Marker Type

Definition:
Required: Yes; Enterable Field: No, Combo_Box
Validation Rules: None

Value	Value Description
PSA (Default)	

Months +/- Diagnosis

Definition: Months before or after diagnosis (use report months before diagnosis with negative numbers)

Required: Yes; Enterable Field: Yes
Validation Rules: None
Data Type: Number; Default Value: -999; Data Range: -999 - 9999

Marker Level (ng)

Definition: PSA level reported in ng
Required: Yes; Enterable Field: Yes
Validation Rules: None
Data Type: Number; Default Value: -1; Data Range: 0.00 - 9999.00

Marker Diagnosis Context

Definition: Reported PSA value that is the before (Pre-Dx), just prior to (Diagnostic), or after (Post-Dx) the diagnostic biopsy date.
Required: Yes; Enterable Field: No, Radio_Button
Validation Rules:

Value	Value Description
Pre Diagnostic	All PSA values prior to the diagnostic biopsy and multiple values allowed.
Diagnostic	PSA value that prompted a Diagnostic Biopsy. Should be immediately prior to the diagnostic biopsy and only one value allowed.
Post Diagnostic	All PSA values after the diagnostic biopsy and multiple values allowed.

How Reported

Definition: Source of PSA information
Required: Yes; Enterable Field: No, Radio_Button
Validation Rules: None

Value	Value Description
Medical Record (Default)	
From MD	
From Patient	
Other	
Unknown	

Marker Events

Definition: THIS ELEMENT IS FOR DATA QUERY VIEWER PURPOSE ONLY.
Required: Yes; Enterable Field: No, Radio_Button
Validation Rules:

Value	Value Description

Therapy Events

Cancer Therapy

Therapy Type

Definition: General type of PCA therapy
Required: Yes; Enterable Field: No, Combo_Box
Validation Rules: None

Value	Value Description
Surgical Resection	
Radiation Rx	
Hormonal Rx	
Chemo Rx	
Biologic Response Modifier	
Other Rx	
Unknown	
Watchful Waiting	

Months After Diagnosis

Definition: Indicates when therapy was begun (months from diagnosis)

Required: Yes; Enterable Field: Yes
Validation Rules: None
Data Type: Number; Default Value: -999; Data Range: -999 - 999

Rx Protocol

Definition: Description of the specific therapy given
Required: Yes; Enterable Field: No, Combo_Box
Validation Rules: None

Value	Value Description
Cancer Directed Surgery	
Non Cancer Directed Surgery	
External Radiation	
Internal Radiation (implants)	
Orchectomy - Surgical	
Orchectomy - Medical	
Single Agent Chemotherapy	
Multiple Agent Chemotherapy	
Immuno-therapy	
Cryotherapy	
Other	
Unknown (Default)	
Watchful Waiting	

Anatomic Location

Definition: Therapy to primary or metastatic site?
Required: Yes; Enterable Field: No, Radio_Button
Validation Rules: None

Value	Value Description
Primary Site	
Known Metastatic Site	
Site Not Applicable	
Unknown (Default)	

Therapy Events

Definition: THIS ELEMENT IS FOR DATA QUERY VIEWER PURPOSE ONLY.
Required: Yes; Enterable Field: Yes
Validation Rules:
Data Type: Number; Default Value: No default; Data Range:
