# Supplementary material for: A Novel Cross-Disciplinary Multi-Institute Approach to Translational Cancer Research: Lessons Learned from Pennsylvania Cancer Alliance Bioinformatics Consortium (PCABC)
Source: Cancer Inform. 2007 Jun 8;3:255–74. (PMC2675833)
Supplement: Breast CDEs — (additional files #9) [file cin-03-255-s9.pdf]

Additional File #9

Pennsylvania Cancer Alliance Bioinformatics Consortium (PCABC)

Breast Common Data Elements

vMarch 2006

Cancer Centers

Cancer Center

Site Name

Definition:

Required: Yes; Enterable Field: No, Radio_Button

Validation Rules:

Value	Value Description
ACC, UPenn	
FCCC	
KCC, TJU	
PSU	
UPCI	
Wistar	
Geisinger	

Case Identification

Case Identification

Case Accession Number

Definition: This is a de-identified number given by the submitting institution to index all data on a given case.

Required: Yes; Enterable Field: Yes

Validation Rules: None

Data Type: Character; Default Value: No default; Maximum length: 32

CDE version

Definition: Version 2 (1/15/2004 aap) with NAACR elements

Required: Yes; Enterable Field: Yes

Validation Rules: This version includes changes made after including NAACR standards.

Data Type: Number; Default Value: No default; Data Range:

Research Consent Elements

Consent Status

Tissue Consent Status

Definition: Is there a valid consent in place for use of this patient's tissue in research?

Required: Yes; Enterable Field: No, Radio_Button

Validation Rules: None

Value	Value Description
Valid	
Not Valid	
Unknown (Default)	

Data Consent Status

Definition: Is there a valid consent in place for use of this patient's data in research?

Required: Yes; Enterable Field: No, Radio_Button

Validation Rules: None

Value	Value Description
Valid	
Not Valid	
Unknown (Default)	

Demographics and History

Base Demographics

Age at Diagnosis

Definition: Age of patient at diagnoses in complete years.
Required: Yes; Enterable Field: Yes
Validation Rules: None
Data Type: Number; Default Value: -1; Data Range: -1 - 99

Age Range at Diagnosis

Definition: THIS ELEMENT IS FOR DATA QUERY VIEWER PURPOSE ONLY.
Required: Yes; Enterable Field: No, Radio_Button
Validation Rules:

Value	Value Description
0-20	
21-30	
31-40	
41-50	
61-70	
71-80	
>80	

Age Range at Diagnosis

Definition: THIS ELEMENT IS FOR DATA QUERY VIEWER PURPOSE ONLY.
Required: Yes; Enterable Field: No, Radio_Button
Validation Rules:

Value	Value Description

Race

Definition: Code the patient?s race. Race is coded separately from Spanish/Hispanic Orgin.
Required: Yes; Enterable Field: No, Radio_Button
Validation Rules: None

Value	Value Description
Native American	
Asian	
African American	
Pacific Islander	
White	
Other	
Unknown (Default)	

Hispanic Origin

Definition: Code identifying persons of Spanish or Hispanic origin.
Required: Yes; Enterable Field: No, Radio_Button
Validation Rules: None

Value	Value Description
Yes	
No	
Unknown (Default)	

Patient Disease History

Last Mammogram before Dx (months)

Definition: Consider definition as last mammogram before histological diagnosis
Required: Yes; Enterable Field: No, Radio_Button
Validation Rules: None

Value	Value Description
< 6	
6 - 12	
13 - 24	
25 - 60	
Never	
Unknown (Default)	

Breast Ultrasound (months before Dx)

Definition: Breast Ultrasound (months before Dx)
Required: Yes; Enterable Field: No, Radio_Button
Validation Rules: None

Value	Value Description
< 6	
6 - 12	
13 - 24	
25 - 60	
Never	
Unknown (Default)	

Previous Dx of Breast CA

Definition: Previous Dx of Breast CA
Required: Yes; Enterable Field: No, Radio_Button
Validation Rules: None

Value	Value Description
Yes - Right	
Yes - Left	
Yes - Bilateral	
No	
Unknown (Default)	

Previous Breast CA Dx (months before Dx)

Definition: Date of Previous Breast CA Dx (months before Current Dx)
Required: Yes; Enterable Field: No, Radio_Button
Validation Rules: None

Value	Value Description
< 6	
6 - 12	
13 - 24	
25 - 60	
61 - 120	
> 120	
Not Applicable	
Unknown (Default)	

Family Disease History

Family History of Breast Cancer

Definition: Family History of Breast Cancer
Required: Yes; Enterable Field: No, Radio_Button
Validation Rules: None

Value	Value Description
Yes	
No	
Unknown (Default)	

Family Disease History Detail

Maternal/Paternal

Definition: Paternal or Maternal Side
Required: Yes; Enterable Field: No, Radio_Button
Validation Rules: None

Value	Value Description
Paternal	
Maternal	
Unknown	
Not Applicable	

Relationship

Definition: Relation to the patient
Required: Yes; Enterable Field: No, Combo_Box
Validation Rules: None

Value	Value Description
Parent	
Sibling	
Child	
Aunt/Uncle	
Grandparent	
First Cousin	
Unknown	

Male?

Definition: Is this family member male?
Required: Yes; Enterable Field: No, Radio_Button
Validation Rules: None

Value	Value Description
Yes	
No	
Unknown	

Patient Exposures

Current Tobacco use

Definition: NAACCR has not adopted standards for this item.
Required: Yes; Enterable Field: No, Radio_Button
Validation Rules: None

Value	Value Description
Yes	
No	
Unknown (Default)	

Current ETOH use

Definition: NAACCR has not adopted standards for this item.
Required: Yes; Enterable Field: No, Radio_Button
Validation Rules: None

Value	Value Description
Yes	
No	
Unknown (Default)	

Total No Of Pregnancies

Definition: THIS ELEMENT IS FOR DATA QUERY VIEWER PURPOSE ONLY.
Required: Yes; Enterable Field: No, Radio_Button
Validation Rules:

Value	Value Description
0	
1	
2	
3	
4	
5	
>5	

Number of Total Pregnancies

Definition: Number of pregnancies
Required: Yes; Enterable Field: Yes
Validation Rules: None
Data Type: Number; Default Value: -1; Data Range: -1 - 15

Term Pregnancy

Definition:
Required: Yes; Enterable Field: No, Radio_Button
Validation Rules:

Value	Value Description
0	
1	
2	
3	
4	
5	
>5	

Number of Term Pregnancies

Definition: Number of Term Pregnancies
Required: Yes; Enterable Field: Yes
Validation Rules: None
Data Type: Number; Default Value: -1; Data Range: -1 - 20

Miscarrage/Abortions

Definition: THIS ELEMENT IS FOR DATA QUERY VIEWER PURPOSE ONLY.
Required: Yes; Enterable Field: No, Radio_Button
Validation Rules:

Value	Value Description
0	
1	
2	
3	
4	
5	
>5	

Number of Miscarriages/Abortions

Definition: Number of Miscarriages/Abortions
Required: Yes; Enterable Field: Yes
Validation Rules: None
Data Type: Number; Default Value: -1; Data Range: -1 - 20

Still Menstruating at Dx

Definition: Still Menstruating at Dx
Required: Yes; Enterable Field: No, Radio_Button
Validation Rules: None

Value	Value Description

No	
Unknown (Default)	

Menopause Age Range

Definition: THIS ELEMENT IS FOR DATA QUERY VIEWER PURPOSE ONLY.

Required: Yes; Enterable Field: No, Radio_Button

Validation Rules:

Value	Value Description
<20	
20-29	
30-39	
>39	

Age at Menopause

Definition:

Required: Yes; Enterable Field: Yes

Validation Rules: None

Data Type: Number; Default Value: -1; Data Range: -1 - 99

Current Hormone Use

Definition:

Required: Yes; Enterable Field: No, Radio_Button

Validation Rules: None

Value	Value Description
Yes	
No	
Unknown (Default)	

Progression and Outcomes

Clinical Staging (at diagnosis)

Clinical Staging AJCC Version

Definition: A code that indicates the edition of the AJCC manual used to stage the tumor. This applies to the manually coded AJCC fields It does not apply t o Derived AJCC T,N,M and AJCC Stage Group fields.

Required: Yes; Enterable Field: No, Radio_Button

Validation Rules: None

Value	Value Description
2nd Edition	
3rd Edition	
4th Edition	
5th Edition	
6th Edition (Default)	
Unknown	

T Stage, Clinical

Definition: Detailed site-specific codes for the clinical tumor (T) as defined by AJCC and recorded by the physician.

Required: Yes; Enterable Field: No, Radio_Button

Validation Rules: None

Value	Value Description
TX (Default)	
T0	
Tis	
T1	
T1mic	
T1a	
T1b	
T1c	
T2	

T3	
T4	
T4a	
T4b	
T4c	
T4d	
Unknown	

N Stage, Clinical

Definition: Detailed site-specific codes for the clinical nodes (N) as defined by AJCC and recorded by the physician.

Required: Yes; Enterable Field: No, Radio_Button

Validation Rules: None

Value	Value Description
NX (Default)	
N0	
N1	
N2	
N2a	
N2b	
N3	
N3a	
N3b	
N3c	
Unknown	

M Stage, Clinical

Definition: Detailed site-specific codes for the clinical metastases (M) as defined by AJCC and recorded by the physician

Required: Yes; Enterable Field: No, Radio_Button

Validation Rules: None

Value	Value Description
MX (Default)	
M0	
M1	
Unknown	

cTNM

Definition: THIS ELEMENT IS FOR DATA QUERY VIEWER PURPOSE ONLY.

Required: Yes; Enterable Field: Yes

Validation Rules:

Data Type: Number; Default Value: No default; Data Range:

Pathologic Staging

Pathologic Staging, AJCC Version

Definition: A code that indicates the edition of the AJCC manual used to stage the tumor. This applies to the manually coded AJCC fields It does not apply t o Derived AJCC T,N,M and AJCC Stage Group fields.

Required: Yes; Enterable Field: No, Radio_Button

Validation Rules: None

Value	Value Description
2nd Edition	
3rd Edition	
4th Edition	
5th Edition	
6th Edition (Default)	
Unknown	

T Stage, Pathologic

Definition: Detailed site-specific codes for the pathological tumor (T) as defined by

AJCC and recorded by the physician

Required: Yes; Enterable Field: No, Radio_Button

Validation Rules: None

Value	Value Description
TX (Default)	
T0	
Tis	
T1	
T1mic	
T1a	
T1b	
T1c	
T2	
T3	
T4	
T4a	
T4b	
T4c	
T4d	
Unknown	

N Stage, Pathologic

Definition: Detailed site-specific codes for the pathological nodes (N) as defined by AJCC and recorded by the physician.

Required: Yes; Enterable Field: No, Radio_Button

Validation Rules: None

Value	Value Description
pNX (Default)	
pN0	
pN0(i-)	
pN0(i+)	
pN0(mol-)	
pN0(mol+)	
pN1	
pN1mi	
pN1a	
pN1b	
pN1c	
pN2	
pN2a	
pN2b	
pN3	
pN3a	
pN3b	
pN3c	
Unknown	

M Stage, Pathologic

Definition: Detailed site-specific codes for the pathological metastases (M) as defined by AJCC and recorded by the physician

Required: Yes; Enterable Field: No, Radio_Button

Validation Rules: None

Value	Value Description
pMX (Default)	
pM0	
pM1	
Unknown	

pTNM

Definition: THIS ELEMENT IS FOR DATA QUERY VIEWER PURPOSE ONLY.
Required: Yes; Enterable Field: Yes
Validation Rules:
Data Type: Number; Default Value: No default; Data Range:

Most Recent Followup

Most Recent Follow Up (Months from Dx)

Definition: Date of last contact with the patient, or date of death.
Required: Yes; Enterable Field: Yes
Validation Rules: None
Data Type: Number; Default Value: -1; Data Range: -1 - 999

Followup (Months)

Definition:
Required: Yes; Enterable Field: No, Radio_Button
Validation Rules:

Value	Value Description
<13	
13-24	
25-36	
37-60	
>60	

Vital Status

Vital Status at Most Recent Follow Up

Definition: Vital Status of the patient as of the date entered in item 1750 (Date of Last Contact).
Required: Yes; Enterable Field: No, Radio_Button
Validation Rules: None

Value	Value Description
Alive	
Dead	
Unknown (Default)	

Months from Diagnosis to Death

Definition: Months between diagnosis and death
Required: Yes; Enterable Field: Yes
Validation Rules: None
Data Type: Number; Default Value: -1; Data Range: -1 - 999

Post Diagnosis Survival

Definition:
Required: Yes; Enterable Field: No, Radio_Button
Validation Rules:

Value	Value Description
<13	
13-24	
25-36	
37-60	
>60	

Follow up

Definition:
Required: Yes; Enterable Field: Yes
Validation Rules:
Data Type: Number; Default Value: No default; Data Range:

First Recurrence

Months to First Recurrence

Definition: Months between diagnosis and first recurrence
Required: Yes; Enterable Field: Yes
Validation Rules: None

Data Type: Number; Default Value: -1; Data Range: 1 - 999

First Recurrence Type

Definition: Code for the type of first recurrence after a period of documented disease-free intermission or remission.

Required: Yes; Enterable Field: No, Combo_Box

Validation Rules: None

Value	Value Description
None - disease free	
In situ	
Local	
Regional - NOS	
Regional - Tissue	
Regional - Lymph Nodes	
Distant	
Never Disease Free	
Recurred - Site Unknown	
Unknown (Default)	

Diagnosis To First Recurrence(Months)

Definition: THIS ELEMENT IS FOR DATA QUERY VIEWER PURPOSE ONLY.

Required: Yes; Enterable Field: No, Radio_Button

Validation Rules:

Value	Value Description
<13	
13-24	
25-36	
37-60	
>60	

Recurrence Events

Definition:

Required: Yes; Enterable Field: Yes

Validation Rules:

Data Type: Number; Default Value: No default; Data Range:

Overall Metastatic Progression

First Distant Metastasis

Definition: Code for the distant site or sites in which the tumor has recurred.

Required: Yes; Enterable Field: No, Combo_Box

Validation Rules: None

Value	Value Description
No First Location	
Peritoneum	
Lung	
Pleura	
Liver	
Bone	
CNS	
Skin	
Distant Lymph Nodes	
Other - Generalized - NOS	

Second Distant Metastasis

Definition: Code for the distant site or sites in which the tumor has recurred

Required: Yes; Enterable Field: No, Combo_Box

Validation Rules: None

Value	Value Description
No Second Location	

Other - Generalized - NOS	
Peritoneum	
Lung	
Pleura	
Lung	
Liver	
Bone	
CNS	
Skin	
Distant Lymph Nodes	

Third Distant Metastasis

Definition: Code for the distant site or sites in which the tumor has recurred.
Required: Yes; Enterable Field: No, Combo_Box
Validation Rules: None

Value	Value Description
No Third Location	
Peritoneum	
Lung	
Pleura	
Liver	
bone	
CNS	
Skin	
Distant Lymph Nodes	
Other - Generalized - NOS	

Time Line

Overall TL

Definition: THIS ELEMENT IS FOR DATA QUERY VIEWER PURPOSE ONLY.
Required: Yes; Enterable Field: Yes
Validation Rules:
Data Type: Number; Default Value: No default; Data Range:

Therapy TL

Definition: THIS ELEMENT IS FOR DATA QUERY VIEWER PURPOSE ONLY.
Required: Yes; Enterable Field: Yes
Validation Rules:
Data Type: Number; Default Value: No default; Data Range:

Procedure TL

Definition: THIS ELEMENT IS FOR DATA QUERY VIEWER PURPOSE ONLY.
Required: Yes; Enterable Field: Yes
Validation Rules:
Data Type: Number; Default Value: No default; Data Range:

Tissue Accession Data

Date and Procedure Elements

De-identified Accession ID

Definition: De-identified accession ID from submitting institution
Required: Yes; Enterable Field: Yes
Validation Rules: None
Data Type: Character; Default Value: No default; Maximum length: 32

Months between Dx and Accession

Definition: The time in months between the Diagnosis and the Accession (can be a negative for benign biopsies before the Dx)
Required: Yes; Enterable Field: Yes
Validation Rules: None
Data Type: Number; Default Value: -1; Data Range: -1 - 999

Diagnosis To Accession (Months)

Definition:
Required: Yes; Enterable Field: No, Radio_Button

Validation Rules:

Value	Value Description
<13	
13-24	
25-36	
37-60	
>60	

Procedure Site

Definition:

Required: Yes; Enterable Field: No, Combo_Box

Validation Rules: None

Value	Value Description
Breast - Primary	
Breast - Metastasis/Recurrence	
Lymph Nodes	
Other	
Not Applicable	
Unknown	

Procedure Type

Definition: Site-specific codes for the type of surgery to the primary site performed as part of the first course of treatment.Records the surgical removal of distant lymph nodes or other tissues/organs beyond primary site.

Required: Yes; Enterable Field: No, Combo_Box

Validation Rules: None

Value	Value Description
Mastectomy (NOS)	
Lumpectomy (NOS)	
Breast Biopsy	
Breast Aspiration	
Lymph Node Exploration Only	
Metastatasis Exploration Only	
Blood or Fluid Only	
Mastectomy and Lymph Node	
Combination (NOS)	Any Combination of procedure types listed above except "Mastectomy and Lymph Node exploration".
Other	
Unknown (Default)	

Procedure Events

Definition:

Required: Yes; Enterable Field: Yes

Validation Rules:

Data Type: Number; Default Value: No default; Data Range:

Diagnosis and Grade

Primary Malignant Histology

Definition: Codes for the histological type of the tumor being reported using ICD-O-3.

Required: Yes; Enterable Field: No, Combo_Box

Validation Rules: None

Value	Value Description
Carcinoma in situ, NOS	
Invasive Cancer NOS	
Lobular carcinoma, NOS	

Lobular carcinoma in situ, NOS	
Ductal NOS	
Intraductal carcinoma , noninfiltrating, NOS	
Noninfiltrating intraductal, papillary adenocarcinoma	
Inflammatory carcinoma	
Mucinous adenocarcinoma	
Medullary carcinoma, NOS	
Medullary with Lymphoid Stroma	
Tubular adenocarcinoma	
Paget's disease, mammary	
Paget's disease and infiltrating duct carcinoma of breast	
Paget's disease and intraductal carcinoma of breast	
Squamous cell carcinoma, NOS	
Adenoid cystic carcinoma	
Phyllodes tumor, malignant	
Cribriform carcinoma NOS	
Carcinoma Undifferentiated, NOS	
Cribriform carcinoma in situ	
Infiltrating duct carcinoma	
Comedocarcinoma, noninfiltrating	
Secretory carcinoma of breast	
Intraductal carcinoma and Lobular carcinoma, in situ	
Carcinosarcoma, NOS	
Carcinoma NOS	
Benign Only	
Metastasis	
Not Applicable	
Unknown (Default)	

Tumor Differentiation

Definition: Code for the grade or degree of differentiation of the reportable tumor
Required: Yes; Enterable Field: No, Radio_Button
Validation Rules: None

Value	Value Description
Well	
Moderate	
Poor	
Not Applicable	
Unknown (Default)	

Nuclear Grade

Definition: Nuclear Grade
Required: Yes; Enterable Field: No, Radio_Button
Validation Rules: None

Value	Value Description
Grade 1 (low,1pt)	
Grade 2 (Intermediate,2pt)	
Grade 3 (High,3pt)	
Not Applicable	
Unknown (Default)	

Histologic (Tubule Formation) Grade

Definition: Histologic (tubule formation) grade
Required: Yes; Enterable Field: No, Radio_Button
Validation Rules: None

Value	Value Description
< 10% (minimum, 1 pt)	
10% - 75% (moderate, 2 pt)	
> 75% (majority of tumor, 3 pt)	

Not Applicable	
Unknown (Default)	

Mitotic Activity Grade

Definition: Mitotic activity grade
Required: Yes; Enterable Field: No, Radio_Button
Validation Rules: None

Value	Value Description
Low Activity, 1pt	
Moderate Activity, 2pt	
Marked Activity, 3pt	
Not Applicable	
Unknown (Default)	

Primary Benign Non-neoplasia

Definition: Primary Benign Non-neoplasia
Required: Yes; Enterable Field: No, Combo_Box
Validation Rules: None

Value	Value Description
Fibrocystic Change	
Ductal Hyperplasia w/o Atypia	
Sclerosing Adenosis	
Microglandular Adenosis	
Adenosis NOS	
Stromal Fibrosis	
Duct Ectasia	
Fat Necrosis	
Diabetic Mastopathy	
Papillomatosis	
Intraductal Papilloma	
Collagenous Spherulosis	
Radial Scar	
Chemo/Radiation Effect	
Fibromatosis	
Other	
Normal	
Unknown	

Primary Benign Neoplasia

Definition: Primary Benign Neoplasia
Required: Yes; Enterable Field: No, Combo_Box
Validation Rules: None

Value	Value Description
Fibroadenoma NOS	
Fibroadenoma w Ductal Hyperplasia	
Breast Harmatoma	
Hemangioma	
Tubular Adenoma	
Myoepithelial Tumor	
Phylloides Tumor	
Granular Cell Tumor	
Other	
No Benign Neoplasia	
Unknown	

Tumor Size and Extent

Tumor Diameter (cm)

Definition: Maximum tumor diameter in cm
Required: Yes; Enterable Field: Yes

Validation Rules: None
Data Type: Number; Default Value: -1; Data Range: -1 - 30

Invasive Multifocal Disease

Definition: Is multifocal invasive tumor present
Required: Yes; Enterable Field: No, Radio_Button
Validation Rules: None

Value	Value Description
Yes	
No	
Not Applicable	
Unknown	

Tumor Size Range

Definition:
Required: Yes; Enterable Field: No, Radio_Button
Validation Rules:

Value	Value Description
<0.5	
0.5-1.0	
1.1-1.5	
1.6-2.0	
>2.0	
Unknown	

In Situ Neoplasia

Is DCIS Present

Definition:
Required: Yes; Enterable Field: No, Radio_Button
Validation Rules: None

Value	Value Description
Yes	
No	
Unknown	

Dominant Histologic Type of DCIS

Definition:
Required: Yes; Enterable Field: No, Combo_Box
Validation Rules: None

Value	Value Description
DCIS NOS	
Comedo	
Solid	
Cribriform	
Micropapillary	
Clinging	
Apocrine	
Intra-cystic(encysted papillary)	
Papillary Carcinoma in situ	
Not Applicable	
Unknown (Default)	

Is DCIS present with invasive cancer

Definition:
Required: Yes; Enterable Field: No, Radio_Button
Validation Rules: None

Value	Value Description

Yes	
No	
Not Applicable	
Unknown	

DCIS extent

Definition:

Required: Yes; Enterable Field: No, Radio_Button

Validation Rules: None

Value	Value Description
Focal	
Extensive	
Not Applicable	
Unknown (Default)	

Microcalcification (in DCIS)

Definition:

Required: Yes; Enterable Field: No, Radio_Button

Validation Rules: None

Value	Value Description
Present - involving benign ducts	
Present - involving DCIS	
Present - in benign and DCIS	
None	
Not Applicable	
Unknown (Default)	

Is DCIS at the margin

Definition:

Required: Yes; Enterable Field: No, Radio_Button

Validation Rules: None

Value	Value Description
Less than 1 mm	
> or = 1 mm to 10 mm	
Greater than 10 mm	
Involved NOS	
Not Involved	
Not Applicable	
Unknown (Default)	

Is LCIS present

Definition:

Required: Yes; Enterable Field: No, Radio_Button

Validation Rules: None

Value	Value Description
Yes	
No	
Unknown	

Is LCIS present with invasive cancer

Definition:

Required: Yes; Enterable Field: No, Radio_Button

Validation Rules: None

Value	Value Description
Yes	
No	
Not Applicable	

Unknown	

LCIS extent

Definition:
Required: Yes; Enterable Field: No, Radio_Button
Validation Rules: None

Value	Value Description
Focal	
Extensive	
Not Applicable	
Unknown (Default)	

Tumor findings and Attributes

Lymphovascular Invasion

Definition:
Required: Yes; Enterable Field: No, Radio_Button
Validation Rules: None

Value	Value Description
Yes	
No	
Not Applicable	
Unknown (Default)	

Perineural Invasion

Definition:
Required: Yes; Enterable Field: No, Radio_Button
Validation Rules: None

Value	Value Description
Yes	
No	
Not Applicable	
Unknown (Default)	

Lymphocytic/mononuclear Infiltrate

Definition: New
Required: Yes; Enterable Field: No, Radio_Button
Validation Rules: None

Value	Value Description
Not Significant	
Significant	
Not Applicable (Default)	
Unknown	

Microcalcification (in Invasive Tumor)

Definition: New
Required: Yes; Enterable Field: No, Radio_Button
Validation Rules: None

Value	Value Description
Yes	
No	
Not Applicable	
Unknown (Default)	

Necrosis present (in Invasive Tumor)

Definition: New
Required: Yes; Enterable Field: No, Radio_Button
Validation Rules: None

Value	Value Description
Yes	
No	
Not Applicable	
Unknown (Default)	

Necrosis Extent

Definition: New
Required: Yes; Enterable Field: No, Radio_Button
Validation Rules: None

Value	Value Description
None	
Scant	
Moderate	
Extensive	
Not Applicable	
Unknown (Default)	

Necrosis Type

Definition: New
Required: Yes; Enterable Field: No, Radio_Button
Validation Rules: None

Value	Value Description
Comedonecrosis,	
Non-comedonecrosis	
Other	
Not Applicable	
Unknown (Default)	

Margin Involvement (Invasive Tumor)

Definition:
Required: Yes; Enterable Field: No, Radio_Button
Validation Rules: None

Value	Value Description
Less than 1 mm	
>= 1 mm to 10 mm	
>10 mm	
Involved NOS	
Not involved	
Unknown (Default)	

Skin Ulceration Present

Definition:
Required: Yes; Enterable Field: No, Radio_Button
Validation Rules: None

Value	Value Description
Yes	
No	
Unknown	
Not Applicable (Default)	

Dermal lymphatic invasion

Definition: New
Required: Yes; Enterable Field: No, Radio_Button
Validation Rules: None

Value	Value Description

Yes	
No	
Not Applicable (Default)	
Unknown	

Paget's Disease of the Nipple

Definition: New

Required: Yes; Enterable Field: No, Radio_Button

Validation Rules: None

Value	Value Description
Yes	
No	
Not Applicable (Default)	
Unknown	

Estrogen Receptor Status

Definition:

Required: Yes; Enterable Field: No, Radio_Button

Validation Rules: None

Value	Value Description
Postitive	
Negative	
Not Applicable	
Unknown	

Progesterone Receptor Status

Definition:

Required: Yes; Enterable Field: No, Radio_Button

Validation Rules: None

Value	Value Description
Postitive	
Negative	
Not Applicable	
Unknown	

HER 2 Neu Receptor Status

Definition:

Required: Yes; Enterable Field: No, Radio_Button

Validation Rules: None

Value	Value Description
Positive	
Negative	
Not Applicable	
Unknown	

Lymph Node Examination

Sentinel Biopsy Performed

Definition: Sentinal Lymph Node Sampled

Required: Yes; Enterable Field: No, Radio_Button

Validation Rules: None

Value	Value Description
Yes	
No	
Not Applicable	
Unknown	

Sentinel Node Site

Definition:

Required: Yes; Enterable Field: No, Combo_Box
Validation Rules: None

Value	Value Description
Axillary	
Supraclavicular	
Internal Mammary	
Other	
Not Applicable	
Unknown (Default)	

Sentinel Node, Biopsy Results

Definition:
Required: Yes; Enterable Field: No, Radio_Button
Validation Rules: None

Value	Value Description
Positive	
Positive with ICH only	
Negative	
Not Applicable	
Unknown	

Sentinel Node, Largest Met

Definition:
Required: Yes; Enterable Field: No, Radio_Button
Validation Rules: None

Value	Value Description
<=2mm	
>2 mm	
Not Applicable (Default)	
Unknown	

Number of Nodes Examined

Definition: Record the total number of regional LN examined by a pathologist. (This includes LN removed from a separate procedure from removal of the primary tumor.) (NAACCR 2840)
Required: Yes; Enterable Field: Yes
Validation Rules: None
Data Type: Number; Default Value: -1; Data Range: -1 - 99

Lymph Node Examined Range

Definition:
Required: Yes; Enterable Field: No, Radio_Button
Validation Rules:

Value	Value Description
0	
1-5	
>5	
Unknown	

Number of Nodes Positive

Definition: Identifies positive regional lymph nodes involved at time of diagnosis. (NAACCR 2830)
Required: Yes; Enterable Field: Yes
Validation Rules: None
Data Type: Number; Default Value: -1; Data Range: -1 - 99

Lymph Node Positive Range

Definition:
Required: Yes; Enterable Field: No, Radio_Button
Validation Rules:

Value	Value Description
0	
1-5	
>5	
Unknown	

Largest Lymph Node Met

Definition:
Required: Yes; Enterable Field: No, Radio_Button
Validation Rules: None

Value	Value Description
< 0.2 to 2 mm by IHC only	
< 0.2 to 2 mm by H&E	
2 mm to 2 cm	
> 2 cm	
Not Applicable	
Unknown (Default)	

Extranodal Extension

Definition:
Required: Yes; Enterable Field: No, Radio_Button
Validation Rules: None

Value	Value Description
Yes	
No	
Not Applicable (Default)	
Unknown	

Lymph Node Ratio

Definition:
Required: Yes; Enterable Field: Yes
Validation Rules:
Data Type: Number; Default Value: No default; Data Range:

Tissue Processing

Total Number of Paraffin Blocks

Definition:Total number of paraffin tissue blocks associated with this accession
Required: Yes; Enterable Field: Yes
Validation Rules: None
Data Type: Number; Default Value: -1; Data Range: -1 - 99

Paraffin Block Range

Definition:THIS ELEMENT IS FOR DATA QUERY VIEWER PURPOSE ONLY.
Required: Yes; Enterable Field: No, Radio_Button
Validation Rules:

Value	Value Description
<5	
6-10	
>10	

Frozen Tissue Banked

Definition:
Required: Yes; Enterable Field: No, Radio_Button
Validation Rules: None

Value	Value Description
YES	
NO	
Unknown	

Warm Ischemic Time

Definition: If tissue was frozen, what was the warm ischemic time (in Minutes).
Required: Yes; Enterable Field: Yes
Validation Rules: None
Data Type: Number; Default Value: -1; Data Range: -1 - 999

Ischemia Time Range (min)

Definition: THIS ELEMENT IS FOR DATA QUERY VIEWER PURPOSE ONLY.
Required: Yes; Enterable Field: No, Radio_Button
Validation Rules:

Value	Value Description
<15	
15-30	
31-60	
>60	

Blood Products Available

Definition: Is there a sample blood or blood product associated with this accession
Required: Yes; Enterable Field: No, Radio_Button
Validation Rules: None

Value	Value Description
Yes	
No	
Unknown	

Accession Available

Definition:
Required: Yes; Enterable Field: No, Radio_Button
Validation Rules: None

Value	Value Description
Yes	
No	

Data on Individual Tissue Blocks and Fluid Aliquots

Block Id (Label)

Definition:
Required: Yes; Enterable Field: Yes
Validation Rules:
Data Type: Number; Default Value: No default; Data Range:

Sample Type

Definition:
Required: Yes; Enterable Field: No, Radio_Button
Validation Rules:

Value	Value Description
Breast	
Lymph Node	
Metastatic Site	
Whole Blood	
Serum	
Plasma	
Buffy Coat	
RBCs	
Urine	
Other	

Procedure Type

Definition:
Required: Yes; Enterable Field: No, Radio_Button

Validation Rules:

Value	Value Description
Biopsy	
Resection	
Tissue Aspiration	
Blood Draw	
Fluid	
Other	
Unknown	
N/A	

Sample Processing

Definition:

Required: Yes; Enterable Field: No, Radio_Button

Validation Rules:

Value	Value Description
Formalin Paraffin	
Other Fixative	
Frozen Bulk	
Frozen OCT	
Unknown	

Freezer Temperature

Definition:

Required: Yes; Enterable Field: No, Radio_Button

Validation Rules:

Value	Value Description
-40	
-40 to -180	
< -180	
Not Applicable	

Invasive Tumor Present

Definition:

Required: Yes; Enterable Field: No, Radio_Button

Validation Rules:

Value	Value Description
Yes	
No	
No - In Situ only	
N/A	
Unknown	

Primary or Metastatic Tumor Present

Definition:

Required: Yes; Enterable Field: No, Radio_Button

Validation Rules:

Value	Value Description
Primary	
Metastatic	
Both	
Neither	
N/A	
Unknown	

Tumor Size, Maximum Diameter (cm)

Definition:

Required: Yes; Enterable Field: Yes
Validation Rules:
Data Type: Number; Default Value: No default; Data Range:

Url to Image

Definition:
Required: No; Enterable Field: Yes
Validation Rules:
Data Type: Number; Default Value: No default; Data Range:

Sample Availability

Definition:
Required: Yes; Enterable Field: No, Combo_Box
Validation Rules:

Value	Value Description
Yes	
No	

Histology Block Type

Definition:
Required: Yes; Enterable Field: No, Radio_Button
Validation Rules:

Value	Value Description
Invasive cancer tissue	
Carcinoma in-situ	
Normal Adjacent	
Normal fatty tissue	
Normal skin tissue	
Normal muscle tissue	
Donor tissue	
N/A - Blood and Fluids	

Therapy Events

Cancer Therapy

Therapy Type

Definition:
Required: Yes; Enterable Field: No, Combo_Box
Validation Rules: None

Value	Value Description
Surgical Resection	
Surgical Biopsy	
Radiation Rx	
Hormonal Rx	
Chemo Rx	
Biologic Response Modifier	
Other Rx	
Unknown	

Months After Diagnosis

Definition:
Required: Yes; Enterable Field: Yes
Validation Rules: None
Data Type: Number; Default Value: -1; Data Range: -1 - 999

Rx Protocol

Definition:
Required: Yes; Enterable Field: No, Combo_Box
Validation Rules: None

Value	Value Description

Cancer Directed Surgery	
Non Cancer Directed Surgery	
External Radiation	
Internal Radiation (Implants)	
Single Agent Chemotherapy	
Multiple Agent Chemotherapy	
Immuno-therapy	
Cryotherapy	
Other	
Unknown (Default)	

Anatomic Location

Definition:

Required: Yes; Enterable Field: No, Radio_Button

Validation Rules: None

Value	Value Description
Primary Site	
Known Metastatic Site	
Site Not Applicable	
Unknown (Default)	

Therapy Events

Definition:

Required: Yes; Enterable Field: Yes

Validation Rules:

Data Type: Number; Default Value: No default; Data Range:
