# Supplementary material for: A Novel Cross-Disciplinary Multi-Institute Approach to Translational Cancer Research: Lessons Learned from Pennsylvania Cancer Alliance Bioinformatics Consortium (PCABC)
Source: Cancer Inform. 2007 Jun 8;3:255–74. (PMC2675833)
Supplement: Melanoma CDEs — (additional files #10) [file cin-03-255-s10.pdf]

Additional File #10

Pennsylvania Cancer Alliance Bioinformatics Consortium (PCABC)

Melanoma Common Data Elements

vMarch 2006

Cancer Centers

Cancer Center

Site Name

Definition:

Required: Yes; Enterable Field: No, Radio_Button

Validation Rules:

Value	Value Description
ACC, UPenn	
FCCC	
KCC, TJU	
PSU	
UPCI	
Wistar	
Geisinger	

Case Identification

Case Identification

PA CA Case Number

Definition: This is a de-identified number given by the submitting institution to index all data on a given case.

Required: Yes; Enterable Field: Yes

Validation Rules: Enter a de-identified number or character string to label this case (patient) in the PA CA Alliance database. This number should not be based on any patient identifier such as name, social security number, etc. The PA CA Alliance database will append a prefix to this number which will identify the tissue bank responsible for this tissue. For example, if a case is identified in Pittsburgh as ABC-1234, the system will save it as PIT-ABC-1234.

Data Type: Character; Default Value: No default; Maximum length: 32

CDE version

Definition: Version 2 (1/15/2004 aap) with NAACR elements

Required: Yes; Enterable Field: Yes

Validation Rules:

Data Type: Number; Default Value: No default; Data Range:

Research Consent Elements

Consent Status

Tissue Consent Status

Definition: Is there a valid consent in place for use of this patient? tissue in research?

Required: Yes; Enterable Field: No, Radio_Button

Validation Rules: None

Value	Value Description
Valid	
Not Valid	
Unknown (Default)	

Data Consent Status

Definition: Is there a valid consent in place for use of this patient?s data in research?
Required: Yes; Enterable Field: No, Radio_Button
Validation Rules: None

Value	Value Description
Valid	
Not Valid	
Unknown (Default)	

Demographics and History

Base Demographics

Age at Diagnosis

Definition: The patient's age when diagnosed with melanoma. Ideally the age at diagnostic biopsy.
Required: Yes; Enterable Field: Yes
Validation Rules: You may have several primary melanomas for one patient. If you are unable to determine if the specimen represents a recurrence or a new primary , considered specimen as a new PRIMARY. Registry has sequence codes to correlate to the case. Ask for Appendix E from PA Cancer Registry if the age at diagnosis is not known within +/- one year, enter a -1.
Data Type: Number; Default Value: -1; Data Range: 10 - 99

Age Range at Diagnosis

Definition: THIS ELEMENT IS FOR DATA QUERY VIEWER PURPOSE ONLY.
Required: Yes; Enterable Field: No, Radio_Button
Validation Rules:

Value	Value Description
0-20	
21-30	
31-40	
41-50	
51-60	
61-70	
71-80	
>80	

Age Range at Diagnosis

Definition: THIS ELEMENT IS FOR DATA QUERY VIEWER PURPOSE ONLY.
Required: Yes; Enterable Field: No, Radio_Button
Validation Rules:

Value	Value Description

Sex

Definition: Code for sex of patient.
Required: Yes; Enterable Field: No, Radio_Button
Validation Rules: None

Value	Value Description
Male	
Female	
Unknown	

Race

Definition: Code the patient?s race. Race is coded separately from Spanish/Hispanic Orgin.
Required: Yes; Enterable Field: No, Radio_Button
Validation Rules: None

Value	Value Description

African American	
Asian	
Native American	
Pacific Islander	
Other	
Unknown (Default)	

Progression and Outcomes

Clinical Staging (at diagnosis)

Clinical Staging, AJCC Version

Definition: A code that indicates the edition of the AJCC manual used to stage the tumor. This applies to the manually coded AJCC fields It does not apply t o Derived AJCC T,N,M and AJCC Stage Group fields.

Required: Yes; Enterable Field: No, Radio_Button

Validation Rules: This is the AJCC Cancer Staging Manual that you are using to determine TNM stage. This is also called the "AJCC Version". Most institutions are using Version 5, however, a new version, version 6, was introduced in late 2002.

Value	Value Description
2nd Edition	
3rd Edition	
4th Edition	
5th Edition (Default)	
6th Edition	
Unknown	

T Stage, Clinical

Definition: Detailed site-specific codes for the clinical tumor (T) as defined by AJCC and recorded by the physician.

Required: Yes; Enterable Field: No, Radio_Button

Validation Rules: See the AJCC Cancer Staging Manual for descriptions of the staging procedure.

Value	Value Description
TX (Default)	
T0	
Tis	
T1	
T1a	
T1b	
T2	
T2a	
T2b	
T3	
T3a	
T3b	
T4	
T4a	
T4b	
Unknown	

N Stage, Clinical

Definition: Detailed site-specific codes for the clinical nodes (N) as defined by AJCC and recorded by the physician.

Required: Yes; Enterable Field: No, Radio_Button

Validation Rules: See the AJCC Cancer Staging Manual for descriptions of the staging procedure.

Value	Value Description
NX (Default)	
N0	
N1	

N2	
N3	
Unknown	

M Stage, Clinical

Definition: Detailed site-specific codes for the clinical metastases (M) as defined by AJCC and recorded by the physician.

Required: Yes; Enterable Field: No, Radio_Button

Validation Rules: See the AJCC Cancer Staging Manual for descriptions of the staging procedure.

Value	Value Description
MX (Default)	
M0	
M1	
M1a	
M1b	
M1c	
Unknown	

cTNM

Definition: THIS ELEMENT IS FOR DATA QUERY VIEWER PURPOSE ONLY.

Required: Yes; Enterable Field: Yes

Validation Rules:

Data Type: Number; Default Value: No default; Data Range:

Pathologic Staging

Pathologic System, AJCC Version

Definition: A code that indicates the edition of the AJCC manual used to stage the tumor. This applies to the manually coded AJCC fields It does not apply t o Derived AJCC T,N,M and AJCC Stage Group fields.

Required: No; Enterable Field: No, Radio_Button

Validation Rules: This is the AJCC Cancer Staging Manual that you are using to determine TNM stage. This is also called the "AJCC Version". Most institutions are using Version 5, however, a new version, version 6, was introduced in late 2002.

Value	Value Description
2nd Edition	
3rd Edition	
4th Edition	
5th Edition (Default)	
6th Edition	
Unknown	

T Stage, Pathologic

Definition: Detailed site-specific codes for the pathological tumor (T) as defined by AJCC and recorded by the physician.

Required: No; Enterable Field: No, Radio_Button

Validation Rules: See the AJCC Cancer Staging Manual for descriptions of the staging procedure.

Value	Value Description
TX	
T0	
Tis	
T1	
T1a	
T1b	
T2	
T2a	
T2b	
T3	
T3a	

T3b	
T4	
T4a	
T4b	
Unknown	

N Stage, Pathologic

Definition: Detailed site-specific codes for the pathological nodes (N) as defined by AJCC and recorded by the physician.

Required: No; Enterable Field: No, Radio_Button

Validation Rules: See the AJCC Cancer Staging Manual for descriptions of the staging procedure.

Value	Value Description
NX	
N0	
N1	
N1a	
N1b	
N2	
N2a	
N2b	
N2c	
N3	
Unknown	

M Stage, Pathologic

Definition: Detailed site-specific codes for the pathological metastases (M) as defined by AJCC and recorded by the physician.

Required: No; Enterable Field: No, Radio_Button

Validation Rules: See the AJCC Cancer Staging Manual for descriptions of the staging procedure.

Value	Value Description
MX	
M0	
M1	
M1a	
M1b	
M1c	
Unknown	

pTNM

Definition: THIS ELEMENT IS FOR DATA QUERY VIEWER PURPOSE ONLY.

Required: Yes; Enterable Field: Yes

Validation Rules:

Data Type: Number; Default Value: No default; Data Range:

Most Recent Followup

Most Recent Follow up (months from diagnosis)

Definition: Date of last contact with the patient, or date of death

Required: Yes; Enterable Field: Yes

Validation Rules: Enter the date of the most follow up in months from diagnosis. For example, if the diagnosis was in December of 1998 and the most recent follow up was January 2001, enter 37. The date of most recent follow up is defined as the date at which have medical information on the patient. For Example: if one is abstracting information from a patient on February 2003 and last medical record (or contact) with the patient is 0ctober 2002, October 2002 should be used to calculate this field. If the patient has died and we have the date of death, it should be used to calculate this field.

Data Type: Number; Default Value: -1; Data Range: -1 - 999

Follow up (Months)

Definition:

Required: Yes; Enterable Field: No, Radio_Button

Validation Rules:

Value	Value Description
<13	
13-24	
25-36	
37-60	
>60	

Vital Status

Vital Status at Most Recent Follow Up

Definition: Vital Status of the patient as of the date entered in item (Date of Last Contact

Required: Yes; Enterable Field: No, Radio_Button

Validation Rules: If the patient was alive at most recent follow up, enter "Yes". If the patient has died enter "No". I can't think of a reason to use "Unknown". The PA CA Alliance system has no element for "lost to follow up". We can calculate how active follow up has been by comparing the value in the "Most Recent Follow Up" element with the current date.

Value	Value Description
Alive	
Dead	
Unknown	

Months to from Diagnosis to Death

Definition:

Required: Yes; Enterable Field: Yes

Validation Rules: If the patient has died and we have a reasonably accurate date of death enter the number of months between diagnosis and death and enter it in this element. Note that this is may be a redundant field as on should be able to derive this from the fields "Most Recent Follow Up" and "Vital Status at Most Recent Follow Up" if the "Most Recent Follow Up" field has been entered correctly. The PA CA Alliance Data Managers should discuss this.

Data Type: Number; Default Value: -1; Data Range: -1 - 999

Post Diagnosis Survival(Months)

Definition:

Required: Yes; Enterable Field: No, Radio_Button

Validation Rules:

Value	Value Description
<13	
13-24	
25-36	
37-60	
>60	

Follow up

Definition:

Required: Yes; Enterable Field: Yes

Validation Rules:

Data Type: Number; Default Value: No default; Data Range:

First Recurrence

Months from Diagnosis to First Recurrence

Definition: Months between diagnosis and first recurrence

Required: Yes; Enterable Field: Yes

Validation Rules: Enter the number of months from Diagnosis to First Reported Recurrence. One should use the first recurrence of the primary tumor. Other "recurrences" should be considered "progressions" and be entered in the "Distant Metastatic Progression" Element. This is a somewhat controversial field. One can imaging a patient with a primary tumor, a recurrence, a remission and then a second recurrence. To avoid complexity, we have set up the system so that one patient can have one first recurrence. The data managers should discuss this. If the patient is "never disease free", Months from Diagnosis to First Recurrence should be entered as "0".

Data Type: Number; Default Value: -1; Data Range: 1 - 999

First Recurrence Type

Definition: Code for the type of first recurrence after a period of documented disease-free intermission or remission.

Required: Yes; Enterable Field: No, Combo_Box

Validation Rules: This element is taken from the NAACCR Data Dictionary (item 1880). Enter the type of first recurrence. If there is first reported recurrence is of more than one type, enter the most "severe" type (Distant > Regional > Local, Tissue > Lymph Node). If the patient was never disease free after initial therapy (for example, positive margins or the presence of mets) this should so indicated. If the patient is "never disease free", Months from Diagnosis to First Recurrence should be entered as "0".

Value	Value Description
None - disease free	
In situ	
Local	
Regional - NOS	
Regional - Tissue	
Regional - Lymph Nodes	
Distant	
Never Disease Free	
Recurred - Site unknown	
Unknown (Default)	

Diagnosis To First Recurrence(Months)

Definition:

Required: Yes; Enterable Field: No, Radio_Button

Validation Rules:

Value	Value Description
<13	
13-24	
25-36	
37-60	
>60	

Recurrence Events

Definition:

Required: Yes; Enterable Field: Yes

Validation Rules:

Data Type: Number; Default Value: No default; Data Range:

Overall Metastatic Progression

First Distant Metastasis

Definition: Code for the distant site in which the tumor has recurred.

Required: Yes; Enterable Field: No, Combo_Box

Validation Rules: Enter the location of the distant metastasis. Note that a distant metastasis may be present at diagnosis, as a "first recurrence" or as part of a later disease progression.

Value	Value Description
No First Location	
Peritoneum	
Lung	
Pleura	
Liver	
Bone	
CNS	
Skin	
Distant Lymph Nodes	
Other - Generalized - NOS	

Second Distant Metastasis

Definition: Code for the distant site in which the tumor has recurred.

Required: Yes; Enterable Field: No, Combo_Box

Validation Rules: Enter the location of the distant metastasis. Note that a distant metastasis may be present at diagnosis, as a "first recurrence" or as part of a later disease progression.

Value	Value Description
No Second Location	
Peritoneum	
Lung	
Pleura	
Liver	
Bone	
CNS	
Skin	
Distant Lymph Nodes	
Other - Generalized - NOS	

Third Distant Metastasis

Definition: Code for the distant site in which the tumor has recurred.

Required: Yes; Enterable Field: No, Combo_Box

Validation Rules: Enter the location of the distant metastasis. Note that a distant metastasis may be present at diagnosis, as a "first recurrence" or as part of a later disease progression.

Value	Value Description
No Third Location	
Peritoneum	
Lung	
Pleura	
Liver	
Bone	
CNS	
Skin	
Distant Lymph Nodes	
Other - Generalized - NOS	

Time Line

Overall TL

Definition: THIS ELEMENT IS FOR DATA QUERY VIEWER PURPOSE ONLY.

Required: Yes; Enterable Field: Yes

Validation Rules:

Data Type: Number; Default Value: No default; Data Range:

Therapy TL

Definition: THIS ELEMENT IS FOR DATA QUERY VIEWER PURPOSE ONLY.

Required: Yes; Enterable Field: Yes

Validation Rules:

Data Type: Number; Default Value: No default; Data Range:

Procedure TL

Definition: THIS ELEMENT IS FOR DATA QUERY VIEWER PURPOSE ONLY.

Required: Yes; Enterable Field: Yes

Validation Rules:

Data Type: Number; Default Value: No default; Data Range:

Tissue Accession Data

Date and Procedure Elements

De-identified Accession ID

Definition: De-identified accession ID from submitting institution

Required: Yes; Enterable Field: Yes

Validation Rules: This is a de-identified number, chosen by the submitting institution, to label this accession. The system will append a prefix designating the submitting institution. For example, if one is at PITT and enters 12345 the system will designate the accession as PIT-12345.

Data Type: Character; Default Value: No default; Maximum length: 32

Months between Dx and Accession

Definition:
Required: Yes; Enterable Field: Yes
Validation Rules:
Data Type: Number; Default Value: No default; Data Range:

Procedure Type

Definition: Site-specific codes for the type of surgery to the primary site performed. Records the surgical removal of distant lymph nodes or other tissues/organs beyond primary site.
Required: Yes; Enterable Field: No, Combo_Box
Validation Rules: None

Value	Value Description
Blood or Fluid Only	
Shave Biopsy of Skin	
Punch Biopsy of Skin	
Excisional Biopsy of Skin	
Wide Excision	
Lymph Node Exploration Only	
Exploration of Distant Mets	
Other	
Unknown (Default)	

Procedure Site

Definition: Primary site of surgery associated with this accession
Required: Yes; Enterable Field: No, Combo_Box
Validation Rules: None

Value	Value Description
Head and Neck	
Upper Extremity	
Lower Extremity	
Back	
Chest and Abdomen	
Ocular	
Subungual	
Mucosal	
Anogenital (non mucosal)	
Other	
Not Applicable	
Unknown (Default)	

Diagnosis To Accession(Months)

Definition:
Required: Yes; Enterable Field: No, Radio_Button
Validation Rules:

Value	Value Description
<13	
13-24	
25-36	
37-60	
>60	

Procedure Events

Definition:
Required: Yes; Enterable Field: Yes
Validation Rules:
Data Type: Number; Default Value: No default; Data Range:

Diagnosis and Grade

Invasive Tumor Present

Definition: Was invasive tumor identified at the accession?

Required: Yes; Enterable Field: No, Radio_Button

Validation Rules: If this accession (and of its specimens) contains any invasive melanoma one should enter "Yes". If there is no melanoma (invasive or in situ) one should enter "No". Should the accession show in situ melanoma without invasive melanoma use the option "In situ Only". Cases of normal skin or control skin, without melanoma, should be entered as "No". The "Not Applicable option is used of accessions such as blood samples only

Value	Value Description
Yes	
No	
In Situ Only	
NA	
Unknown	

Primary or Metastatic Tumor

Definition: Was the tumor primary or metastatic?

Required: Yes; Enterable Field: No, Radio_Button

Validation Rules: If the accession contains only the primary melanoma, enter "primary". If the accession contains only metastatic melanoma, enter "metastatic". Should the accession contain both primary and metastatic melanoma samples (for example, a primary site with region mets) enter "both". The "Not Applicable option is used of accessions such as blood samples only

Value	Value Description
Primary	
Metastatic	
Both	
Neither	
Not Applicable	
Unknown	

Primary Histology

Definition: Codes for the histological type of the tumor being reported using ICD-O-3.

Required: Yes; Enterable Field: No, Combo_Box

Validation Rules: Choose the primary histology in the current accession. Note that some rare types of melanoma are not listed and should be entered as "Other Melanoma Type". The option "Not Applicable" is used for cases in which there is no histology, such as accessions that represent a blood sample only.

Value	Value Description
Melanoma in situ	
Malignant Melanoma NOS	
Superficial Spreading	
Nodular	
Lentigno Maligna Melanoma	
Acral Lentiginous	
Desmoplastic	
Nevoid	
Spitzoid	
Other Melanoma Type	
No Melanoma Seen	
Not Applicable	
Unknown (Default)	

Tumor Size and Extent

Max Surface Diameter (mm)

Definition: Largest dimension, or the diameter of the primary tumor in millimeters.

Required: Yes; Enterable Field: Yes

Validation Rules: None

Data Type: Number; Default Value: -1; Data Range: 0 - 50

Clark's Level

Definition: Clark's Level

Required: Yes; Enterable Field: No, Radio_Button

Validation Rules: Not Applicable is used for cases with no histology, like fluid only accessions.

Value	Value Description
1	1 - Above basal lamina (in situ melanoma)
2	2 - Extension into papillary dermis
3	3 - Interface papillary-reticular dermis
4	4 - Reticular dermis
5	5 - Subcutaneus fat
Not Applicable	Not Applicable - used for cases with no histology, like fluid only accessions.
Unknown	

Breslow's Thickness (mm)

Definition: Breslow's thickness in mm

Required: Yes; Enterable Field: Yes

Validation Rules: The Breslow's Thickness is measured from the granular layer to the deepest invasion of the tumor.

Data Type: Number; Default Value: -1; Data Range: 0 - 50

Tumor Size Range (mm)

Definition:

Required: Yes; Enterable Field: No, Radio_Button

Validation Rules:

Value	Value Description
<0.5 x 10	
0.5-1.0 x 10	
1.1-1.5 x 10	
1.6-2.0 x 10	
>2.0 x 10	
Unknown	

Tumor findings and Attributes

Surface Ulceration

Definition: Is gross or microscopic surface ulceration evident

Required: Yes; Enterable Field: No, Radio_Button

Validation Rules: Enter "Yes" if there is documentation of any ulceration, gross or microscopic. "Not Applicable" is used for cases with no histology, like fluid only accessions.

Value	Value Description
Yes	
No	
Not Applicable	Not Applicable - used for cases with no histology, like fluid only accessions.
Unknown	

Angiolymphatic Invasion

Definition: Is there histologic evidence of angiolymphatic invasion in this accession?

Required: Yes; Enterable Field: No, Radio_Button

Validation Rules: Enter "Yes" if there is documentation of any angiolymphatic (or vascular-lymphatic) invasion by the tumor. Not Applicable is used for cases with no histology, like fluid only accessions.

Value	Value Description
Yes	Not Applicable - used for cases with no histology, like fluid only accessions.
No	
Not Applicable	
Unknown	

Pre-existing Nevus

Definition: Is there histologic evidence of a pre-existing nevus?

Required: Yes; Enterable Field: No, Radio_Button

Validation Rules: Enter "Yes" if there is documentation of any pre-existing nevus at the site of the primary tumor. Not Applicable is used for cases that do not have primary tumor or cases with no histology, like fluid only accessions.

Value	Value Description
Yes	
No	
Not Applicable	Not Applicable is used for cases that do not have primary tumor or cases with no histology, like fluid only accessions.
Unknown	

Intransit or Satellite Lesions

Definition: Is there gross or microscopic evidence of in transit or satellite metastases?

Required: Yes; Enterable Field: No, Radio_Button

Validation Rules: Enter "Yes" if there is any evidence (gross or microscopic) intransit or satellite lesions. Not Applicable is used for cases with no histology, like fluid only accessions.

Value	Value Description
Yes	
No	
Not Applicable	Not Applicable is used for cases with no histology, like fluid only accessions.
Unknown	

Evidence of Regression

Definition: Is there histologic evidence of regression

Required: Yes; Enterable Field: No, Radio_Button

Validation Rules: None

Value	Value Description
Yes	
No	
Not Applicable	
Unknown	

Tumor Infiltrating Lymphocytes

Definition: What is the intensity of the lymphoid infiltrate in the tumor?

Required: Yes; Enterable Field: No, Radio_Button

Validation Rules: None

Value	Value Description
Absent	
Non-brisk	
Moderate	
Brisk	
Not Applicable	
Unknown	

Mitotic Rate (# / 10 HPF)

Definition: Number of mitotic figures per 10 HPF

Required: Yes; Enterable Field: No, Radio_Button

Validation Rules: IF it is not in 10 high power fields, do not enter.

Value	Value Description
0	
1 - 2	
3 - 5	
6 - 10	
11 -15	
> 15	
Not Applicable	

Unknown	

Perineural Invasion

Definition: Is there histologic evidence of perineural invasion by tumor?
Required: Yes; Enterable Field: No, Radio_Button
Validation Rules: None

Value	Value Description
Yes	
No	
Not Applicable	
Unknown	

Increased Vascularity

Definition: What is the state of the vascularity surrounding the tumor?
Required: Yes; Enterable Field: No, Radio_Button
Validation Rules: None

Value	Value Description
Yes	
No	
Not Applicable	
Unknown	

Lymph Node Examination

Sentinel Lymph Biopsy Results

Definition: Was the sentinel lymph node involved?
Required: Yes; Enterable Field: No, Radio_Button
Validation Rules: None

Value	Value Description
Positive	
Negative	
N/A - No Sentinel Mapping	
Unknown	

Total Lymph Nodes Examined

Definition: Total number of lymph nodes examined at this accession
Required: Yes; Enterable Field: Yes
Validation Rules: None
Data Type: Number; Default Value: -1; Data Range: -1 - 99

Lymph Node Examined Range

Definition: THIS ELEMENT IS FOR DATA QUERY VIEWER PURPOSE ONLY.
Required: Yes; Enterable Field: No, Radio_Button
Validation Rules:

Value	Value Description
0	
1-5	
>5	
Unknown	

Total Lymph Nodes Positive

Definition: Identifies regional lymph nodes positive in this accession. Add Sentinel and Regional Lymph Nodes
Required: Yes; Enterable Field: Yes
Validation Rules: None
Data Type: Number; Default Value: -1; Data Range: -1 - 99

Lymph Node Positive Range

Definition: THIS ELEMENT IS FOR DATA QUERY VIEWER PURPOSE ONLY.
Required: Yes; Enterable Field: No, Radio_Button
Validation Rules:

Value	Value Description
0	
1-5	
>5	
Unknown	

Complete Dissection Performed?

Definition: Was a complete lymph node dissection performed?
Required: Yes; Enterable Field: No, Radio_Button
Validation Rules: None

Value	Value Description
Yes	
No	
Not Applicable	
Unknown	

Largest Nodal Metastasis

Definition: The diameter of the largest lymph node metastatasis
Required: Yes; Enterable Field: No, Radio_Button
Validation Rules: None

Value	Value Description
< 2 mm	
>= 2 mm	
Not Applicable (no mets)	
Unknown	

Extranodal Extension

Definition: Was there gross matting or microscopic evidence of extracapsular extension in lymph nodes?
Required: Yes; Enterable Field: No, Radio_Button
Validation Rules: None

Value	Value Description
Yes	
No	
Not Applicable (no mets)	
Unknown	

Lymph Node Ratio

Definition:
Required: Yes; Enterable Field: Yes
Validation Rules:
Data Type: Number; Default Value: No default; Data Range:

Tissue Processing

Total Number of Paraffin Blocks

Definition: Total number of paraffin blocks associated with this accession
Required: Yes; Enterable Field: Yes
Validation Rules: None
Data Type: Number; Default Value: -1; Data Range: -1 - 99

Paraffin Block Range

Definition: THIS ELEMENT IS FOR DATA QUERY VIEWER PURPOSE ONLY.
Required: Yes; Enterable Field: No, Radio_Button
Validation Rules:

Value	Value Description
<5	
6-10	
>10	

Frozen Tissue Available

Definition: Was frozen tissue banked on this accession?

Required: Yes; Enterable Field: No, Radio_Button

Validation Rules: None

Value	Value Description
Yes	
No	
Not Applicable	
Unknown	

Warm Ischemic Time (min)

Definition: Number of minutes between removal of the tissue and freezing of the tissue

Required: Yes; Enterable Field: Yes

Validation Rules: None

Data Type: Number; Default Value: -1; Data Range: -1 - 999

Ischemia Time Range(min)

Definition: THIS ELEMENT IS FOR DATA QUERY VIEWER PURPOSE ONLY.

Required: Yes; Enterable Field: No, Radio_Button

Validation Rules:

Value	Value Description
<15	
15-30	
31-60	
>60	

Are Blood Products Available

Definition: Where blood samples banked associated with this accession?

Required: Yes; Enterable Field: No, Radio_Button

Validation Rules: None

Value	Value Description
Yes	
No	
Not Applicable	
Unknown	

Is this accession currently available?

Definition: Is this accession currently available for research?

Required: Yes; Enterable Field: No, Radio_Button

Validation Rules: None

Value	Value Description
Yes	
No	

Data on Individual Tissue Blocks and Fluid Aliquots

Block ID (label)

Definition: Alphanumeric string that identifies the block or fluid in question. This string must not identify the patient

Required: Yes; Enterable Field: Yes

Validation Rules: None

Data Type: Character; Default Value: No default; Maximum length: 10

Sample Type

Definition:

Required: Yes; Enterable Field: No, Radio_Button

Validation Rules: None

Value	Value Description

Skin	
Lymph Node	
Metastatic Site	
Whole Blood (Default)	
Serum	
Plasma	
Buffy Coat	
RBC	
Other	

Procedure Type

Definition:

Required: Yes; Enterable Field: No, Radio_Button

Validation Rules: None

Value	Value Description
Biopsy	
Resection	
Tissue Aspiration	
Blood Draw	
Fluid	
Other	
Not Applicable	
Unknown (Default)	

Sample Processing

Definition:

Required: Yes; Enterable Field: No, Radio_Button

Validation Rules: None

Value	Value Description
Formalin Paraffin	
Other Fixed	
Bulk Frozen	
OCT Frozen	
Unknown	

Block Tumor Size (mm)

Definition:

Required: Yes; Enterable Field: Yes

Validation Rules: None

Data Type: Number; Default Value: -1; Data Range: -1 - 5.00

Invasive Tumor Present

Definition:

Required: Yes; Enterable Field: No, Radio_Button

Validation Rules: None

Value	Value Description
Yes	
No	
In Situ Only	
Not Applicable	
Unknown	

Primary or Metastatic Tumor Present

Definition: Is tumor in this sample primary or metastatic

Required: Yes; Enterable Field: No, Radio_Button

Validation Rules: None

Value	Value Description
Primary	
Metastatic	

Both	
Neither	
Not Applicable	
Unknown (Default)	

Freezer

Definition:
Required: Yes; Enterable Field: No, Radio_Button
Validation Rules: None

Value	Value Description
-40	
-40 to -180	
<180 (Default)	
Not Applicable	

Warm Ischemic Time (min)

Definition:
Required: Yes; Enterable Field: Yes
Validation Rules: None
Data Type: Number; Default Value: -1; Data Range: -1 - 100

URL to Image

Definition:
Required: No; Enterable Field: Yes
Validation Rules: None
Data Type: Character; Default Value: No default; Maximum length: 128

Sample Comment

Definition:
Required: No; Enterable Field: Yes
Validation Rules: None
Data Type: Character; Default Value: No default; Maximum length: 128

Sample Availability

Definition: Is this sample available now?
Required: Yes; Enterable Field: No, Radio_Button
Validation Rules: None

Value	Value Description
Yes	
No (Default)	

Therapy Events

Cancer Therapy

Therapy Type

Definition:
Required: Yes; Enterable Field: No, Combo_Box
Validation Rules: None

Value	Value Description
Surgical Resection	
Radiation Rx	
Chemo Rx	
Biologic Response Modifier	
Other Rx	
Unknown	

Months After Diagnosis

Definition:
Required: Yes; Enterable Field: Yes
Validation Rules: None
Data Type: Number; Default Value: -999; Data Range: -999 - 999

Rx Protocol

Definition:
Required: Yes; Enterable Field: No, Combo_Box
Validation Rules: None

Value	Value Description
Cancer Directed Surgery	
Non Cancer Directed Surgery	
External Radiation	
Internal Radiation (Implants)	
Single Agent Chemotherapy	
Multiple Agent Chemotherapy	
Immuno-therapy	
Cryotherapy	
Other	
Unknown	

Anatomic Location

Definition:
Required: Yes; Enterable Field: No, Radio_Button
Validation Rules: None

Value	Value Description
Primary Site	
Known Metastatic site	
Site Not Applicable	
Unknown	

Therapy Events

Definition:
Required: Yes; Enterable Field: Yes
Validation Rules:
Data Type: Number; Default Value: No default; Data Range:
